# Regional disparities in US media coverage of archaeology research

**DOI:** 10.1126/sciadv.adt5435

**Published:** 2025-07-02

**Authors:** Bridget Alex, Jenny Ji, Rowan Flad

**Affiliations:** ^1^ Department of Human Evolutionary Biology, Harvard University, Cambridge, MA, USA.; ^2^ SAPIENS Magazine, New York, NY, USA.; ^3^ Department of Bioengineering, Stanford University, Stanford, CA, USA.; ^4^ Department of Anthropology, Harvard University, Cambridge, MA, USA.

## Abstract

Mass media represents a primary avenue for research to reach diverse publics, but relatively few peer-reviewed scientific papers become popular science news. Numerous gatekeepers determine which research manuscripts complete this dissemination pathway, and the resulting media landscape influences public understandings of scientific fields. Here, we compare scientific and popular publishing of archaeology about different geographic regions. Of 1155 archaeology papers in one specialist and six general science journals across 6 years, 32% were reported by at least one of 15 US news sources. Mixed-effects logistic regression models revealed variation across news sources, but overall papers about archaeology in United Kingdom, Israel/Palestine, and Australia were significantly more likely to receive coverage, compared to China/Taiwan. This disparity raises concerns as archaeology influences notions of identity and cultural achievement, and has been misappropriated by racist, nationalist ideologies. We recommend ways for actors in research dissemination to diversify archaeology coverage.

## Teaser

US media coverage of archaeology shows geographic disparities, with archaeology of China/Taiwan less likely to receive coverage.

## INTRODUCTION

As the primary means for scholars to communicate their research, peer-reviewed journal articles remain the “currency of science” (*1*). The number of papers published annually has been growing for over a century (*2*), reaching at least 2.9 to 4.7 million articles worldwide in 2020 (*3*, *4*). Despite the profusion of publications, much of this research is inaccessible to the public due, in part, to paywalls, low readability (*5*), and the specialized knowledge needed to grasp the significance.

One remedy to these barriers, mass media produces stories about science that may be consumed by millions of individuals belonging to diverse publics. According to surveys by Pew Research Center, about 75% of US adults are interested in following news about science, a higher level of interest than business, finance, sports, or entertainment (*6*). A specialized form of journalism professionalized in the second half of the 20th century, science journalism primarily covers science, medicine, and technology, including scientific achievements, the scientific process, and stories of scientists’ careers and research (*7*, *8*). Science journalists have varied backgrounds: Some have studied journalism or science journalism, completed graduate degrees in the sciences or medicine, and/or entered the profession through internships or self-publication. The workforce includes editors who oversee a publication’s content, staff reporters/writers employed at a media organization, and freelancers who work by contract for any number of outlets (*9*). In addition to disseminating and translating research to the public, science journalists also investigate and critique science, helping to hold scientists accountable to society (*7*, *10*).

It is clear, however, that mass media reports a slim portion of scholarly research, although quantified estimates are rare. In analysis of scholarly publications in the 2010s including more than 670,000 journal articles, Yu and colleagues found that ~6% received any press coverage (*11*). That rate varied considerably by field, with 53% of the reported research concerning health and medicine. In deciding which research to cover, press editors rely on factors that constitute a story’s news value, which include timeliness, conflict, unexpectedness, novelty, and composition, or the diversity of story types covered by an outlet at one time [e.g., (*12*–*14*)] (text S1). Proximity, another classic news factor, can be understood as the closeness between audience and event in physical, geographical terms and the psychological distance based on cultural, political, or economic ties (*15*, *16*). Within science journalism, additional factors are considered such as scientific relevance, which is often signaled by the publishing journal’s prominence or an accompanying press release (*17*).

A study’s news value does not necessarily match its scientific value, and news editors make subjective decisions over which research merits the media spotlight. While the media filter may distort scientific fields, it is the press’s version that largely shapes public perceptions of science. For example, examining press coverage of research in bioarchaeology (a specialization of archaeology that focuses on human remains), Stojanowski and Duncan (*18*) found nonalignment between the subfield’s emerging research foci and stories in the news aggregator *Science Daily*, which predominately reported on mummies, Vikings, natural curiosities, superlative discoveries, and famous individuals such as Richard III. More broadly, science journalism tends to prioritize superlative findings (oldest, smallest, etc.), “firsts,” and breakthroughs, rather than presenting science as an ongoing process of replication and incremental progress (*19*, *20*). Decisions by journalists also influence the public image of scientists and scholars. Studies have found that when covering a research paper, US and UK news stories are less likely to mention or quote coauthors with East Asian–sounding names compared to those with Anglo-sounding names (*21*, *22*). In surveys that ask Americans to name a living scientist, the most common responses are individuals who create public content and/or receive considerable publicity, including Anthony Fauci, Neil DeGrasse Tyson, Bill Nye, and Jane Goodall (*23*).

Skewed coverage may be particularly charged for archaeology, a field with large public interest that generates understandings about past cultures with real or imagined descendants. When mischaracterized, archeological data can fuel harmful ideologies. For example, pseudoscience about ancient aliens or lost civilizations attempts to promote white supremacy and negate Indigenous achievements (*24*–*26*). Far-right white supremacist figures have explicitly praised pseudoarchaeology, such as Graham Hancock’s *Ancient Apocalypse* series, as an effective way to spread their ideology (*27*). More subtly, we contend that news coverage prioritizing certain regions or cultural identities may create a false impression that only those ancestries made great achievements in the past. Unreported or under-reported regions may be perceived as “primitive” or lacking hallmarks of civilization.

As an anecdotal example, in the same week of 2021, two major archeological finds were announced by researchers working in Egypt and China. One of these findings, related to the city of Aten of Ancient Egypt’s 18th Dynasty, received considerable attention in US media [e.g., (*28*–*30*)]. The other, sacrificial pits containing precious objects at the Chinese site of Sanxingdui (*31*), was widely covered by Chinese press (*32*–*35*), but not reported in US media at the time. In an op-ed in *The Washington Post*, Flad (*36*) argued that this discrepancy presents an example of anti-Asian biases in US media coverage of archaeology.

Here, as researchers engaged in archaeology, journalism, and statistical analysis, we evaluate and broaden that claim by investigating whether US media coverage of archeological research papers has differed on the basis of geographic location of the findings. Considering archaeology papers published in seven scientific journals from 2015 to 2020, we collated metrics of media attention and coded the research papers by potentially influential variables including geographic region (data S1). These data present a 6-year view of archeological scholarship in major journals and associated global and US media attention. Of 1155 archaeology research papers published in one specialist and six general science journals, 32% were reported by at least one of 15 major US news outlets. Coverage correlated with predictable variables including the publication journal, inclusion in a press release service, and focus on paleolithic archaeology. Geographic focus of the article also likely affected media attention based on results of mixed-effects logistic regression models: Relative to China/Taiwan, articles about archaeology in Israel/Palestine, United Kingdom, and Australia were about three times (2.7, 3.0, and 4.2 times, respectively) more likely to be reported by at least one US news outlet. Applying the analysis to each news source individually, archaeology studies about Egypt, Spain, Turkey, and United States were also significantly more likely to be reported by some outlets compared to China/Taiwan. The findings point to actionable measures for researchers, press office workers, journalists, and news editors to enhance the geographic and cultural diversity of archaeology reported in US press.

## RESULTS

### Data collection

Data were collected from Altmetric (Altmetric.com), a commercial website that tracks and analyzes altmetrics, measures of online activity surrounding research outputs, which include journal articles, datasets, white papers, and more (*37*). Altmetric aggregates hyperlinks or references to research outputs found within other online sources such as news websites, social media platforms, Wikipedia, and other journal articles. For each research output, Altmetric calculates an attention score, which is a weighted approximation of online attention a research output has received. Concerns have been raised about the quality of altmetrics in general provided by Altmetric, PlumX, or other services (*37*–*39*), and studies have demonstrated issues in particular with the service Altmetric for sources such as scholarly books (*40*) and non-English research (*41*). However, Altmetric has been shown to provide relatively reliable counts for English news mentions of peer reviewed journal articles, which are the focus of this study (*38*). Moreover, it is unlikely that documented errors in Altmetric’s news mention data (*42*) would bias results for the questions posed in this study.

We searched for research outputs under the subject area filter “archaeology” published between 2015 and 2020 in one specialist journal, *Antiquity*, and six general science journals: *Science*, *Nature*, *Science Advances*, *Nature Communications*, *Scientific Reports,* and *Proceedings of the National Academy of Sciences* (*PNAS*) (data S2). We focused on these journals because they have relatively high prestige rankings (impact factor and SCImago Journal Ranking), report archaeology research, and generate press releases to promote their articles to the media. Among archaeology-focused journals with similar rankings (*43*), *Antiquity* was selected because it covers diverse methodologies and global archaeology. As a journal with press releases and articles regularly reported by mass media, *Antiquity* contributes to the pool of potential science news most accessible to journalists, just as the six general science journals do. A priori, we did not expect meaningful differences between our results for the specialist or general science journals. After we manually culled the outputs to include only peer-reviewed research about archaeology, our search produced a dataset of 1155 research papers (data S1).

For this dataset, we collated metrics provided by Altmetric including the attention score, global news mentions, and whether a press release appeared on EurekAlert!, a nonprofit news-release platform run by the American Association for the Advancement of Science (AAAS). The platform hosts press releases that meet its eligibility guidelines for institutions including university press offices, journal publishers, and corporations that have paid its submission fee (www.eurekalert.org/about-us). The public can access these releases for free. Journalists can access embargoed releases and research articles before their public release. We also calculated an original metric called the US news score, which was the sum of a research paper’s mentions in 15 major US media outlets or aggregators that cover science: *Yahoo*, *Google*, *MSN*, *The New York Times*, *The Washington Post*, *FOX*, *ABC*, *CNN*, *National Geographic*, *Discover*, *New Scientist*, *Newsweek*, *Scientific American*, *Forbes*, and *Los Angeles Times* (data S1and S3).

We reviewed research papers to code variables including the study’s geographic location, absolute time span, and whether it concerned paleolithic archaeology, under the assumption that this research on humanity’s shared origins and evolution would have global relevance, regardless of its geographic location. Geographic location referred to the present-day country (or countries), subcontinent(s), and continent(s) where the past people or finds are now located; it did not reflect the institutional affiliations of the scientists who authored the study [e.g., (*44*)]. In geographic analyses at each level, we considered articles focused on a single geographic region. Disagreements or ambiguities in coding were resolved by consensus. We checked for interobserver replicability by discussing ambiguous cases, and then, one of us cleaned the full dataset to ensure consistency.

Mixed-effects logistic regression models were generated to calculate the likelihood that articles focused on a given region received US news coverage (US news score > 0) or coverage by a particular news source, controlling for potential confounding variables such as paleolithic archaeology, journal, publication year, and EurekAlert! press release. Regularly used in computational archaeology [e.g., (*45*)], mixed-effects logistic regression models elucidate complex relationships by assessing the influence of a factor on the likelihood of the outcome while holding all other covariates constant. This method is preferred to alternatives because it can model binary outcomes (i.e., whether the article has US news coverage or not) from a mixture of continuous and categorical predictor variables (i.e., journal, year, inclusion in EurekAlert!, paleolithic archaeology, and geographic region).

### Research outputs and media coverage

Between 2015 and 2020, the seven journals collectively published 1155 peer-reviewed papers about archaeology research. There is an upward trend in the number of archaeology papers in these journals, with 109 during 2015 more than doubling to 244 by 2020 (fig. S1 and table S1). Across the 6-year period, more than half (611 of 1155) of the papers appeared in *Antiquity*. *Scientific Reports*, and *PNAS* published more than 100 archaeology papers, whereas the remaining four journals published around 50 or fewer archaeology papers (Table 1).

**Table 1.** Archaeology research papers published and reported by US news sources. Coverage indicates the number of published research papers that were reported by at least one of 15 US news sources (US news score > 0). The % indicates the papers with coverage divided by the total research papers published for a respective row variable. Journal impact factors (in parentheses following the journal name) are the 5-year impact factors reported by journals in 2022. NA, not applicable.

		Paleolithic archaeology			Not paleolithic archaeology			Total articles		
		***N*** ** = 948**			***N*** ** = 207**			***N*** ** = 1155**		
**Journal**		**Coverage**	**Total**	**(%)**	**Coverage**	**Total**	**(%)**	**Coverage**	**Total**	**(%)**
*Antiquity* (2)		9	72	13%	103	539	19%	112	611	18%
*Scientific Reports* (4.9)		17	48	35%	28	176	16%	45	224	20%
*PNAS* (12)		15	27	56%	58	123	47%	73	150	49%
*Science Advances* (15.4)		8	8	100%	29	43	67%	37	51	73%
*Science* (54.4)		21	21	100%	25	29	86%	46	50	92%
*Nature* (60.9)		25	25	100%	16	17	94%	41	42	98%
*Nature Comm*. (17)		4	6	67%	9	21	43%	13	27	48%
Total		99	207	48%	268	948	28%	367	1155	32%
**Continents**		**Coverage**	**Total**	**(%)**	**Coverage**	**Total**	**(%)**	**Coverage**	**Total**	**(%)**
Asia		44	83	53%	72	283	25%	116	366	32%
Europe		21	64	33%	64	273	23%	85	337	25%
Africa		21	35	60%	22	86	26%	43	121	36%
North America		3	8	38%	48	109	44%	51	117	44%
South America		0	0	NA	24	61	39%	24	61	39%
Australia, Oceania		3	3	100%	10	33	30%	13	36	36%
Global		1	5	20%	5	42	12%	6	47	13%
Total*		93	198	47%	245	887	28%	367	1085	34%
**Subcontinents**		**Coverage**	**Total**	**(%)**	**Coverage**	**Total**	**(%)**	**Coverage**	**Total**	**(%)**
Western Europe		17	43	40%	52	205	25%	69	248	28%
Southwest Asia		11	20	55%	31	102	30%	42	122	34%
East Asia		14	21	67%	15	85	18%	29	106	27%
Sub-Saharan Africa		18	30	60%	15	53	28%	33	83	40%
North America		2	7	29%	24	66	36%	26	73	36%
Southeast Asia		11	17	65%	15	49	31%	26	66	39%
Central Asia		6	22	27%	11	41	27%	17	63	27%
South America		0	0	NA	24	61	39%	24	61	39%
Eastern Europe		3	9	33%	4	38	11%	7	47	15%
Central America		1	2	50%	21	38	55%	22	40	55%
North Africa		3	4	75%	8	31	26%	11	35	31%
Australia, Oceania		3	3	100%	9	32	28%	12	35	34%
Total*		89	178	50%	229	801	29%	318	979	32%
**Countries**		**Coverage**	**Total**	**(%)**	**Coverage**	**Total**	**(%)**	**Coverage**	**Total**	**(%)**
China/Taiwan		9	14	64%	13	72	18%	22	86	26%
USA		2	7	29%	19	48	40%	21	55	38%
UK		0	3	0%	20	50	40%	20	53	38%
Italy		3	8	38%	8	39	21%	11	47	23%
Israel/Palestine		5	8	63%	10	29	34%	15	37	41%
Spain		6	13	46%	5	23	22%	11	36	31%
France		5	14	36%	2	19	11%	7	33	21%
Russia		5	14	36%	4	12	33%	9	26	35%
Turkey		0	0	NA	6	23	26%	6	23	26%
Iran		1	6	17%	3	15	20%	4	21	19%
Peru		0	0	NA	9	21	43%	9	21	43%
South Africa		6	12	50%	2	9	22%	8	21	38%
Egypt		0	0	NA	6	20	30%	6	20	30%
Indonesia		7	7	100%	2	8	25%	9	15	60%
Australia		2	2	100%	7	13	54%	9	15	60%
Greece		2	2	100%	1	12	8%	3	14	21%
Total*		53	110	48%	117	413	28%	170	523	33%

*Total differs by geographic level because only articles concerning a single region were included in analysis. For example, multi-country articles were included from the country count but might be included at higher levels if all countries belonged to the same subcontinent or continent.

The Altmetric attention scores ranged from 0 to 3433 (mean of 200; median of 37) (data S1). Altmetric’s generated total news mentions, a measure of global media attention, ranged from 0 to 449 (mean of 18.4; median of 1, mode of 0). Of the articles, 51.7% had a news mention greater than 0, meaning at least one news outlet published a story linking to the study. The five articles with the highest total news mentions concerned paleolithic archaeology (Table 2 and table S2). The US news score was strongly correlated with the Altmetric attention score [coefficient of determination (*R*^2^) = 0.7173) and total news mentions (*R*^2^ = 0.7142) (fig. S2).

**Table 2.** Comparison of research papers receiving top scores for Altmetric global news mentions. BCE, before the common era.

Altmetric global news mentions				
All articles				
**Rank**	**Citation**	**Location**	**Score**	**Eurek Alert!**
1	The earliest modern humans outside Africa. *Science*	Israel/Palestine	449	✔
2	Human origins in a southern African palaeo-wetland and first migrations. *Nature*	Botswana, South Africa	375	
3	The age of the hominin fossils from Jebel Irhoud, Morocco, and the origins of the Middle Stone Age. *Nature*	Morocco	356	✔
4	U-Th dating of carbonate crusts reveals Neanderthal origin of Iberian cave art. *Science*	Spain	354	✔
5	Earliest hunting scene in prehistoric art. *Nature*	Indonesia	310	
**Not Paleolithic Archaeology**				
**Rank in category (rank total)**	**Citation**	**Location**	**Score**	
1 (7)	Archaeobotanical evidence reveals the origins of bread 14,400 years ago in northeastern Jordan. *PNAS*	Jordan	301	✔
2 (8)	Revealing a 5000-y-old beer recipe in China. *PNAS*	China	279	
3 (10)	Female hunters in the early Americas. *Science Advances*	Peru	260	✔
4 (12)	Ancient genomes revisit the ancestry of domestic and Przewalski’s horses. *Science*	Kazakhstan	255	✔
5 (15)	Early Neolithic wine of Georgia in the South Caucasus. *PNAS*	Georgia	247	✔
**China**				
1 (8)	Revealing a 5000-y-old beer recipe in China. *PNAS*	China	279	
2 (13)	A late Middle Pleistocene Denisovan mandible from the Tibetan Plateau. *Nature*	China	249	✔
3 (19)	The origins of cannabis smoking: Chemical residue evidence from the first millennium BCE in the Pamirs. *Science Advances*	China	186	✔
4 (31)	The earliest unequivocally modern humans in southern China. *Nature*	China	155	
5 (56)	Late Pleistocene archaic human crania from Xuchang, China. *Science*	China	95	

**Fig. 1.**
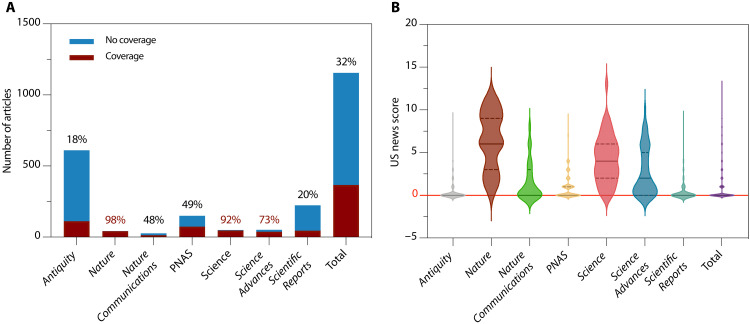
Archaeology research papers and US news coverage by journal. (**A**) Number of archaeology papers published in seven journals and percentage with US news coverage (US news score > 0). (**B**) For archaeology research in seven journals, violin plots show the distribution of US news scores, a sum of news mentions in 15 US sources. Center solid lines represent the median and dashed lines represent the first and third quartiles.

Of the total papers, 32% (367 of 1155) received attention from one or more of the 15 US media outlets considered in this study (Fig. 1 and Table 1). In other words, the majority (68%) of archaeology research papers received no attention from these outlets or a value of 0 by our US news score. Percent coverage (research papers with US news score > 0/total research papers) varied considerably by journal, showing a positive correlation with the journals’ 5-year impact factors (*R*^2^ = 0.82) and a negative correlation with the number of archaeology papers published in each journal (*R*^2^ = 0.52). The coverage rates ranged from 98% (41 of 42) for *Nature* to 20% (45 of 224) for *Scientific Reports* and 18% (112 of 611) for *Antiquity*. Relative to the specialist journal *Antiquity*, controlling for potential confounders, the likelihoods of coverage were significantly higher for all general science journals except *Scientific Reports* and *Nature Communications*.

### Influence of press release, paleolithic archaeology, and location

**Fig. 2.**
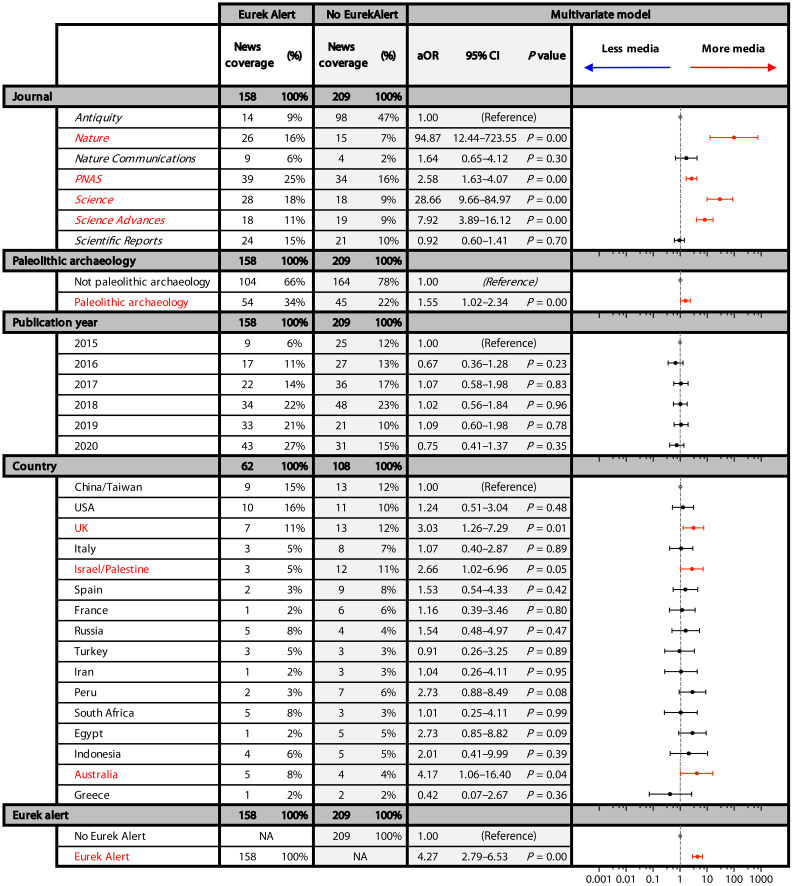
Adjusted odds ratios for US news coverage at country scale. Results of mixed-effects logistic regression models to calculate adjusted odds ratios (aOR) for US news coverage by each covariable. Adjusted odds ratios were relative to *Antiquity* for journal, articles that did not concern paleolithic archaeology, 2015 for year, China/Taiwan for country, and no inclusion in EurekAlert!. The 95% confidence intervals of statistically significant aOR are colored. The EurekAlert!/No EurekAlert! columns report the number and percentage of articles with US news coverage (US news scores > 0) for a respective covariable, with and without inclusion in this press release service.

In what follows, confidence intervals for odds ratios are reported at 95%. Articles with press releases on EurekAlert! had a percent coverage of 71% (158 of 224) compared to the 22% (209 of 931) coverage for articles not included in this press release service. In the logistic regression models, having a press release on EurekAlert! increased coverage likelihood 4.09 to 4.31 times, depending on whether analyzing data by country, subcontinent, or continent (Fig. 2 and figs. S3 and S4). Paleolithic archaeology articles showed greater percent coverage than articles that did not concern this specialization: 48% (99 of 207) compared to 28% (268 of 948), respectively. Paleolithic archaeology articles were between 1.53 and 1.60 times more likely to receive coverage, depending on geographic level of analysis (Fig. 2 and figs. S3 and S4).

**Fig. 3.**
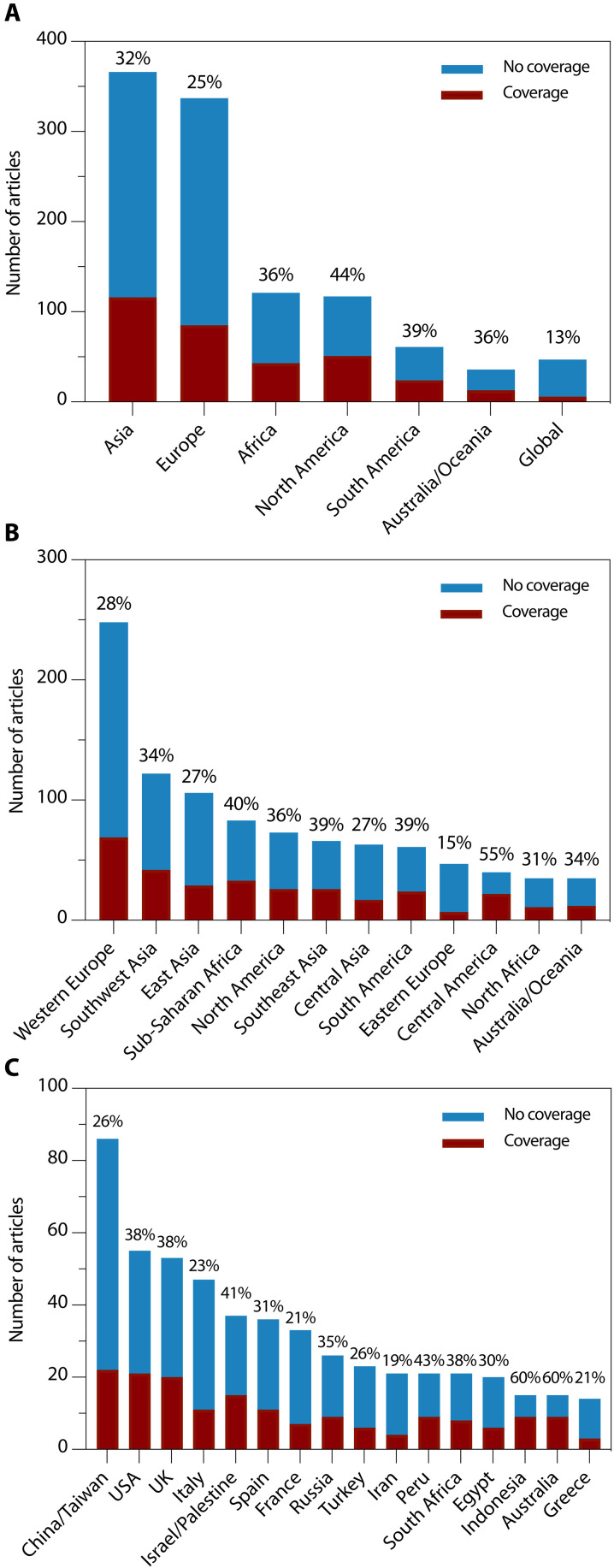
Archaeology research papers and US news coverage by region. The number of archaeology papers published and percentage with US news coverage (US news score < 0) by (**A**) continent, (**B**) subcontinent, and (**C**) country.

At the continent scale, Asia had the highest number of research papers and a US news percent coverage of 32% (116 of 366) (Fig. 3A). Excluding paleolithic archaeology papers, Asia’s coverage fell to 25% (72 of 283). North America received the highest percent coverage of 44% (51 of 117), a percentage that remained the same when paleolithic archaeology studies were excluded (48 of 109). Based on the mixed-effects logistic regression models, no continents differed statistically in odds of coverage from Asia (fig. S3). However, articles focused on North America were 2.0 times (1.09 to 3.60) more likely to have EurekAlert! press releases than articles focused on Asia (data S4).

At the subcontinent scale (figs. S4 and S5), Western Europe had the highest number of research papers and a percent coverage of 28% (69 of 248) or excluding paleolithic archaeology, 25% (52 of 205) (Fig. 3B). Central America showed the highest percent coverage of 55% (22 of 40) but relatively few research papers. Relative to East Asia, Central America was 4.0 times (1.66 to 9.75) more likely to receive coverage. In analysis with only the general science journals, Central America was also more likely to receive coverage than East Asia [9.2 times (1.97 to 42.47)] (data S5). The likelihood of a press release in EurekAlert! was approximately three times for Central America [3.6 times (1.21 to 10.76)], Central Asia [3.4 times (1.24 to 9.32)], North America [3.5 times (1.47 to 8.22)], and sub-Saharan Africa [2.8 times (1.11 to 6.90)] compared to East Asia (data S4).

At the country scale, we focused on the 16 countries with more than 14 research papers (Fig. 3C). Among these, China/Taiwan, United States, United Kingdom, Italy, Israel/Palestine, Spain, France, and Russia collectively accounted for 71% of the single country articles published across the 6 years. China/Taiwan produced the most research papers with a percent coverage of 26% (22 of 86), which fell to 18% (13 of 72) when paleolithic archaeology articles were excluded. The United States had the second most research papers, but notably higher media attention, with a percent coverage of 38% (21 of 55) for all papers and 40% (19 of 48) without paleolithic archaeology papers. Indonesia [60% (9 of 15)] and Australia [60% (9 of 15)] had the highest precent coverage, but relatively few total research papers. Considering countries with at least 20 research papers, the highest percent coverages belonged to Peru [43% (9 of 21)] and Israel/Palestine [41% (15 of 37)].

**Fig. 4.**
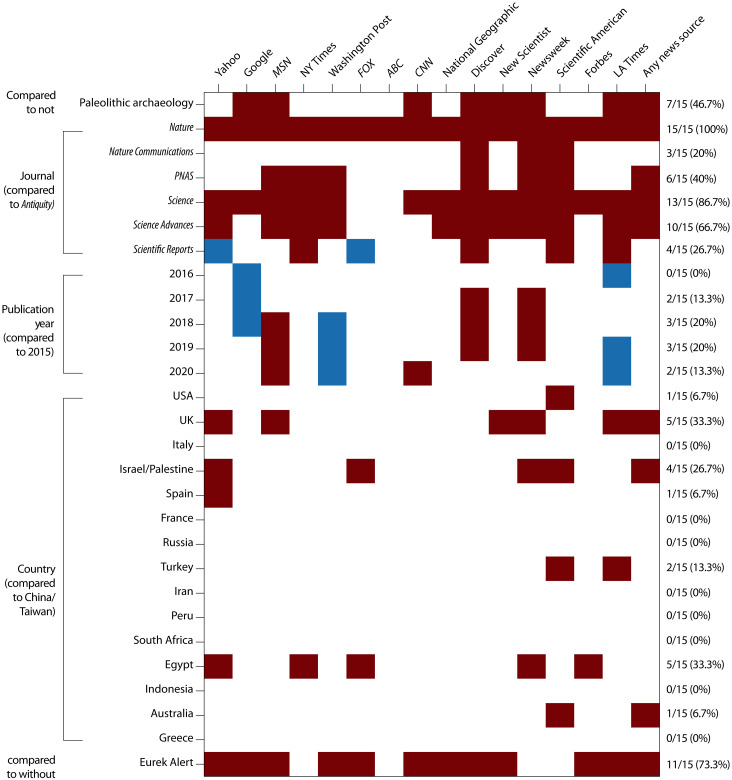
Coverage of archaeology research papers across 15 US news sources. Colored cells indicate significant results for comparisons between a row variable and the reference for its category, which were non-paleolithic archaeology, *Antiquity*, 2015, China/Taiwan, and no EurekAlert!. Red indicates that the reference is significantly less likely to receive coverage than the row variable, and blue indicates that the reference is significantly more likely to receive coverage than the row variable.

We ran the model using each of these 16 countries as the reference to see whether the remaining countries were more or less likely to receive coverage than the reference (fig. S6). No country was significantly less likely to receive US news coverage than China/Taiwan. The odds ratio of overall coverage was significantly higher than China/Taiwan for Israel/Palestine [2.7 times (1.02 to 6.96)], United Kingdom [3.0 times (1.26 to 7.29)], and Australia [4.2 times (1.06 to 16.40)] (Fig. 2). Analyzing each news source independently, Egypt, Spain, Turkey, and United States (in addition to Israel/Palestine, United Kingdom, and Australia) also emerged as countries statistically more likely to be reported by some outlets compared to China/Taiwan (Fig. 4 and data S4). In analysis of only the specialist journal *Antiquity*, the odds ratios for coverage were significantly higher than China/Taiwan for Egypt [5.7 times (1.17 to 28.00)], United Kingdom [3.9 times (1.08 to 14.02)], and Peru [6.4 times (1.14 to 35.39)] (data S5). EurekAlert! was more likely to contain a press release for articles about archaeology in Turkey [7.5 times (1.67 to 33.75), United Kingdom [7.4 times (2.23 to 24.71)], and United States [4.2 times (1.54 to 11.61)], relative to China/Taiwan (data S4).

## DISCUSSION

### Increasing research publication

The view of academic and popular publishing captured here is a 6-year snapshot of a dynamic media environment. Accordingly, the frequency of archaeology research appearing in various outlets may have changed since our study period. For example, anecdotally, we observe a recent uptick in archaeology research reported in *The Miami Herald*, and much of this seems to cover Asia and Central/South America.

That said, our data from 2015 to 2020 reveal patterns of global research production and US domestic news coverage in archaeology. Across one specialist and six general science journals, the total number of archaeology papers more than doubled across this span (fig. S1 and table S1). All seven journals increased their yearly total of archaeology papers. The largest proportionate increases were in *Scientific Reports* (from 11 to 70 articles) and *Science Advances* (2 to 14), which are also the only two journals to experience continuous growth in archaeology articles during this period.

### News factors driving archaeology coverage

In our dataset, 32% of the archaeology research papers received media coverage in at least one analyzed US news outlet and 51.7% were mentioned in at least one global news outlet included in Almetric’s database. This percentage likely exceeds the rate for media coverage of research across all fields, although comparisons with identical methodology are unavailable. One study found that the magazine *Time* and TV program NBC News covered fewer than 0.34% of all journal articles and fewer than 0.005% of papers from fields outside of health/medicine between 1990 and 2001 (*46*). In a more extensive study, Yu *et al.* (*11*) analyzed more than 670,000 journal articles and other scholarly publications in Altmetric’s database as of 2018 and found that only 6.3% received media attention. Scholarly outputs in their analysis received an average of 3.5 global news mentions compared to an average of 18.4 global news mentions for our dataset of archaeology articles from seven journals.

In Yu and colleagues’ analysis, health and medicine research made up roughly 53% of the scholarly outputs with media coverage, while history and archaeology only amounted to 0.97%. Thus, it appears that archaeology research constitutes a small proportion of the science news ecosystem, but within archaeology, a relatively high proportion of research gets coverage, and the top-reported research gets substantial coverage (table S2).

It should be expected that only a fraction of scholarly publications generates media attention. Many scientific articles have little immediate public relevance or newsworthiness, but high value to scholars and practitioners, such as methods development, review articles, and field reports in archaeology. In our study, the research papers reported by global (Table 2 and table S2) or US news outlets (Table 3 and table S3) had attributes (or news factors) thought to make events newsworthy for general news and science news, specifically (text S1).

**Table 3.** Comparison of research papers receiving top US news scores. Scores are sum of mentions for research paper in 15 major US news outlets or aggregators.

US news score				
All articles				
**Rank**	**Citation**	**Location**	**Score**	**Eurek Alert!**
1	The earliest modern humans outside Africa. *Science*	Israel/Palestine	13	✔
2	A 130,000-year-old archeological site in southern California, USA. *Nature*	United States	12	
3	The age of the hominin fossils from Jebel Irhoud, Morocco, and the origins of the Middle Stone Age. *Nature*	Morocco	11	✔
3	Human origins in a southern African palaeo-wetland and first migrations. *Nature*	Botswana, South Africa	11	
3	Evidence of human occupation in Mexico around the Last Glacial Maximum. *Nature*	Mexico	11	✔
3	Human occupation of northern Australia by 65,000 years ago. *Nature*	Australia	11	✔
**Not Paleolithic Archaeology**				
**Rank in category (total rank)**	**Citation**	**Location**	**Score**	**Eurek Alert!**
1 (7)	The origins of cannabis smoking: Chemical residue evidence from the first millennium BCE in the Pamirs. *Science Advances*	China	10	✔
2 (12)	Early Neolithic wine of Georgia in the South Caucasus. *PNAS*	Georgia	9	✔
3 (12)	Native American gene flow into Polynesia predating Easter Island settlement. *Nature*	Rapa Nui (Easter Island, Chile)	9	✔
4 (12)	Megalith quarries for Stonehenge’s Bluestones. *Antiquity*	United Kingdom	9	
**China**				
**Rank in category (total rank)**	**Citation**	**Location**	**Score**	**Eurek Alert!**
1 (7)	A late Middle Pleistocene Denisovan mandible from the Tibetan Plateau. *Nature*	China	10	✔
1 (7)	The origins of cannabis smoking: Chemical residue evidence from the first millennium BCE in the Pamirs. *Science Advances*	China	10	✔
3 (12)	Late Pleistocene archaic human crania from Xuchang, China. *Science*	China	9	
4 (23)	The earliest unequivocally modern humans in southern China. *Nature*	China	8	
5 (37)	Revealing a 5000-year-old beer recipe in China. *PNAS*	China	7	

Interviews with editors and content analysis indicates that newsrooms often base their assessment of the news factor of scientific relevance on journal prominence or the presence of a press release (*17*). This finding is corroborated by our data: The top five articles by global and US news scores were published in *Nature* or *Science*, the journals with the highest impact factors. More than 90% of the archaeology papers published in *Nature* or *Science* were covered by at least one of the 15 US media outlets considered in this study. Relative to *Antiquity*, while controlling for other potential confounding factors, the likelihood of coverage was between 95 and 124 times more likely for *Nature* papers and 29 and 32 times more likely for *Science* papers (Fig. 2 and figs. S3 and S4).

In considering the effect of press releases in signaling scientific value, we focus on the relationship between US news score and inclusion on EurekAlert!, a US-based news release service. Nine of the 11 of the articles with the highest US news scores had press releases on EurekAlert! (table S3). Inclusion in the service did not guarantee media coverage, but the percent coverage was substantially higher for articles with the press releases: 70% (158 of 224) of articles with EurekAlert! press releases received US media coverage compared to a coverage rate of 22% (209 of 931) for articles without the press releases. Articles with a EurekAlert! press release were about four times more likely to receive coverage from at least one analyzed US news source. The positive effect of press release on media coverage is expected and has been demonstrated in previous studies of general science and medical research dissemination (*47*, *48*). Press releases alert media workers to the existence of new research, explain its significance in non-specialist language, and have been considered an indicator of scientific relevance by editors (*17*). Some media outlets repeat exaggerations or caveats of press releases (*49*) or directly republish their content. Furthermore, many articles/press releases included in EurekAlert! are distributed under embargo to journalists before the article publication. The embargo system allows journalists to prepare stories ahead of time but requires news outlets to delay publication until the research article appears in its respective journal. We assume that embargoed articles with press releases are more likely to receive coverage than non-embargoed articles with press releases (*50*). A limitation of this study is that we did not distinguish between EurekAlert!‘s embargoed and non-embargoed articles.

In addition, we found that regions were not equally represented on EurekAlert! (data S4). Relative to articles about archaeology in Asia, articles focused on the North American continent were about two times more likely to appear on the service. At the subcontinent level, the likelihood for inclusion in EurekAlert! was significant compared to East Asia for Central America, Central Asia, North America (as subcontinent including United States and Canada), and sub-Saharan Africa. At the country level, the likelihood for inclusion in EurekAlert! was significant compared to China/Taiwan for Turkey [7.52 times (1.67 to 33.75)], United Kingdom [7.42 times (2.23 to 24.71)], and United States [4.23 times (1.54 to 11.61).

Focus on paleolithic archaeology also seemed to drive news coverage, perhaps reflecting the news factors (text S1) of novelty—these studies often report the oldest known example of a human biological or cultural trait—or proximity, in the sense that human origins research reconstructs the shared history of all living people. In addition, paleolithic archaeology studies often present unexpected fossil findings, which aligns with science journalism’s prioritization of superlative finds and breakthroughs over incremental progress (*20*). Nearly all articles with the most coverage in global (7 of 10) (table S2) or US news (10 of 11) (table S3) concerned paleolithic archaeology. Considering the full dataset, paleolithic archaeology articles were covered at rate of 48% (99 of 207) and with an odds ratio about 1.5 times that of articles that did not concern this topic, which had a percent coverage of just 28% (268 of 948). To evaluate our hypothesis that novelty or proximity explain paleolithic archaeology’s popularity, future research could follow the methodology of Badenschier and Wormer (*17*), which comprised interviews with news editors about the reasoning behind their decisions. A study could also explore whether this pattern extends to other areas such as museum exhibits and university courses.

Excluding paleolithic archaeology, remaining articles that received considerable global or US coverage (Tables 2 and 3, and tables S2 and S3) reported early bread, beer, wine, or cannabis—likely all considered newsworthy for novelty (*51*–*54*). Also included were articles about the prevalence of female hunters in the ancient Andes and Indigenous American gene flow into Polynesia, which may have been newsworthy for unexpectedness because they challenge traditional thinking or scholarship in archaeology (*55*, *56*).

### Perceived proximity and geographic biases

Proximity between a story and newsroom has long been considered a primary news factor driving coverage (text S1). As mass media has evolved in the digital age, reaching more disparate or diverse audiences, media scholars have conceptualized proximity as a multidimensional construct comprising the distance between story and audience, measured by physical, geographic units, and psychological factors based on cultural, political, or economic ties (*15*, *16*). The latter concept of proximity has been applied to explain geographic disparities in coverage or reader interest for natural disasters (*15*) and other international events in print and television news (*57*–*59*). To quantify the strength of geopolitical ties between the United States and a foreign nation, analyses have used metrics such as trade flow or US military presence, controlling for measures of global “prominence” such as gross domestic product and population size (*60*).

For archeological research, we hypothesized that distance between story and audience will compound geographic proximity and perceived cultural proximity of past people—in other words, stories about the places people consider “home” and the audience’s real or imagined ancestors. A priori, we expected US media outlets to disproportionately cover research considered proximate to their US readers. Considering the geographic component of proximity, this interest would focus on the United States despite (or perhaps because of) the history of white settlers violently colonizing Native Americans and occupying their lands in forming the United States. We assume many non-Native American readers consider the United States their home and might feel connected to research about archaeology in the US, including its predominately precolonial past. The percent coverage of articles for North America [44% (51 of 117)] was indeed the highest among continents. The United States was also among the countries with highest percent coverage [38% (21 of 55)], particularly when removing paleolithic archaeology articles from those considered [40% (19 of 48)]. However, in the logistic regression models, North America and the US were not significantly more (or less) likely to receive coverage than any other regions (data S6 and fig. S6). The only geographically proximate region to produce significant results was Central America, which was roughly four times more likely to receive coverage than East Asia (fig. S4). Therefore, we found limited support for the geographic component of proximity being influential.

Perceived cultural proximity, however, could explain geographic disparities that did emerge in the analysis. When controlling for journal, focus on paleolithic archaeology, and inclusion on EurekAlert! within the logistic regression models, no geographic regions were significantly less likely to receive news coverage than Asia or East Asia. At the country level, the odds ratio of overall coverage was significantly higher for Israel/Palestine, United Kingdom, and Australia (Fig. 2). Considering individual news outlets, countries significantly more likely to receive coverage than China/Taiwan included Australia (for *Scientific American*), Egypt (*Yahoo*, *The New York Times*, *FOX*, *Newsweek*, and *Forbes*), Israel/Palestine (*Yahoo*, *FOX*, *Newsweek*, and *Scientific American*), Spain (*Yahoo*), Turkey (*Scientific American* and *Los Angeles Times*), United Kingdom (*Yahoo*, *MSN*, *New Scientist*, *Newsweek*, and *Los Angeles Times*), and United States (*Scientific American*) (Fig. 4 and data S4). This is despite our finding that the total and non-paleolithic archaeology count for research papers was highest for Asia and China/Taiwan and third highest for East Asia for the respective geographic levels.

It is possible that this underrepresentation could be due to saturation: Because so many research papers cover archaeology in China/Taiwan and Asia, news outlets are rejecting some as potential stories to ensure a diverse composition of their publications. Composition, the mix of topics in an issue or broadcast, is considered a classic and science news factor (*13*, *17*). Although the proportion of articles covered for China/Taiwan was lower, the count of 22 total reported papers was higher than all other countries, including the United States with 21 and United Kingdom with 20 reported papers.

However, other aspects of the analysis and previous scholarship suggest that composition alone does not explain the observed regional disparities. Of the Chinese articles that garnered high US news scores (Table 3), the top five can be explained by news factors of proximity and novelty: three covered paleolithic archaeology (*61*–*63*), which concerns the shared past of our species; one reported the origins of cannabis smoking (*53*); and another documented a 5000-year-old beer recipe (*54*). Only 13 of the 72 articles (18%) about China/Taiwan that did not report paleolithic finds received media coverage compared to 9 of the 14 articles (64%) that did cover paleolithic archaeology.

Perceived cultural proximity likely contributes to archaeology research’s news value. However, any measures of cultural distance are problematic in the context of US media as the audience descends from diverse ancestries. Using total population as a rough proxy for news audience, 2020 US Census data suggest a public that identifies alone or in combination as 71% white, 15.1% some other race, 14.2% Black or African American, 7.2% Asian, 2.9% American Indian and Alaska Native, and 0.5% Native Hawaiian and other Pacific Islander [The census reports data for individuals who only identified as one racial category (“alone”), multiple racial categories (“in combination”), and a summed value of alone or in combination. Thus, these values, capturing alone or in combination, sum to more than 100%. The census also asks Hispanic identity as a separate question from racial identification. See www.census.gov/library/stories/2021/08/improved-race-ethnicity-measures-reveal-united-states-population-much-more-multiracial.html]. While these socially constructed racial categories are not based on genetics or biology (*64*), in the United States, they carry implicit assumptions about ancestral ties. Moreover, newsrooms are considerably less diverse than the US population with more than 75% of employees and 90% of supervisors identifying as white in surveys conducted during the years our dataset spans (*65*, *66*). Within science journalism, full member respondents to a 2021 survey of National Association of Science Writers (NASW) identified as 81% white, 4.7% multiple categories, 3.3% Asian, 1.6% Hispanic or Latino, and 0.9% Black or African American (*67*). Considering religion as a component of cultural ties, in 2020, the US population identified as 64% Christian, 30% no religion, and 6% other religious groups, according to Pew Research Center data (*68*). Given disparities in racial and religious identification of the US population and racial identification among newsroom staff/leadership, US media coverage of archaeology research may overrepresent past people and places considered relevant to white and Judeo-Christian heritage.

In addition, anti-Asian and anti-Chinese sentiments have long persisted in the United States (*69*). Discriminatory laws in the 19th century, such as the Chinese Exclusion Act of 1882, limited immigration and naturalization of people from China. During the timeframe of our dataset, from 2015 to 2020, a survey found between 47 and 73% of US adults held an unfavorable view of China, which peaked with the COVID-19 pandemic. As of 2024, the percentage of US adults viewing China unfavorably had grown to 81% (*70*).

Compounding effects of anti-Chinese and pro-white/Christian biases could explain the finding that the likelihoods of coverage for the United Kingdom, Israel/Palestine, and Australia were between 2.7 and 4.2 times compared to China/Taiwan, when controlling for other potential confounding factors. Moreover, for individual news sources, countries statistically more likely to receive coverage than China/Taiwan included Australia, Egypt, Israel/Palestine, Spain, Turkey, United Kingdom, and United States. Our anecdotal observation that research about Egyptian archaeology is overreported relative to archaeology of China (*36*) was not supported by aggregated analysis of 15 US news sources. However, the premise was supported for several particular, prominent news outlets: Compared to archaeology of China/Taiwan, stories about Egypt were more likely to appear in *The New York Times* [10.59 times (1.56 to 72.00)], *Yahoo* [4.30 (1.02 to 18.13)], *FOX* [4.23 (1.03 to 17.48)], *Newsweek* [11.38 (1.94 to 66.82)], and *Forbes* [43.67 (4.91 to 388.23)] (Fig. 4 and data S4).

A country-by-country pairwise comparison supports the proposed biases (fig. S6). There were no significant results for other “non-Western” countries, including Russia, South Africa, and Indonesia. Only three countries were statistically less likely to receive coverage than others: Greece compared to the United Kingdom and Australia; Italy compared to the United Kingdom; and China/Taiwan compared to the United Kingdom, Israel/Palestine, and Australia. Of these comparisons, Greece and Italy were less likely to receive coverage than countries that could be considered part of the same white European culture: the United Kingdom and Australia, a former colony of the British Empire that US media might associate with white European culture despite its original and continuous Aboriginal population. Moreover, in these intracultural comparisons, the English-speaking countries received higher attention in US media. In contrast, China/Taiwan’s lower odds ratios appeared in intercultural comparisons between “Eastern” culture and countries important in “Western” heritage or imperialism. However, as a counterpoint to our argument, we would have expected Italy and Greece to be significantly more likely to receive coverage than non-Western countries, considering narratives of Ancient Greece and Rome as progenitors of Western civilization (*71*). The results for Greece and Italy in our models merit further investigation.

Considering all the factors that contribute to research’s news value, this geographic influence may be subtle in practice. As evidence, when articles from the specialist journal *Antiquity* were removed from the dataset, no country was more or less likely to receive coverage than China/Taiwan (data S5). In analysis of only the *Antiquity* articles, which constituted more than half the total dataset (611 of 1155), Egypt, United Kingdom, and Peru were more likely to receive coverage than China/Taiwan. We hypothesize that the appearance of archaeology research in general science journals signals to editors that the work holds scientific relevance amounting to newsworthiness. This news value may dampen considerations of cultural or geographic proximity indicated by the location of the research. However, we contend that the full dataset, including *Antiquity*, more closely resembles the pool of potential stories from which editors make selections. Although only 18% of *Antiquity* articles received coverage that still amounted to 112 total articles, roughly one-third of the articles with US media coverage in our dataset.

Given that *Antiquity* represents nearly half the journal articles, its publication rates for different regions disproportionately influence the geographic makeup of our raw dataset. *Antiquity* published nearly as many articles about Asia (197) as Europe (212) and China/Taiwan (*34*) as the United Kingdom (*48*), but fewer articles at the continent scale for Africa (*68*), North America (*50*), South America (*28*), and Oceania/Australia (*11*). Notably, regions at the center of our discussion (Asia versus Europe and China/Taiwan versus United Kingdom) had similar article counts. Moreover, the mixed-effects logistic regression models account for sample size within each category.

### Potential consequences of geographic biases

Our analysis suggests limited media bias toward archaeology research geographically close to US audiences, with only Central America appearing to have significantly higher odds of coverage in a comparison of subcontinents (data S6). However, our analysis is consistent with the hypothesis that US media outlets conceive cultural proximity to mean stories about human origins—humanity’s shared ancestors—and regions considered important in white or Judeo-Christian history. There is a statistically significant underrepresentation of archaeology from China/Taiwan in aggregated and individual US news sources, which may reflect anti-Chinese sentiments, pro-white/Christian sentiments, or both.

This finding and its potential consequences build on decades of scholarship on the sociopolitics of archaeology, defined as “how archaeology serves the goals of the larger society which supports it, while it also mirrors the norms, prejudices, politics and economic structure of that society” [(*72*), p. 1]. Blakey (*73*) concluded that North Americans prioritize discussions of finds in the Middle East over finds in North Africa in ways that reflect cultural or economic priorities. This conclusion echoes the work of Trigger (*74*), who has tied the colonialist history of American archaeology to a focus on “Western civilization” and nationalist sense of identity as a white nation.

Work in social psychology has demonstrated related implicit biases, such as a finding that adults at a US university were more likely to associate Europeans (such as Hugh Grant) than Asian Americans (such as Connie Chung) with the United States (*75*, *76*). This implied “otherness” of non-white members of society and non-European histories relates to ways in which non-white voices, perspectives, and scholarship are underrepresented in knowledge production. Recent studies of gender and occupational affiliation in North American archaeology (*77*–*80*) have likewise noted imbalances related to these status characteristics in academic publishing and public-facing magazines. An early inquiry into this topic (*81*) showed that 20th century *National Geographic* focused proportionally little attention on the archaeology of entire countries and regions—India, China, Southeast Asia, Africa, and Australia—with preponderant coverage given to investigations in the Middle East and Europe.

Based on our data and previous studies, the rates with which US popular science reports archaeology about certain regions does not correspond with the rates of scientific publication about those regions. We do not suggest that the US mass media originated the observed regional affinities but rather is among the numerous sectors that perpetuate them. An emphasis on white and Judeo-Christian histories has long permeated US popular culture, reflected by these phenomena as Egyptomania (*82*, *83*) and high tourism to the “holy lands” and Europe. Furthermore, studies of US primary and secondary history curricula have demonstrated an emphasis on white histories over those of other racial/identity groups [e.g., (*84*–*86*)]. Whether in classroom lessons or popular press, these biases can reinforce notions of cultural affiliation that exclude and diminish certain identity groups from what is understood as the relevant pasts of people living in what is now the United States.

### Gates and gatekeepers in research dissemination

The findings of this study also highlight ways for different actors to diversify media coverage of archaeology. When considering how academic research reaches publics, we will focus on one common pathway, recognizing other avenues for science outreach exist: Scholars submit research manuscripts to academic journals. The peer review process and journal editors determine which submitted manuscripts are published as research papers. Some of these research papers have associated press releases, often created through collaboration between study authors and press offices at research institutions or journals. One major aggregator of science news releases in the United States is AAAS’ EurekAlert!, which hosts press releases for institutions that have paid a submission fee. Articles without press releases may still be noticed by writers or journalists, particularly freelance writers, who send pitches suggesting research as a story to news outlets. Either through press release or pitch process, mass media editors decide which research papers are newsworthy for their respective audiences. Assigned to a writer, this research is reported as “pop-science” stories.

Within this pathway, we highlight numerous actors: research paper authors, academic journal editors and peer reviewers, communications workers involved in press release creation and distribution, science writers/journalists, and news outlet editors. Expanding the media studies concept of gatekeeping, these actors stand at various gates or decision points, which determine whether a research paper will be published, publicized, and reported by mass media (*16*, *87*).

Acknowledging a balance of risks and rewards to media coverage, which will vary with a scholar’s positionality, we identify “gates” researchers can open to increase the likelihood their work receives media coverage. By our analysis, journal was one of the most substantial drivers of media attention, with more than 90% of archaeology papers published in *Science* and *Nature* reported by at least one US news source. As acceptance rate for these publications is low, archeologists more often publish in other journals we analyzed, and our data indicate that articles in these lower-impact journals did not receive equal media coverage. Our multivariant analysis shows that relative to the specialist journal *Antiquity*, articles were statistically more likely to receive US media coverage when published in *PNAS* and *Science Advances*. However, articles in *Scientific Reports* and *Nature Communications—*journals that had impact factors of 4.9 and 17, respectively—were *not* statistically more likely to receive coverage compared to *Antiquity*, with an impact factor of 2. Thus, if archeologists want to tailor their manuscripts to an audience of mostly fellow archeologists, *Antiquity* is an option that did not statistically reduce media attention compared to *Scientific Reports* and *Nature Communications*, both broad open-access journals from the Nature Portfolio publishing group. Alternatively, researchers can publish in their preferred journals and then write about their work directly for the public in media outlets that have scholars/scientists as authors, including *Scientific American, The Conversation*, and SAPIENS. We note that one author, B.A., is an editor at SAPIENS.

Moving to the peer-review gate, our data do not track potential disparities in manuscript submission and acceptance. Numerous previous studies have identified a geographic bias in academic and scientific publishing, which preferences journals or authors from the Global North or high-income countries relative to the Global South or low- to middle-income countries (*88*–*93*). All articles we analyzed were published in Global North—UK- or US-based—journals, and we did not consider the institutional location of authors, but rather the location of past archeological peoples. In addition, our study does not contain data about acceptance rates of manuscripts about different regions or topics. Across seven journals and 6 years, the most archaeology articles concerned materials from China/Taiwan, followed by the United States and United Kingdom. Countries with archaeology reported in more than 20 research articles also included (in descending order): Italy, Israel/Palestine, Spain, France, Russia, Turkey, Iran, Peru, South Africa, and Egypt. From the available data, economic status or classification as Global North/South did not appear to influence the number of research papers published about archaeology in a given country.

Press releases on EurekAlert! did emerge as a regionally biased gate in the research dissemination pathway. Articles about research in Turkey, United Kingdom, and United States were significantly more likely to have press releases included on EurekAlert! than articles about China/Taiwan. Furthermore, articles included on this service were about four times more likely to receive US media coverage. We note that this analysis focused on one press release service and 15 major US news sources. The effects of press releases disseminated by other means and for smaller news outlets remains unexplored. Still, the significant effect and apparent regional bias of press releases in our data leads to an obvious recommendation: Research paper authors who desire media coverage should work with press offices at publishing journals and/or their research institutions to create press releases shared by EurekAlert!. However, not all researchers work for/with institutions that have press offices or the funds to pay submission fees for EurekAlert! or similar platforms. The accessibility of press releases is another factor that contributes to institutional equalities in researcher productivity and visibility (*94*, *95*).

We also note that for just the journal *Antiquity*, 47% percentage of articles without EurekAlert! press releases received media coverage compared to just 9% with releases on this service. It is possible this pattern reflects another branch in the research dissemination pathway, by which journalists submit story ideas through a pitch process. To find exclusive story ideas, writers look beyond press releases, including attending conferences and following specialist journals. We do not have data to test this hypothesis but base it on published advice from journalists [e.g. (*96*–*98*)) and the training and experiences of one author (B.A.) who has worked as a freelance science writer. Based on this study’s results, journalists seeking exclusive or under-reported stories to pitch should consider topics, regions, and journals underrepresented in our dataset of media coverage and EurekAlert! inclusion.

Last, newsroom editors, as the final gate in our research pathway, can use these results to inform their assessment of a research article’s news value. Topics/regions/journals underrepresented in our coverage data potentially offer news and science news factors of exclusivity, unexpectedness, and astonishment (*13*, *17*). News organizations maintaining commitments to justice, equity, diversity, and inclusion should reflect on how they implicitly or explicitly define cultural proximity between their audiences and archeological contexts.

Media attention does not directly correlate with scientific value of research studies—nor should it, given the non-identical communication goals of scholarly and popular science publications. However, our study raises concerns by showing that some geographically distant regions are more likely to receive media coverage for archaeology research, when controlling for journal, press release inclusion, publication year, and paleo-subject matter. In an aggregated look at 15 US news sources, we found that coverage was more likely for archaeology of United Kingdom, Israel/Palestine, and Australia, relative to China/Taiwan. Analysis of each news source individually suggests greater odds of coverage in particular outlets of archaeology of Australia, Egypt, Israel/Palestine, Spain, Turkey, United Kingdom, and United States. Building on scholarship across media studies, the sociopolitics of archaeology, social psychology, and curriculum studies, we argue that these regional disparities reflect anti-Chinese biases and false notions that the histories relevant to people in the United States are from white, Judeo-Christian pasts—a viewpoint that is also integral to the ideology of white Christian nationalism. More regionally diverse media coverage of archaeology research—closer aligned with journal research outputs—could play a small role in equitable and accurate representation of the diverse ancestries that are culturally proximate to people in the United States.

## MATERIALS AND METHODS

### Data collection

Altmetric offers free access to university-affiliated researchers studying scientometric topics through the researcher data access program (www.altmetric.com/our-audience/researchers/research-access/). Using the company’s application portal, we requested and received complimentary researcher access for a limited time to Altmetric Explorer, a more comprehensive and searchable platform than the freely available Altmetric website. Within Altmetric’s database, articles are classified into subject areas by its sister company dimensions, which applies a machine learning algorithm to article abstracts to categorize them into fields of research (FoR), devised by the Australian and New Zealand Standard Research Classification. In September 2021, we collected our dataset with Altmetric Explorer’s advanced search: For the FoR, we specified archaeology and restricted it to “articles,” which should be peer-reviewed research studies. We narrowed this dataset to research papers published between 1 January 2015 and 31 December 2020 (the 6 years before the start of this study) in one specialist journal, *Antiquity*, and six general science journals: *Science*, *Nature*, *Science Advances*, *Nature Communications*, *Scientific Reports*, and *PNAS*. The selected general science journals include those that regularly publish archeological content with the highest impact factors and SCImago Journal Rankings (*43*). Among the archaeology journals with similar rankings, *Antiquity* engages in media outreach and offers wider methodological and geographical breadth. From this search, we downloaded a dataset of “research outputs” (the journal articles, data S2) and associated “mentions” (news stories, Tweets, blog posts, and other online mentions, data S3). We worked from this timestamped dataset so that the Altmetric data would not change over the course of our analysis.

The search produced 3111 research outputs, which we manually reviewed to ensure each article presented peer-reviewed research about archaeology. We defined archaeology research as analysis, interpretation, synthesis, experimental work, or methods development related to material culture, paleoenvironmental remains, or biomolecules, which produces knowledge about past humans. Most of the omitted outputs (1057 of the 1956 omitted) were older *Antiquity* studies published in print before 2015 that were first published online during our study span. For one example, see Steven Snape “Neb-Re and the heart of darkness: the latest findings from Zawiyet Umm el-Rakham (Egypt)” *Antiquity*, with a 2001 original print publication date and 02 January 2015 online publication date.

We also removed book reviews, editorials, and studies that did not present original archeological data or analysis. The latter category mostly comprised earth sciences papers that used a method also applied in archaeology such as carbon isotopes analysis. For example: Rowe *et al.* (2019) “Black carbon and other light-absorbing impurities in snow in the Chilean Andes” *Scientific Reports*; Ishibashi and Sakai 2019 “Dispersal of allergenic pollen from *Cryptomeria japonica* and *Chamaecyparis obtusa:* characteristic annual fluctuation patterns caused by intermittent phase synchronisations” *Scientific Reports*; Choudhury *et al.* (2019) “The role of surface air temperature over the east Asia on the early and late Indian Summer Monsoon Onset over Kerala” *Scientific Reports*.

For the resulting dataset of 1155 research papers, we recorded Altmetric attention score, news mentions, other online mentions such as Twitter, and whether a press release appeared on EurekAlert! (data S1). For global news mentions, we applied Altmetric’s “highlights only” filter, which narrows media to high-profile, international news sources. We collected the titles of these news articles but did not review their content. It is possible that some are republications of press releases or stories originally published in another outlet. These ostensibly replicate data would not alter our findings because editors are still deciding to disseminate the research in question to their publication’s audience. It is also possible that some analyzed news articles merely reference a journal article while reporting on another study or event. Altmetric news mentions do not distinguish these cases, which is a limitation of the data.

From Altmetric’s highlights only news mentions, we calculated the US news score as the sum of a research paper’s mentions in 15 major US media outlets or aggregators that cover science: *Yahoo*, *Google*, *MSN*, *The New York Times*, *The Washington Post*, *FOX*, *ABC*, *CNN*, *National Geographic*, *Discover*, *New Scientist*, *Newsweek*, *Scientific American*, *Forbes*, and *Los Angeles Times*. In choosing US media outlets, we selected those that reported at least 35 research papers in our dataset, in addition to *Scientific American*, *Los Angeles Times*, *ABC*, and *The Washington Post*. Some outlets that report archaeology news were excluded from our analysis because they were absent from the Altmetric output (e.g., *Smithsonian Magazine*) or only appeared in small numbers (e.g., 1 article from Associated Press, 4 articles from *Slate Magazine*, 9 articles from *TIME*, 16 from NBC News). We assume Altmetric’s counts for these outlets are unreliable, an observation that adds to research on the limitations of altmetrics (*38*, *42*). Other outlets such as BBC (British Broadcasting Corporation) News, *El País*, and *Daily Mail* appeared in high numbers but were excluded because they are not US media outlets, the focus of our study.

For each research paper, we manually coded additional variables: geographic location by country, subcontinent, and continent; temporal span (absolute and archeological period); and concerning the topics of paleolithic archaeology, ancient DNA, or environmental archaeology (data S1). To investigate geographic patterns, we parsed the dataset at three scales (continent, subcontinent, and country). In each case, we identified any relevant geographical regions considered in the article. We assigned countries to subcontinents by consensus, focusing on geographic location and archeologically relevant cultural groupings (fig. S5). We mostly disregarded geopolitical associations related to colonization and other modern events. For example, we included Greenland in the North America subcontinent because of its geographic location and archeological history and not its status as an autonomous territory of Denmark. For India, we assigned articles to Southwest Asia if the research concerned the Indus Valley Civilization; other archeological research in India was assigned to Southeast Asia. When articles related to more than one area (country, subcontinent, or continent), we coded each of the relevant geographical units, separated with commas in the respective data cell. For example, at the country scale, a study that features sites in both Portugal and Spain would be coded as “Portugal, Spain.” In our analyses, we only considered papers that concerned a single region at a given scale. Accordingly, the Portugal, Spain paper would be excluded from country-level analysis. At the higher scales of subcontinent (Western Europe) and continent (Europe), it would be included in our analyses. For this reason, the number of articles included in logistic regression models varies across levels of country, subcontinent, and continent.

### Statistical analysis

Using STATA/BE 17.0, we developed mixed-effects logistic regression models to calculate adjusted odds ratios for US news coverage by region. Mixed-effects logistic regression models are chosen for their ability to model binary outcomes from both fixed and random effects and assess the impacts of both categorical and continuous predictor variables. In the models, the dependent variable, US news coverage, was treated as a binary variable of coverage (US news score > 0) or no coverage (US news score = 0). For analysis, we controlled for potential confounding variables such as paleolithic archaeology, journal, publication year, geographic region (continent, subcontinent, and country), and EurekAlert! press release. Limiting the data to single region articles, models estimated the adjusted odds that an article from a given continent, subcontinent, or country would receive coverage relative to Asia, East Asia, and China/Taiwan, respectively. Adjusted odds ratios for journals were relative to *Antiquity*, and the publication years were relative to 2015. Calculated adjusted odds ratio > 1.0 are associated with higher likelihoods of coverage by at least one of 15 major US news sources and deemed statistically significant if the associated *P* value was ≤ 0.05 by the Wald test. The analysis was repeated for only the specialist journal (*Antiquity*) and the six general science journals (all excluding *Antiquity*) (data S5), as well as with North America and each analyzed country used as the reference (fig. S6 and data S6).

Similar analyses were conducted for each news source alone with the dependent variable as a binary coverage by each respective news source, controlling for paleolithic archaeology, journal, publication year, geographic region (country with respect to China/Taiwan), and EurekAlert! press release (data S4). We also generated models to estimates odds ratio for an article to have a EurekAlert! press release by region, controlling for paleolithic archaeology, journal, and publication year, with respect to Asia, East Asia, and China/Taiwan (data S4).

## Supplementary Materials

### The PDF file includes:

Supplementary Text S1 and S2 Figs. S1 to S6 Tables S1 to S3 Legends for data S1 to S6 References
