# Supplementary material for: Regional disparities in US media coverage of archaeology research
Source: Sci Adv. 2025 Jul 2;11(27):eadt5435. doi: 10.1126/sciadv.adt5435 (PMC12219491; doi:10.1126/sciadv.adt5435)
Supplement: Supplementary file 1 — Supplementary Text S1 and S2 Figs. S1 to S6 Tables S1 to S3 Legends for data S1 to S6 References [file sciadv.adt5435_sm.pdf]

Science Advances
AAAS

Supplementary Materials for

Regional disparities in US media coverage of archaeology research

Bridget Alex *et al.*

Corresponding author: Bridget Alex, balex@fas.harvard.edu; Rowan Flad, rflad@fas.harvard.edu

Sci. Adv. **11**, eadt5435 (2025)
DOI: 10.1126/sciadv.adt5435

The PDF file includes:

Supplementary Text S1 and S2
Figs. S1 to S6
Tables S1 to S3
Legends for data S1 to S6
References

Other Supplementary Material for this manuscript includes the following:

Data S1 to S6

Supplementary Text

text S1. Determining Newsworthiness

Media scholars have formulated models to determine components that make an event newsworthy, or worthy of media coverage. Most identify a list of news factors that sum to an event's news value, which is assessed by editors at a given outlet (12). Such "laundry list" of indicators (14) often include factors such as timeliness, proximity, importance/impact, human interest, conflict, sensationalism, novelty, and prominence of people or places involved. For a simple summary see "The Components of 'Newsworthiness" by the Purdue Online Writing Lab (https://owl.purdue.edu/owl/subject_specific_writing/journalism_and_journalistic_writing/components_of_newsworthiness.html).

Over the decades, proposed news factors have been evaluated and updated through quantitative and qualitative methods, including content analysis and structured interviews, across different media genres and forms. For example, Harcup and O'Neill (13) proposed the following updated set of indicators that, in combination, seem to explain the content of 711 stories on news pages across 10 newspapers (descriptions have been shortened).

- **Exclusivity:** generated by or available first to the news organization
- **Bad news:** particularly negative overtones such as death, injury, loss
- **Conflict:** including controversies, arguments, splits, strikes, fights, insurrections, war
- **Surprise:** an element of surprise, contrast, and/or the unusual
- **Audio-visuals:** such as arresting photographs, video, audio, infographics
- **Shareability:** likely to generate sharing and comments via social media
- **Entertainment:** soft stories about sex, showbusiness, sport, lighter human interest, animals, or offering opportunities for humor, witty headlines, lists
- **Drama:** unfolding escapes, accidents, searches, rescues, battles, court cases, etc.
- **Follow-up:** subjects already in the news
- **Power elite:** concerning powerful individuals, organizations, institutions
- **Relevance:** about groups or nations perceived to be influential with or culturally/historically familiar to audience.
- **Magnitude:** large numbers of people involved, large potential impact, extremes
- **Celebrity:** people who are already famous
- **Good news:** particularly positive overtones such as recoveries, breakthroughs, wins, celebrations
- **News organization's agenda:** set or fit the news organization's agenda, whether ideological, commercial, or as part of specific campaign.

Focusing on science journalism, analysis by Badenschier and Wormer (17) identified 14 news factors with the highest impact on story selection (definitions originally modified to be applicable to science coverage and then shortened here). They note that scientific relevance was signaled by publication of a scientific paper in a journal and the reputation of that journal.

- **Astonishment:** an event causes amazed reactions ("Aah!")
- **Composition:** mix of topics within a distinct science page and whole issue
- **Controversy:** differences in opinions
- **Economic relevance:** importance of an event for the economy
- **Graphical material:** event becomes news because pictures or figures available
- **Intention:** type of science communication (e.g. investigative story or explainer)
- **Personalization:** persons important to the reported circumstances
- **Political relevance:** importance of event for politics or legislation
- **Range:** number of affected or participating people
- **Reference to elite persons:** political, economic, cultural, or scientific power of a person, group, or institution
- **Relevance to recipients/society:** importance of an event for article recipient or society
- **Scientific relevance:** importance of an event for scientific progress
- **Actuality:** reason for selection of event at present moment
- **Unexpectedness:** event was not expected

Originally understood as physical distance between an event and newsroom, the classic news factor of proximity is now conceptualized as physical and psychological distance between event and audience, indicated by cultural, political, or economic ties (15, 16, 59). Analysis of U.S. coverage of international news have identified geographic disparities in print and television, explained by psychological proximity as well as a foreign nation's "prominence" on a global stage (57-60). Below we provide a short sample of such studies:

Jones and colleagues (60) tracked foreign nation visibility in *The New York Times* and NBC Nightly News from 1950-2006, considering geographic proximity, bilateral trade flow, U.S. troop deployment, GDP per capita, and population of countries in relation to the U.S. Across the full timeframe, Russia/USSR and Israel received the most consistent coverage followed by Britain, China, France, Japan, Germany, Iraq, and Mexico. In the post-9/11 period (2002-2006), *The New York Times* gave the most foreign coverage (in descending order) to Iraq, China, France, Israel, Japan, Russia, Britain, Afghanistan, Mexico, and Germany. For *The New York* Times, all tested variables significantly correlated with foreign nation visibility.

In an analysis of U.S. evening newscast coverage of international natural disasters, Adams (15) found T.V. minutes per estimated 1,000 deaths to be far highest for Western Europe (9.2), followed by Eastern Europe (3.6), Latin America (1.02), the Middle East (0.87), and Asia (0.76). Of 16 variables analyzed, three explained 61% of the variation in coverage: number of U.S. tourists (as proxy for cultural proximity and social interest), estimated disaster deaths (as a logarithm), and distance from New York City. The author concluded, for U.S. evening news, "the globe is prioritized so that the death of one Western European equaled three Eastern Europeans equaled 9 Latin Americans equaled 11 Middle Easterners equaled 12 Asians."

Focusing on audience interest rather than actual coverage, Hargrove and Stempel (57) surveyed 1,007 U.S. adults about their interest in 20 hypothetical headlines, which varied by geographic

region, positive/negative overtones, and topic. For foreign regions, interest was highest for stories about the Middle East (45% of respondents), followed by Canada/Mexico (37%), Africa (35%), Western Europe (35%), and Southeast Asia (29%).

text S2. Archaeology in Chinese Media

Although this article focuses on the US media landscape, our reviewers asked for a comparison with science journalism in China. Since the 1950s, there have been Chinese journalistic sources for information on cultural heritage and archaeology-related topics at local and provincial levels. Since 1986, a national-level gazette (*Zhongguo wenwubao* 中国文物报 [*Chinese cultural relics gazette*]) has been published in the form of a weekly newspaper as a major source of information about discoveries and related issues (99). Since 2022 it has been available online (www.zhongguowenwubao.com) as a bi-weekly newsletter. This source almost exclusively covers content from China or, occasionally, research by Chinese scholars abroad. Archaeological finds and related topics are also covered widely by mainstream media sources. Such media coverage is not limited to Chinese research, although most articles do focus on China. In contrast with the U.S., archaeology is overtly a state-sponsored discipline in China.

An exhaustive discussion of Chinese science journalism is beyond the scope of this study, but one salient aspect is the nature of media alerts, press releases, or other forums that alert journalists about recent research. We reached out to a science journalist based in China for additional information. In their experience, some Chinese institutions do create their own press releases. These will provide basic information about a discovery and its importance. The releases are typically distributed on WeChat groups that include journalists and through other channels such as email. Much of this is done through personal networks.

Our analysis found little support for focus on U.S. archaeology by U.S. media. Although the precent coverage was relatively high (38%), in the linear regression models, the U.S. was not more likely to receive coverage than any other country. The statistically significant geographic patterns, we argue, are related to perceived cultural proximity: China/Taiwan in relation to countries relevant to white European or Judeo-Christian history. Therefore, if Chinese media gave more attention to Chinese archaeology, it would not contradict the findings from our study.

fig. S1. Journal outputs by year. Archaeology research papers published in 7 journals from 2015–2020. Note break in vertical axis.

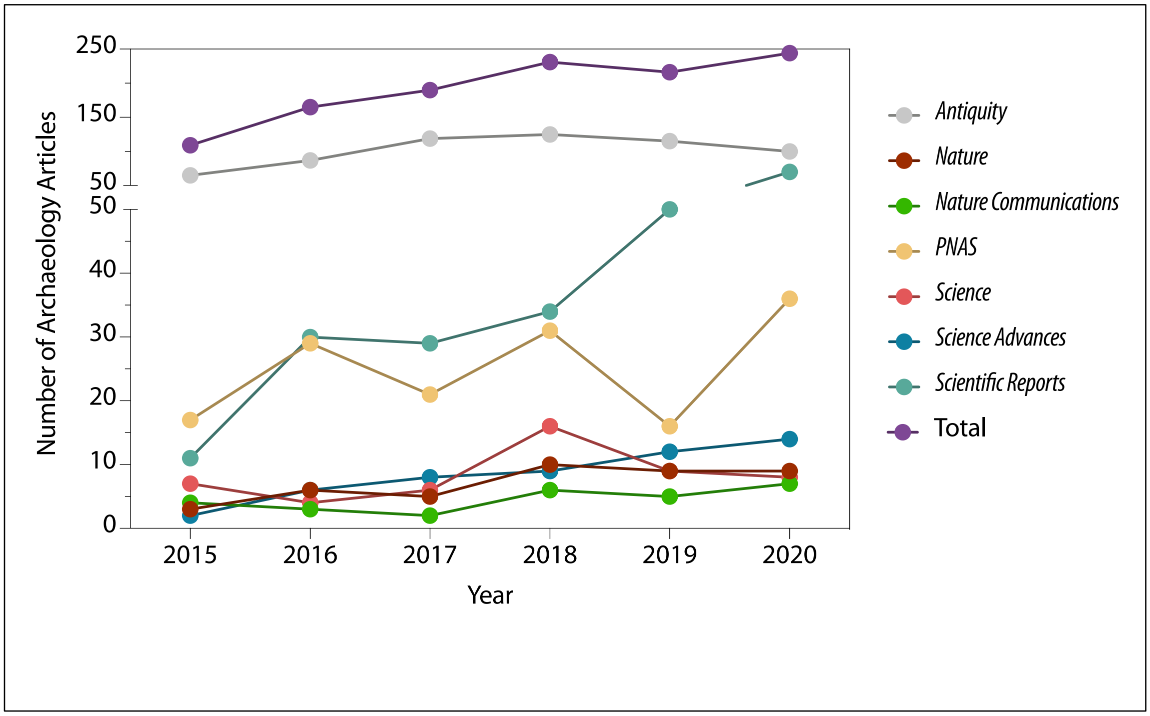

fig. S2. Correlations between U.S. news score and (A) Altmetric attention score or (B) global news score. Each point represents an archaeology research paper, plotted by its respective U.S. news score (sum of mentions in 15 U.S. news sources) and Altmetric attention score or global news score.

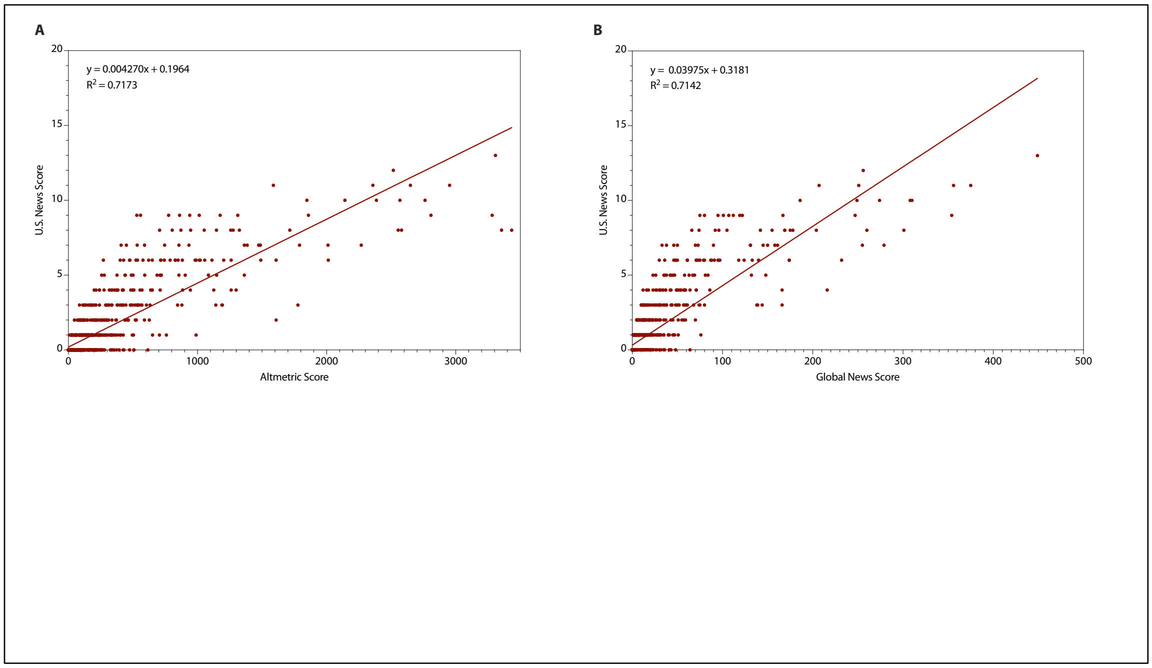

fig. S3. Adjusted odds ratios for U.S. news coverage at continent scale. Results of mixed-effects logistic regression models to calculate adjusted odds ratios (aOR) for U.S. news coverage by each covariable. Adjusted odds ratios were relative to *Antiquity* for journal, articles that did not concern Paleolithic Archaeology, 2015 for year, Asia for continent, and no inclusion in EurekAlert!. The 95% confidence intervals of statistically significant aOR are colored. The EurekAlert!/No EurekAlert! columns report the number and percentage of articles with U.S. news coverage (U.S. news scores <0) for a respective covariable, with and without inclusion in this press release service.

	Eurek Alert		No EurekAlert		Multivariate Model		
	News Coverage	(%)	News Coverage	(%)	aOR	95% CI	p-value
**Journal**	**158**	**100%**	**209**	**100%**			
Antiquity	14	9%	98	47%	1.00	*(Reference)*	
Nature	26	16%	15	7%	110.49	14.14-863.26	P=0.00
Nature Communications	9	6%	4	2%	1.85	0.75-4.50	P=0.17
PNAS	39	25%	34	16%	2.47	1.57-3.88	P=0.00
Science	28	18%	18	9%	29.16	9.83-86.48	P=0.00
Science Advances	18	11%	19	9%	6.84	3.39-13.83	P=0.00
Scientific Reports	24	15%	21	10%	0.89	0.59-1.36	P=0.60
**Paleoarchaeology**	**158**	**100%**	**209**	**100%**			
Not paleolithic archaeology	104	66%	164	78%	1.00	*(Reference)*	
Paleolithic archaeology	54	34%	45	22%	1.53	1.02-2.28	P=0.04
**Publication Year**	**158**	**100%**	**209**	**100%**			
2015	9	6%	25	12%	1.00	*(Reference)*	
2016	17	11%	27	13%	0.74	0.39-1.39	P=0.39
2017	22	14%	36	17%	1.03	0.56-1.87	P=0.56
2018	34	22%	48	23%	0.97	0.54-1.74	P=0.54
2019	33	21%	21	10%	1.09	0.60-1.96	P=0.60
2020	43	27%	31	15%	0.78	0.43-1.41	P=0.43
**Continent**	**143**	**100%**	**195**	**100%**			
Asia	48	34%	68	35%	1.00	*(Reference)*	
Europe	30	21%	55	28%	0.94	0.64-1.39	P=0.76
Africa	22	15%	21	11%	1.07	0.62-1.85	P=0.82
North America	23	16%	28	14%	1.50	0.90-2.49	P=0.12
South America	8	6%	16	8%	1.62	0.85-3.10	P=0.14
Australia, Oceania	9	6%	4	2%	0.85	0.36-2.01	P=0.71
Global	3	2%	3	2%	0.36	0.13-1.04	P=0.06
**Eurek Alert**	**158**	**100%**	**209**	**100%**			
No Eurek Alert	NA		209	100%	1.00	*(Reference)*	
Eurek Alert	158	100%	NA		4.09	2.70-6.17	P=0.00

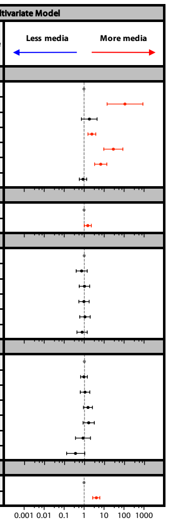

fig. S4. Adjusted odds ratios for U.S. news coverage at subcontinent scale. Results of mixed-effects logistic regression models to calculate adjusted odds ratios for U.S. news coverage by each covariable. Adjusted odds ratios were relative to *Antiquity* for journal, articles that did not concern Paleolithic Archaeology, 2015 for year, East Asia for subcontinent, and no inclusion in EurekAlert!. The 95% confidence intervals of statistically significant aOR are colored. The EurekAlert!/No EurekAlert! columns report the number and percentage of articles with U.S. news coverage (U.S. news scores <0) for a respective covariable, with and without inclusion in this press release service.

	Eurek Alert		No EurekAlert		Multivariate Model		
	News Coverage	(%)	News Coverage	(%)	aOR	95% CI	p-value
**Journal**	**158**	**100%**	**209**	**100%**			
Antiquity	14	9%	98	47%	1.00	*(Reference)*	
Nature	26	16%	15	7%	123.89	16.10-953.44	P=0.00
Nature Communications	9	6%	4	2%	2.12	0.86-5.25	P=0.10
PNAS	39	25%	34	16%	2.78	1.75-4.40	P=0.00
Science	28	18%	18	9%	32.08	10.72-95.94	P=0.00
Science Advances	18	11%	19	9%	7.53	3.66-15.47	P=0.00
Scientific Reports	24	15%	21	10%	0.94	0.61-1.45	P=0.78
**Paleolithic Archaeology**	**158**	**100%**	**209**	**100%**			
Not paleolithic archaeology	104	66%	164	78%	1.00	*(Reference)*	
Paleolithic archaeology	54	34%	45	22%	1.60	1.06-2.42	P=0.03
**Publication Year**	**158**	**100%**	**209**	**100%**			
2015	9	6%	25	12%	1.00	*(Reference)*	
2016	17	11%	27	13%	0.74	0.39-1.40	P=0.35
2017	22	14%	36	17%	0.96	0.52-1.77	P=0.90
2018	34	22%	48	23%	1.02	0.56-1.86	P=0.95
2019	33	21%	21	10%	1.16	0.64-2.11	P=0.63
2020	43	27%	31	15%	0.77	0.42-1.41	P=0.40
**Subcontinent**	**132**	**100%**	**186**	**100%**			
Western Europe	23	17%	46	25%	1.00	0.38-2.47	P=0.38
Southwest Asia	13	10%	29	16%	1.86	0.94-3.68	P=0.07
East Asia	11	8%	18	10%	1.00	*(Reference)*	
Sub-Saharan Africa	19	14%	14	8%	1.38	0.63-3.05	P=0.42
North America	14	11%	12	6%	1.01	0.46-2.22	P=0.97
Southeast Asia	10	8%	16	9%	1.53	0.67-3.51	P=0.31
Central Asia	12	9%	5	3%	0.72	0.29-1.79	P=0.47
South America	8	6%	16	9%	1.99	0.90-4.41	P=0.09
Eastern Europe	3	2%	4	2%	0.57	0.20-1.68	P=0.31
Central America	9	7%	13	7%	4.02	1.66-9.75	P=0.002
North Africa	2	2%	9	5%	1.93	0.72-5.14	P=0.19
Australia, Oceania	8	6%	4	2%	0.90	0.33-2.45	P=0.83
**Eurek Alert**	**158**	**100%**	**209**	**100%**			
No Eurek Alert	NA		209	100%	1.00	*(Reference)*	
Eurek Alert	158	100%	NA		4.31	2.83-6.57	P=0.00

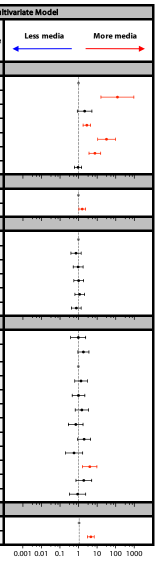

fig. S5. Assignment of countries to subcontinents. Assignments reflect geographic location or archaeologically relevant groupings, rather than modern geopolitical associations. For India, articles were assigned to Southwest Asia if the research concerned the Indus Valley Civilization; other archaeological research in India was assigned to Southeast Asia. Created with MapChart.

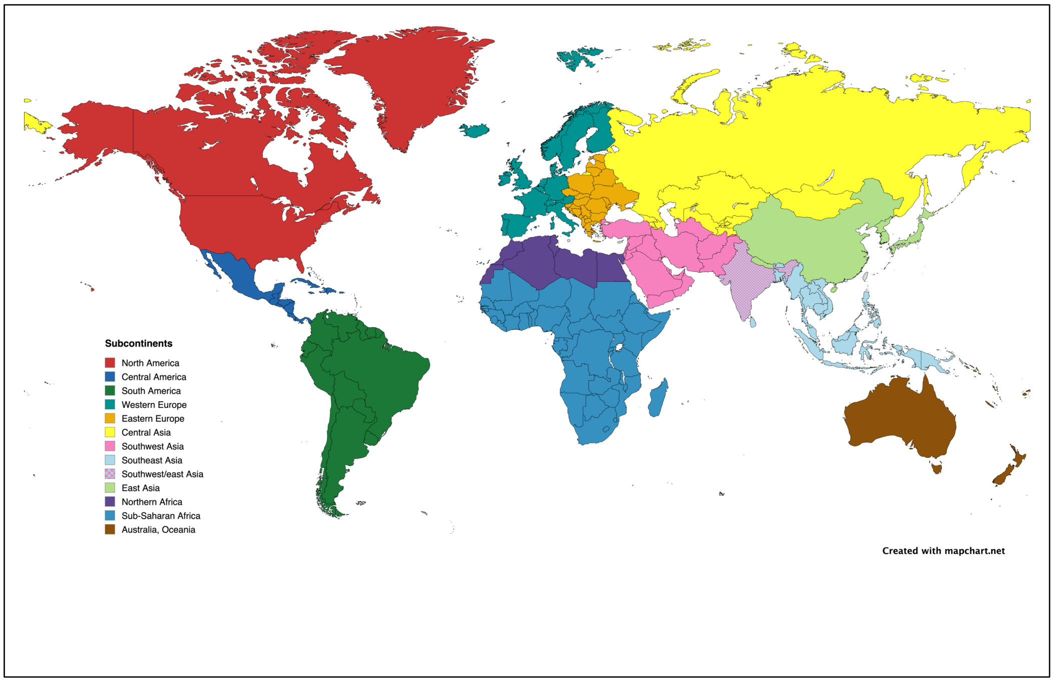

fig. S6. Pairwise comparison of coverage for countries. Colored cells indicate a column country was less (red) or more (blue) likely to receive U.S. news coverage than a row country. Statistically significant results are in the brighter colors. Non-significant results are in the faded colors.

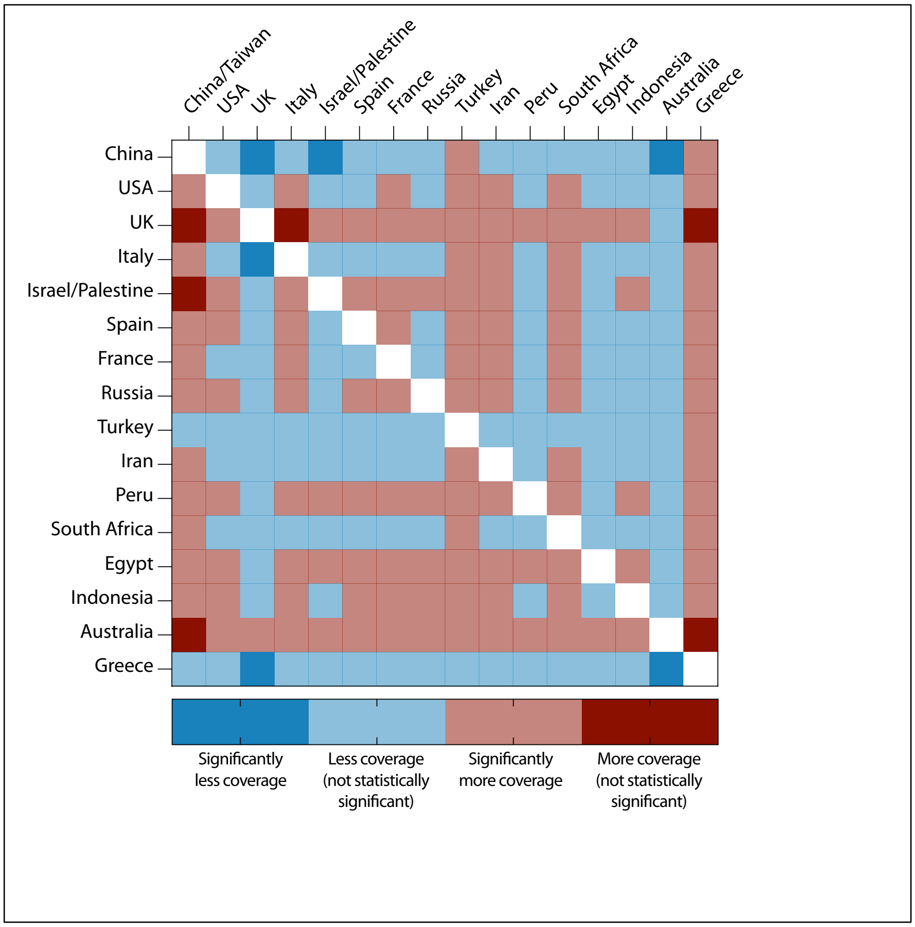

table S1. Archaeology research papers published across 6-year span in 7 journals.

Year	*Antiquity*	*Scientific Reports*	PNAS	*Science Advances*	*Science*	*Nature*	*Nature Comm.*	Total
2015	65	11	17	2	7	3	4	109
2016	87	30	29	6	4	6	3	165
2017	119	29	21	8	6	5	2	190
2018	125	34	31	9	16	10	6	231
2019	115	50	16	12	9	9	5	216
2020	100	70	36	14	8	9	7	244
Total	611	224	150	51	50	42	27	1155

table S2. Research papers receiving top scores for Altmetric global news mentions.

Altmetric Global News Mentions					
rank	research paper	location	score	Paleo Arch	Eurek Alert!
1	The earliest modern humans outside Africa *Science*	Israel/Palestine	449	✓	✓
2	Human origins in a southern African palaeo-wetland and first migrations *Nature*	Botswana, South Africa	375	✓	
3	The age of the hominin fossils from Jebel Irhoud, Morocco, and the origins of the Middle Stone Age *Nature*	Morocco	356	✓	✓
4	U-Th dating of carbonate crusts reveals Neandertal origin of Iberian cave art *Science*	Spain	354	✓	✓
5	Earliest hunting scene in prehistoric art *Nature*	Indonesia	310	✓	
6	An abstract drawing from the 73,000-year-old levels at Blombos Cave, South Africa *Nature*	South Africa	308	✓	✓
7	Archaeobotanical evidence reveals the origins of bread 14,400 years ago in northeastern Jordan PNAS	Jordon	301		✓
8	Revealing a 5,000-y-old beer recipe in China PNAS	China	279		
9	Apidima Cave fossils provide earliest evidence of Homo sapiens in Eurasia *Nature*	Greece	274	✓	
10	Female hunters of the early Americas *Science Advances*	Peru	260		✓

table S3. Research papers receiving top U.S. news scores.

U.S. News Score					
rank	research paper	location	score	Paleo Arch	Eurek Alert!
1	The earliest modern humans outside Africa *Science*	Israel/Palestine	13	✓	✓
2	A 130,000-year-old archaeological site in southern California, USA *Nature*	USA	12	✓	✓
3	The age of the hominin fossils from Jebel Irhoud, Morocco, and the origins of the Middle Stone Age *Nature*	Morocco	11	✓	✓
3	Human origins in a southern African palaeo-wetland and first migrations *Nature*	Botswana, South Africa	11	✓	✓
3	Evidence of human occupation in Mexico around the Last Glacial Maximum *Nature*	Mexico	11	✓	✓
3	Human occupation of northern Australia by 65,000 years ago *Nature*	Australia	11	✓	✓
7	Earliest hunting scene in prehistoric art *Nature*	Indonesia	10	✓	
7	An abstract drawing from the 73,000-year-old levels at Blombos Cave, South Africa *Nature*	South Africa	10	✓	✓
7	Apidima Cave fossils provide earliest evidence of Homo sapiens in Eurasia *Nature*	Greece	10	✓	
7	A late Middle Pleistocene Denisovan mandible from the Tibetan Plateau *Nature*	China	10	✓	✓
7	The origins of cannabis smoking: Chemical residue evidence from the first millennium BCE in the Pamirs *Science Advances*	China	10		✓

data S1. Coded research outputs and media coverage. Research outputs from Altmetric Explorer search were cleaned to include only peer reviewed archaeology research published between Jan 1, 2015 and Dec 31, 2020 in seven journals. Altmetric data includes attention score, global news mentions, appearance in 15 U.S. news outlets, appearance on EurekAlert!, and Twitter mentions. Using article text, we manually coded for geographic location, time period, and focus on topics of Paleolithic Archaeology, ancient DNA, or environmental archaeology.

data S2. Altmetric research outputs. In September 2021 data were downloaded from an Altmetric Explorer search under field of research "archaeology" for "articles" published between January 1, 2015 and December 31, 2020 in *Antiquity*, *Science, Nature, Science Advances, Nature Communications, Scientific Reports,* and *Proceedings of the National Academy of Sciences* (PNAS).

data S3. Altmetric news mentions. The data are Altmetric's highlights only news mentions for the research outputs resulting from our search in Altmetric Explorer in September 2021.

data S4. Mixed-effects logistic regression model outputs for individual news outlets and EurekAlert!. Run in STATA/BE 17.0, mixed-effects logistic regression models estimate odds ratios for an article about different countries to appear in each of 15 U.S. news outlets, controlling for Paleolithic Archaeology, journal, year, and EurekAlert! inclusion, with respect to China/Taiwan. Models also estimate odds ratios for an article to have a press release on EurekAlert! by region, controlling for Paleolithic Archaeology, journal, and year, with respect to Asia, East Asia, and China/Taiwan.

data S5. Mixed-effects logistic regression model outputs with specialist and general science journals separated. In STATA/BE 17.0, mixed-effects logistic regression models were run on our dataset separated into articles from the specialist journal (*Antiquity*) and general science journals. Models estimate the odds ratios of research from a particular region to receive coverage, controlling for Paleolithic Archaeology, journal, year, and EurekAlert! inclusion, with respect to Asia, East Asia, and China/Taiwan.

data S6. Mixed-effects logistic regression model outputs with North America, U.S. as reference. Run in STATA/BE 17.0, mixed-effects logistic regression models estimate odds ratios for articles from a given region to receive coverage controlling for Paleolithic Archaeology, journal, year, and EurekAlert! inclusion, with respect to North America (at continent and subcontinent levels) and U.S.

REFERENCES AND NOTES

1. B. Freeling, Z. A. Doubleday, S. D. Connell, How can we boost the impact of publications? Try better writing. *Proc. Natl. Acad. Sci. U.S.A.* **116**, 341–343 (2019).

2. L. Bornmann, R. Haunschild, R. Mutz, Growth rates of modern science: a latent piecewise growth curve approach to model publication numbers from established and new literature databases. *Humanit. Soc. Sci. Commun.* **8**, 224 (2021).

3. National Science Board, Publications Output: U.S. Trends and International Comparisons," Science and Engineering Indicators 2021. (National Science Foundation, 2021).

4. B. Aczel, B. Szaszi, A. O. Holcombe, A billion-dollar donation: estimating the cost of researchers' time spent on peer review. *Res. Integr. Peer Rev.* **6**, 14 (2021).

5. P. Plavén-Sigray, G. J. Matheson, B. C. Schiffler, W. H. Thompson, The readability of scientific texts is decreasing over time. *eLife* **6**, e27725 (2017).

6. Pew Research Center, "Pew Research Cener's American Trends Panel" (2021); www. pewresearch.org.

7. M. W. Angler, *Science Journalism: An Introduction* (Routledge New York, 2017).

8. L. Guenther, *Oxford Research Encyclopedia of Communication* (Oxford Univ. Press, 2019).

9. H. Pearson, Careers in Science Journalism and Writing. *Cold Spring Harb. Perspect. Biol.* **9**, a032961 (2017).

10. D. Blum, Science Journalism Grows Up. *Science* **372**, 323 (2021).

11. H. Yu, X. Cao, Y. Wang, Xinwen tidai jiliang zhibiao de fenbu tezheng yanjiu 新闻替代计量指标的分布特征研究 (Distribution Features of News Altmetrics). *J. China Soc. Sci. Tech. Inf.* **39**, 1081–1092 (2020).

12. C. Fleming, *An Introduction to Journalism* (SAGE Publications, Limited, 2006). pp. 4–26.

13. T. Harcup, D. O'Neill, What is News? *Journal. Stud.* **18**, 1470–1488 (2017).

14. P. J. Shoemaker, E. K. Mayfield, Building a Theory of News Content: A Synthesis of Current Approaches. Journalism Monographs Number 103 (Association for Education in Journalism and Mass Communication, 1987).

15. W. C. Adams, Whose Lives Count?: TV Coverage of Natural Disasters. *J. Commun.* **36**, 113–122 (1986).

16. P. J. Shoemaker, J. H. Lee, G. K. Han, A. A. Cohen "Proximity and scope as news values," in *Media Studies: Key issues and debates*, E. Devereux Ed. (SAGE Publications Ltd., 2007), pp. 231–248.

17. F. Badenschier, H. Wormer "Issue selection in science journalism: Towards a special theory of news values for Science News?" in *The Sciences' Media Connection - Public Communication and its Repercussions*, S. Rodder, M. Franzen, P. Weingart, Eds. (Springer Science + Business Media, 2012), pp. 59–85.

18. C. M. Stojanowski, W. N. Duncan, Engaging bodies in the public imagination: Bioarchaeology as social science, science, and humanities. *Am. J. Hum. Biol.* **27**, 51–60 (2015).

19. M. Franzen, Making science news: The press relations of scientific journals and implications for scholarly communication. *Sociol. Sci.* **28**, 333–352 (2012).

20. M. H. Slater, E. R. Scholfield, J. C. Moore, Reporting on science as an ongoing process (or not). *Front. Commun.* **5**, 535474 (2021).

21. N. R. Davidson, C. S. Greene, Analysis of science journalism reveals gender and regional disparities in coverage. *eLife* **28**, RP84855 (2024).

22. H. Peng, M. Teplitskiy, D. Jurgens, Author mentions in science news reveal widespread disparities across name-inferred ethnicities. *Quant. Sci. Stud.* **5**, 351–365 (2024).

23. ResearchAmer!ca. Survey: Most Americans Cannot Name a Living Scientist or a Research Institution (2021); wwww.researchamerica.org/blog/survey-most-americans-cannot-name-a-living-scientist-or-a-research-institution/.

24. D. Barrowclough, *Digging for Hitler: the Nazi archaeologists search for an Aryan past* (Fonthill Media, 2016).

25. S. E. Hakenbeck, Genetics, archaeology and the far right: An unholy trinity. *World Archaeol.* **51**, 517–527 (2019).

26. S. Halmhofer, Did aliens build the pyramids? And other racist theories (Sapiens, 2021); www.sapiens.org/archaeology/pseudoarchaeology-racism/.

27. S. Halmhofer, "Manufacturing history: Atlantis, Aryans, and the use of pseudoarchaeology by the far-right," in *Conspiracy Theories and Extremism in New Times*, C. T. Conner, M. N. Hannah, N. J. MacMurray Eds. (Lexington Books, 2024), pp. 53–81.

28. L. Gershon, "Archaeologists in Egypt discover 3,000-year-old 'Lost Golden City'," *Smithsonian Magazine*, 9 April 2021; www.smithsonianmag.com/smart-news/archaeologists-discover-3400-year-old-egyptian-city-180977471/.

29. S. Raghavan, "A 3,000-year-old 'lost golden city' has been unearthed in Egypt," *The Washington Post*, 8 April 2021; www.washingtonpost.com/world/middle_east/luxor-lost-city-tutankhamun-archaeology/2021/04/08/4d9577d0-9863-11eb-8f0a-3384cf4fb399_story.html.

30. Reuters, Archaeologists Unearth 'Ancient Egyptian Pompeii', *The New York Times*, 8 April 2021; www.nytimes.com/2021/04/08/world/middleeast/egypt-ancient-city.html.

31. Sanxingdui [Sanxingdui Yizhi Jisiqu Kaogu Gongzuodui 三星堆遗址祭祀区考古工作队], Sichuan Guanghan shi Sanxingdui yizhi jisiqu 四川广汉市三星堆遗址祭祀去 (The sacrificial precinct of Sanxingdui in Guanghan City, Sichuan). *Kaogu* 考古 [*Archaeology*] **2002**, 15–33 (2022).

32. Cai Chunlin 蔡纯琳. (CCTV, 2021), Sanxingdui "Shang Xin" wenwu 500 yu jian 三星堆"上新"文物500余件 (More than 500 cultural relics "new" in Sanxingdui) (2021).

33. Gu Yue 谷玥, “Sanxingdui kaogu xin faxian: Shenmi mu xia qidong fajue zuida qingtong zun bei tiqu 三星堆考古新发现:神秘木匣启动发掘 最大青铜尊被提取 (New archaeological discoveries in Sanxingdui: Excavation of the mysterious wooden box begins and the largest bronze statue is extracted),” (Xinhua, 2021); www.xinhuanet.com/politics/2021--2003/2022/c_1127238356.htm.

34. Wang Mengmeng 王萌萌, Sanxingdui yizhi "zai jing tianxia" xian yi chutu zhongyao wenwu 500 yu jian 三星堆遗址“再惊天下” 现已出土重要文物500余件 (The Sanxingdui site "shocked the world again" and more than 500 important cultural relics have been unearthed),” *Xinhua News*, 2021; www.xinhuanet.com/local/2021-2003/2020/c_1127234295.htm.

35. H. Wu, Ye Ruolin, “The mysterious ancient city that’s rewriting Chinese history,” *Sixth Tone*, 7 July 2021.

36. R. K. Flad, “It’s a golden age for Chinese archaeology—and the West is ignoring it,” *The Washington Post*, 11 May 2021; www.washingtonpost.com/outlook/2021/05/11/chinese-archaeology-egyptian-bias-sanxingdui/.

37. A. E. Williams, Altmetrics: An overview and evaluation. *Online Inform. Rev.* **41**, 311–317 (2017).

38. A. Fleerackers, L. Nehring, L. A. Maggio, A. Enkhbayar, L. Moorhead, J. P. Alperin, Identifying science in the news: An assessment of the precision and recall of Altmetric.com news mention data. *Scientometrics* **127**, 6109–6123 (2022).

39. J.-L. Ortega, Altmetrics data providers: A meta-analysis review of the coverage of metrics and publication. *Prof. Inf.* **29**, e290107 (2020).

40. D. Torres-Salinas, J. Gorraiz, N. Robinson-Garcia, The insoluble problems of books: What does Altmetric.com have to offer? *Aslib J. Inf. Manag.* **70**, 691–707 (2018).

41. G. Barata, Por métricas alternativas mais relevantes para a América Latina. *Transinformação* **31**, e190031 (2019).

42. H. Yu, X. Yu, X. Cao, How accurate are news mentions of scholarly output? A content analysis. *Scientometrics* **127**, 4075–4096 (2022).

43. J. Beck, E. Gjesfjeld, S. Chrisomalis, Prestige or Perish: Publishing decisions in academic archaeology. *Am. Antiq.* **86**, 669–695 (2021).

44. M. Kowal, P. Sorokowski, E. Kulczycki, A. Żelaźniewicz, The impact of geographical bias when judging scientific studies. *Scientometrics* **127**, 265–273 (2022).

45. I. Wachtel, R. Zidon, S. Garti, G. Shelach-Lavi, Predictive modeling for archaeological site locations: Comparing logistic regression and maximal entropy in north Israel and north-east China. *J. Archaeol. Sci.* **92**, 28–36 (2018).

46. J. Suleski, M. Ibaraki, Scientists are talking, but mostly to each other: A quantitative analysis of research represented in mass media. *Public Underst. Sci.* **19**, 115–125 (2010).

47. V. de Semir, C. Ribas, G. Revuelta, Press releases of science journal articles and subsequent newspaper stories on the same topic. *JAMA* **280**, 294–295 (1998).

48. J. E. Stryker, Reporting medical information: Effects of press releases and newsworthiness on medical journal articles' visibility in the news media. *Prev. Med.* **35**, 519–530 (2002).

49. P. Sumner, S. Vivian-Griffiths, J. Boivin, A. Williams, L. Bott, R. Adams, C. A. Venetis, L. Whelan, B. Hughes, C. D. Chambers, Exaggerations and caveats in press releases and health-related science news. *PLOS ONE* **11**, e0168217 (2016).

50. S. Lemke, M. Brede, S. Rotgeri, I. Peters, Research articles promoted in embargo e-mails receive higher citations and altmetrics. *Scientometrics* **127**, 75–97 (2022).

51. A. Arranz-Otaegui, L. Gonzalez Carretero, M. N. Ramsey, D. Q. Fuller, T. Richter, Archaeobotanical evidence reveals the origins of bread 14,400 years ago in northeastern Jordan. *Proc. Natl. Acad. Sci. U.S.A.* **115**, 7925–7930 (2018).

52. P. McGovern, M. Jalabadze, S. Batiuk, M. P. Callahan, K. E. Smith, G. R. Hall, E. Kvavadze, D. Maghradze, N. Rusishvili, L. Bouby, O. Failla, G. Cola, L. Mariani, E. Boaretto, R.

Bacilieri, P. This, N. Wales, D. Lordkipanidze, Early Neolithic wine of Georgia in the South Caucasus. *Proc. Natl. Acad. Sci. U.S.A.* **114**, E10309–E10318 (2017).

53. M. Ren, Z. Tang, X. Wu, R. Spengler, H. Jiang, Y. Yang, N. Boivin, The origins of cannabis smoking: Chemical residue evidence from the first millennium BCE in the Pamirs. *Sci. Adv.* **5**, 1–8 (2019).

54. J. Wang, L. Liu, T. Ball, L. Yu, Y. Li, F. Xing, Revealing a 5,000-y-old beer recipe in China. *Proc. Natl. Acad. Sci. U.S.A.* **113**, 6444–6448 (2016).

55. R. Haas, J. Watson, T. Buonasera, J. Southon, J. C. Chen, S. Noe, K. Smith, C. V. Llave, J. Eerkens, G. Parker, Female hunters of the early Americas. *Sci. Adv.* **6**, eabd0310 (2020).

56. A. G. Ioannidis, J. Blanco-Portillo, K. Sandoval, E. Hagelberg, J. F. Miquel-Poblete, J. V. Moreno-Mayar, J. E. Rodríguez-Rodríguez, C. D. Quinto-Cortés, K. Auckland, T. Parks, K. Robson, A. V. S. Hill, M. C. Avila-Arcos, A. Sockell, J. R. Homburger, G. L. Wojcik, K. C. Barnes, L. Herrera, S. Berríos, M. Acuña, E. Llop, C. Eng, S. Huntsman, E. G. Burchard, C. R. Gignoux, L. Cifuentes, R. A. Verdugo, M. Moraga, A. J. Mentzer, C. D. Bustamante, A. Moreno-Estrada, Native American gene flow into Polynesia predating Easter Island settlement. *Nature* **583**, 572–577 (2020).

57. T. Hargrove, G. H. Stempel III, Exploring Reader Interest In International News. *Newsp. Res. J.* **23**, 46–51 (2002).

58. S. Jones, Television News: Geographic and Source Biases, 1982–2004. *Int. J. Commun.* **2**, 223–252 (2008).

59. D. C. Whitney, M. Fritzler, S. Jones, S. Mazzarella, L. Rakow, Geographic and Source Biases in Network Television News 1982-1984. *J. Broadcast. Electron. Media* **33**, 159–174 (1989).

60. T. M. Jones, P. V. Aelst, R. Vliegenthart, Foreign Nation Visibility in U.S. News Coverage: A Longitudinal Analysis (1950-2006). *Communic. Res.* **40**, 417–436 (2013).

61. F. Chen, F. Welker, C.-C. Shen, S. E. Bailey, I. Bergmann, S. Davis, H. Xia, H. Wang, R. Fischer, S. E. Freidline, T.-L. Yu, M. M. Skinner, S. Stelzer, G. Dong, Q. Fu, G. Dong, J.

Wang, D. Zhang, J.-J. Hublin, A late Middle Pleistocene Denisovan mandible from the Tibetan Plateau. *Nature* **569**, 409–412 (2019).

62. Z.-Y. Li, X.-J. Wu, L.-P. Zhou, W. Liu, X. Gao, X.-M. Nian, E. Trinkaus, Late Pleistocene archaic human crania from Xuchang, China. *Science* **355**, 969–972 (2017).

63. W. Liu, M. Martinón-Torres, Y. Cai, S. Xing, H. Tong, S. Pei, M. J. Sier, X. Wu, R. L. Edwards, H. Cheng, Y. Li, X. Yang, J. M. B. de Castro, X. Wu, The earliest unequivocally modern humans in southern China. *Nature* **526**, 696–699 (2015).

64. A. Fuentes, R. R. Ackermann, S. Athreya, D. Bolnick, T. Lasisi, S.-H. Lee, S.-A. McLean, R. Nelson, AAPA Statement on Race and Racism. *Am. J. Phys. Anthropol.* **169**, 400–402 (2019).

65. ASNE, Table B - Minority Employment by Race and Job Category (2018); http://asne.org/content.asp?contentid=416.

66. E. Grieco, Newsroom employees are less diverse than U.S. workers overall, Pew Research Center, (Pew Research Center, 2018); www.pewresearch.org/short-reads/2018/11/02/newsroom-employees-are-less-diverse-than-u-s-workers-overall/.

67. NASW (National Association of Science Writers), NASW 2021 Diversity Report (2022); www.nasw.org/article/nasw-2021-diversity-report.

68. Pew Research Center, Modeling the Future of Religion in America (2022); www.pewresearch.org/religion/2022/09/13/modeling-the-future-of-religion-in-america/.

69. J. Huang, R. Liu, Xenophobia in America in the age of coronavirus and beyond. *J. Vasc. Interv. Radiol.* **31**, 1187–1188 (2020).

70. C. Huang, L. Silver, L. Clancy, Americans Remain Critical of China (Pew Research Center, 2024); www.pewresearch.org/global/2024/05/01/americans-remain-critical-of-china/.

71. A. Kingdon, Hooked on Classics: The Far Right's Appropriation of Ancient Greece and Rome The World White Web: Uncovering the Hidden Meanings of Online Far-Right Propaganda (Springer Nature, 2024), pp. 69–91.

72. J. M. Gero, D. Lacy, M. L. Blakey, Eds. *The Sociopolitics of Archaeology* (University of Massachusetts, 1983).

73. M. L. Blakey "Socio-political bias and ideological production in historical archaeology," in *The Sociopolitics of Archaeology*, J. M. Gero, D. Lacy, M. L. Blakey, Eds. (1983), pp. 5–16.

74. B. G. Trigger, Alternative Archaeologies: Nationalist, Colonialist, Imperialist. *Man* **19**, 355–370 (1984).

75. M. R. Banaji, A. G. Greenwald, *Blindspot: Hidden Biases of Good People* (Delacorte Press, 2013).

76. T. Devos, M. R. Banaji, American = White? *J. Pers. Soc. Psychol.* **88**, 447–466 (2005).

77. C. Colwell-Chanthaphonh, Publishing the past: Gender and patterns of authorship in academic and public archaeology journals. *Grad. J. Soc. Sci.* **1**, 117–143 (2004).

78. T. J. Fulkerson, S. Tushingham, Who dominates the discourses of the past? gender, occupational affiliation, and multivocality in north american archaeology publishing. *Am. Antiq.* **84**, 379–399 (2019).

79. L. E. Heath-Stout, Who writes about archaeology? an intersectional study of authorship in archaeological journals. *Am. Antiq.* **85**, 407–426 (2020).

80. S. R. Hutson, J. Johnson, S. Price, D. Record, M. Rodriguez, T. Snow, T. Stocking, Gender, institutional inequality, and institutional diversity in archaeology articles in major journals and Sapiens. *Am. Antiq.* **88**, 326–343 (2023).

81. J. M. Gero, D. Root. "Public presentations and private concerns: Archaeology in the pages of National Geographic," in *The Politics of the Past*, P. W. Gathercole, D. Lovwenthal, Eds. (Unwin Hyman, 1990), pp. 19–37.

82. R. H. Fritze, *Egyptomania: A History of Fascination, Obsession and Fantasy* (Reaktion Books, 2016).

83. B. Brier, *Egyptomania: Our Three Thousand Year Obsession with the Land of the Pharaohs* (Palgrave Macmillan, 2013).

84. S. An, Asian Americans in American History: An AsianCrit Perspective on Asian American Inclusion in State U.S. History Curriculum Standards. *Theory Res. Soc. Educ.* **44**, 244–276 (2016).

85. A. L. Brown, W. Au, Race, memory, and master narratives: A critical essay on U.S. curriculum history. *Curr. Inq.* **44**, 358–389 (2014).

86. C. Seeger, T. Mitchell Patterson, M. G. Paz, Reckoning with white supremacy and anti-Black racism in the Virginia US history standards. *Curr. Inq.* **53**, 268–289 (2023).

87. D. M. White, The "gate keeper": A case study in the selection of news. *Journal. Q.* **27**, 383–396 (1950).

88. T. Amano, V. Ramírez-Castañeda, V. Berdejo-Espinola, I. Borokini, S. Chowdhury, M. Golivets, J. D. González-Trujillo, F. Montaño-Centellas, K. Paudel, R. L. White, D. Veríssimo, The manifold costs of being a non-native English speaker in science. *PLoS Biol.* **21**, e3002184 (2023).

89. J. A. Bol, A. Scheffel, N. Zia, A. Meghani, How to address the geographical bias in academic publishing. *BMJ Glob. Health* **8**, e013111 (2023).

90. M. Cabrera, I. Saraiva, Principales problemáticas de las publicaciones científicas: un análisis en perspectiva latinoamericana. *E-Cienc. Inf.* **12**, 188–210 (2022).

91. S. Canagarajah, Language diversity in academic writing: toward decolonizing scholarly publishing. *Journal of Multicultural Discourses* **17**, 107–128 (2022).

92. G. González-Alcaide, J. C. Valderrama-Zurián, R. Aleixandre-Benavent, The impact factor in non-English-speaking countries. *Scientometrics* **92**, 297–311 (2012).

93. M. Skopec, H. Issa, J. Reed, M. Harris, The role of geographic bias in knowledge diffusion: A systematic review and narrative synthesis. *Res. Integr. Peer Rev.* **5**, 1–14 (2020).

94. N. C. Kawa, J. A. Clavijo, J. L. Michelangeli, D. G. Clark, C. McCarty, The social network of US academic anthropology and its inequalities. *Am. Anthropol.* **121**, 14–29 (2019).

95. S. Zhang, K. H. Wapman, D. B. Larremore, A. Clauset, Labor advantages drive the greater productivity of faculty at elite universities. *Sci. Adv.* **8**, eabq7056 (2022).

96. L. Helmuth, "Pitching errors: How not to pitch," in *The Craft of Science Writing*, S. Carpenter, Ed. (The Open Notebook Inc., 2020).

97. J. Rosen, "Beyond the Press-Release Rate Race: Fresh Ways to Cover Science News" (The Open Notebook, 2015); www.theopennotebook.com/2015/07/28/fresh-ways-to-cover-science-news/.

98. E. Sohn, "Finding ideas," in *The Science Writers Handbook*, T. Hayden, M. Nijhuis, Eds. (Da Capo Press, 2013), pp. 9–22.

99. L. V. Falkenhausen, Serials on Chinese Archaeology Published in the People's Republic of China: A Bibliographic Survey. *Early China* **17**, 247–295 (1992).
