# Supplementary material for: Regional disparities in US media coverage of archaeology research
Source: Sci Adv. 2025 Jul 2;11(27):eadt5435. doi: 10.1126/sciadv.adt5435 (PMC12219491; doi:10.1126/sciadv.adt5435)
Supplement: Supplementary file 2 — Data S1 to S6 [file sciadv.adt5435_data_s1_to_s6.zip › adt5435_data_s4.pptx]

## Slide 1
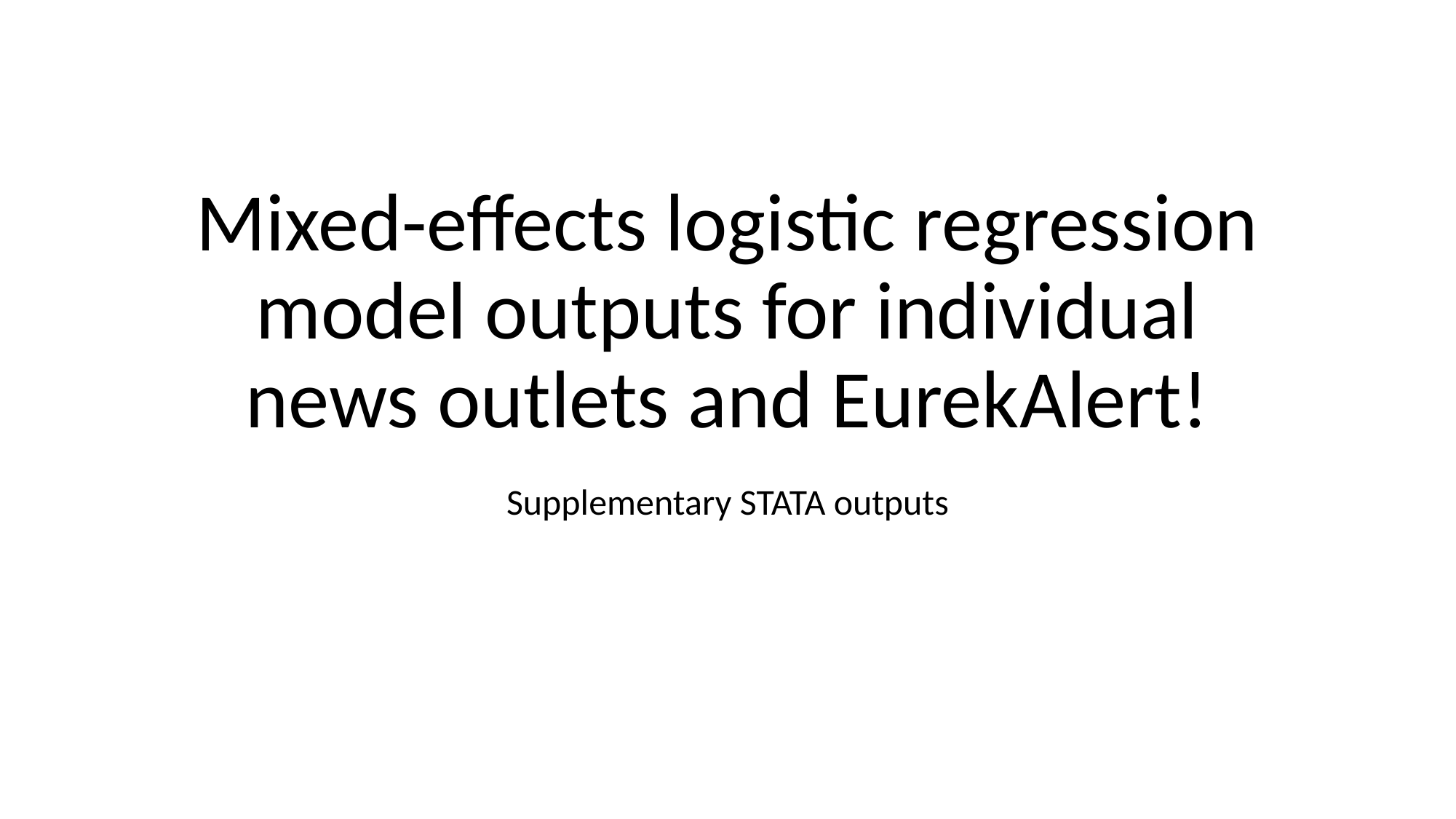

# Mixed-effects logistic regression model outputs for individual news outlets and EurekAlert!
Supplementary STATA outputs

## Slide 2
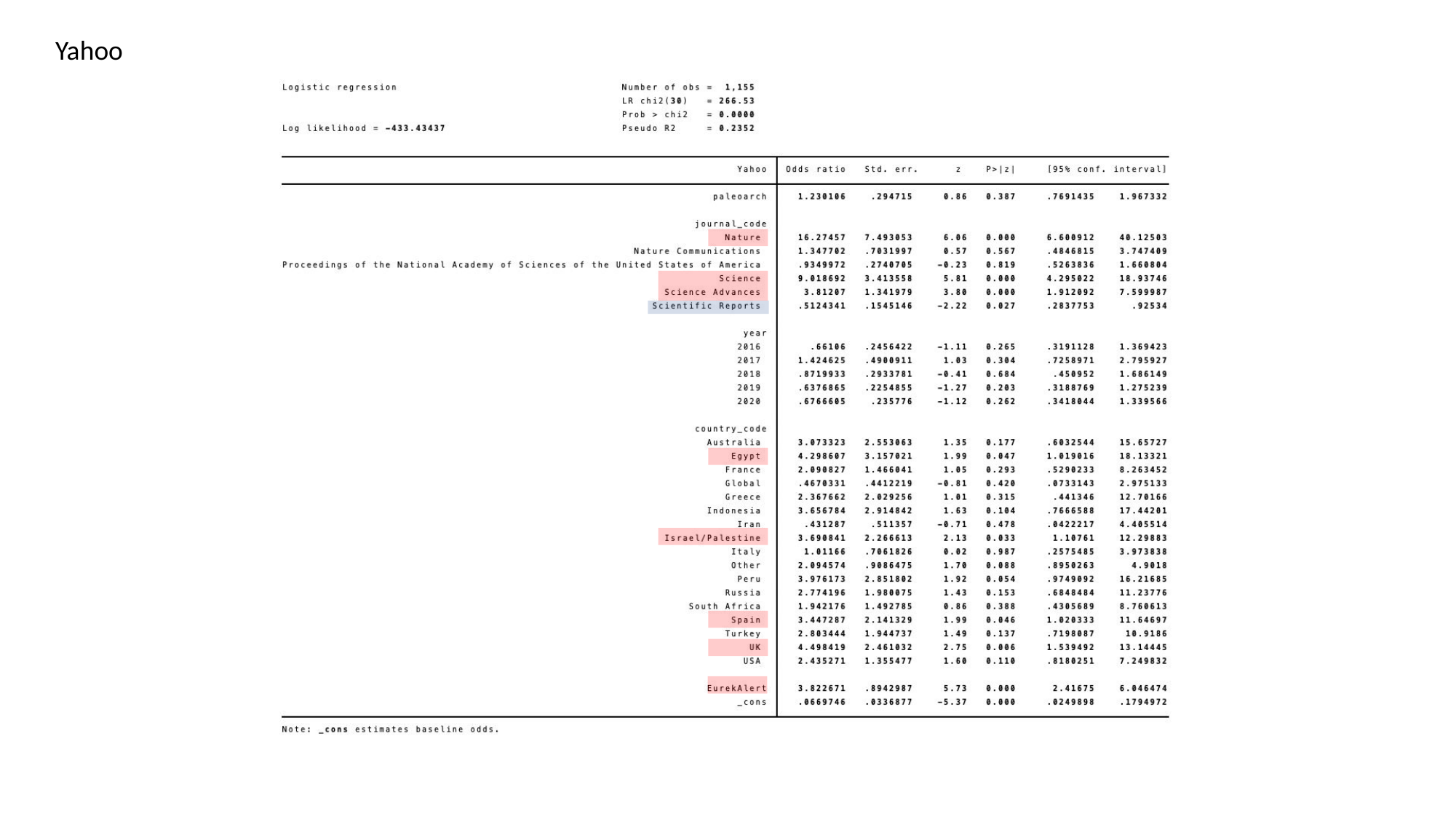

Yahoo

## Slide 3
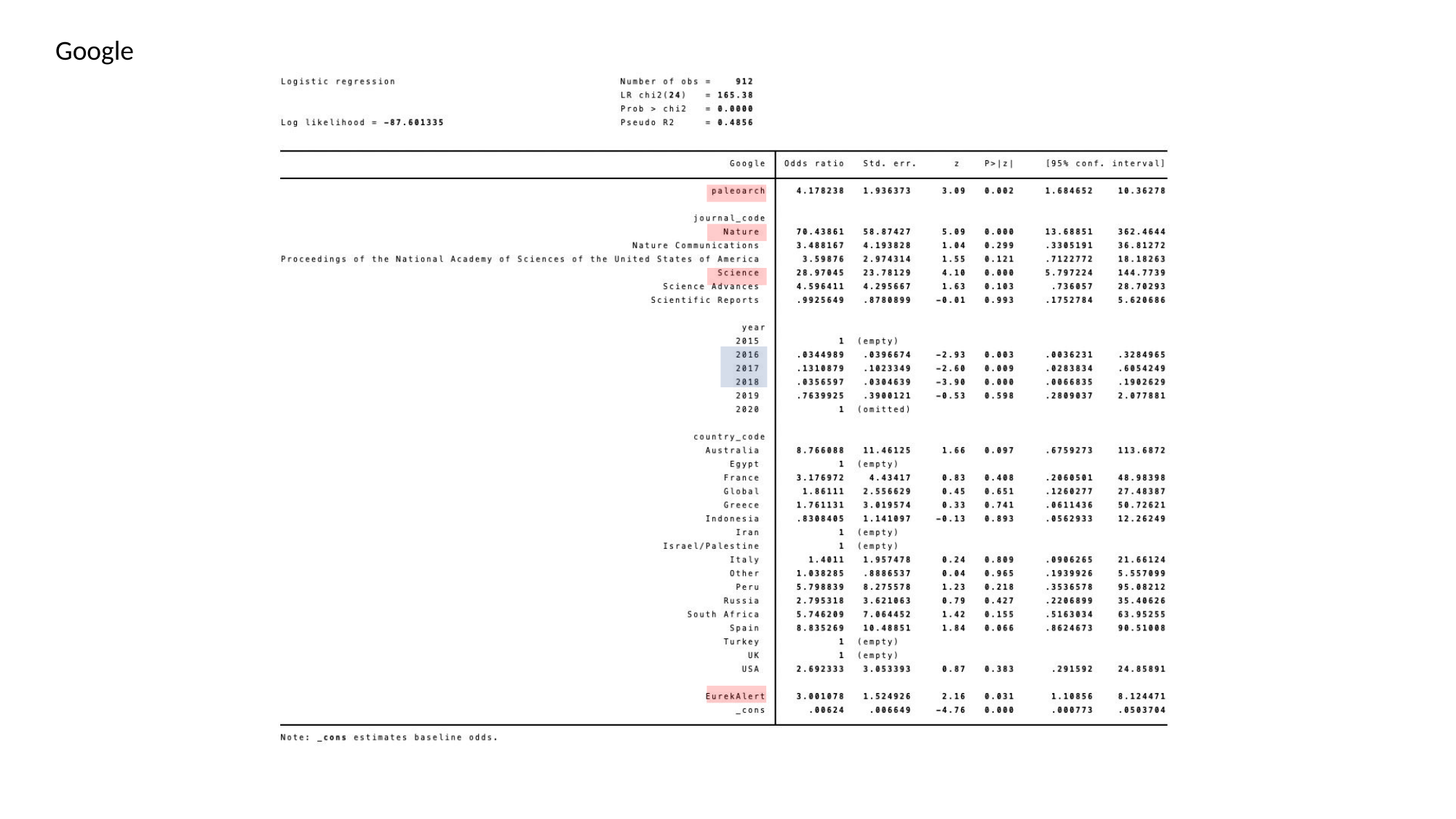

Google

## Slide 4
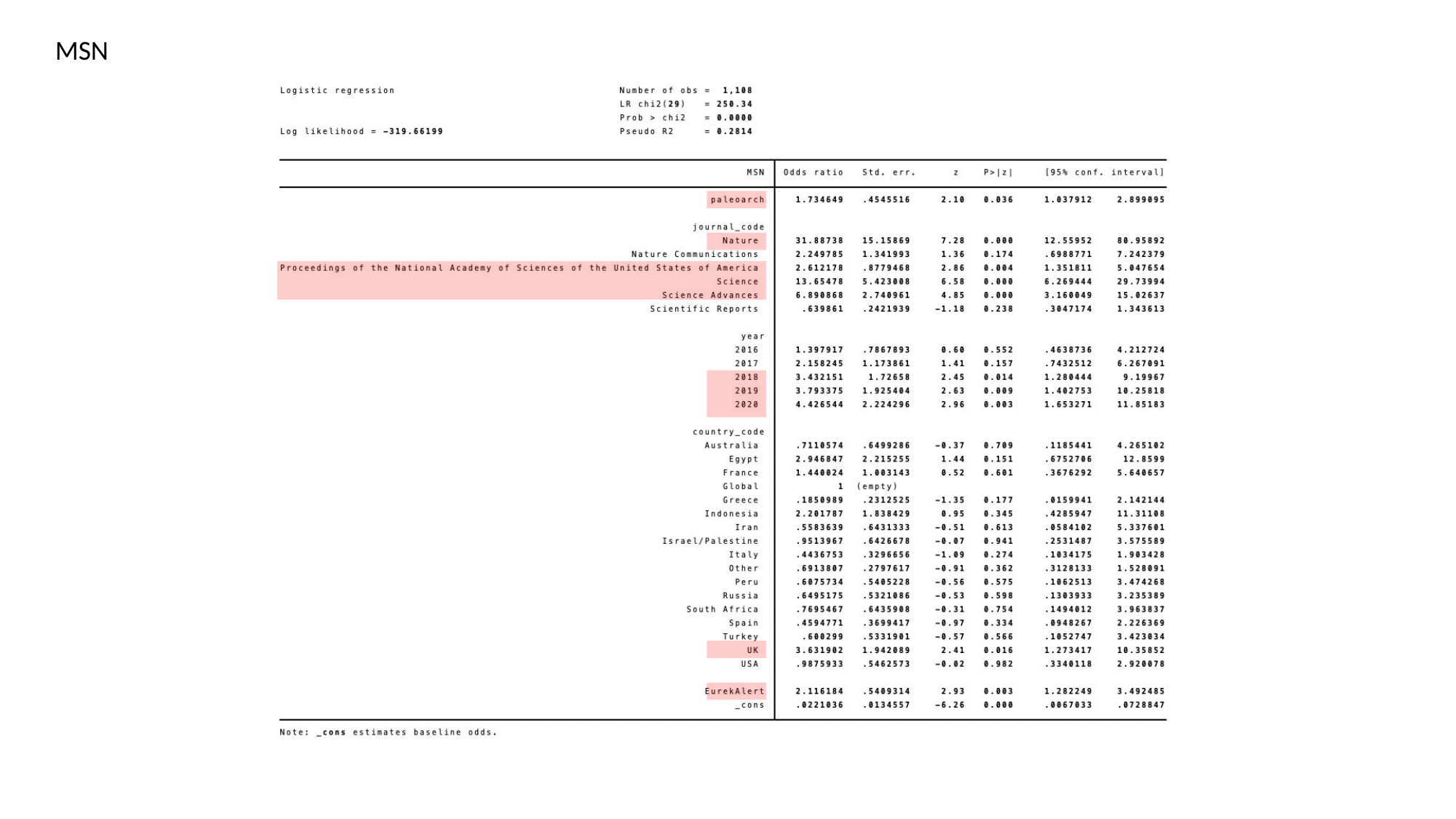

MSN

## Slide 5
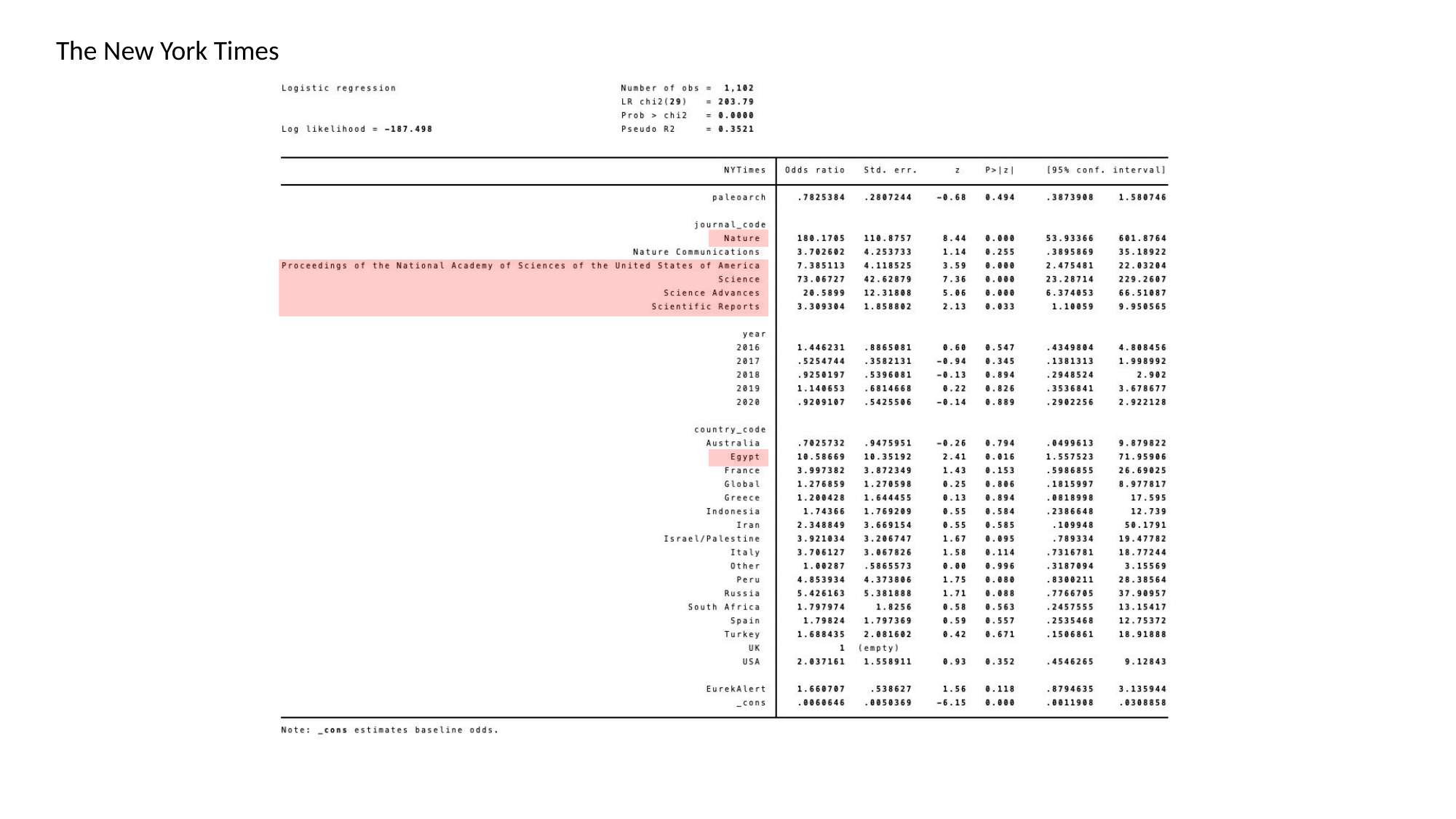

The New York Times

## Slide 6
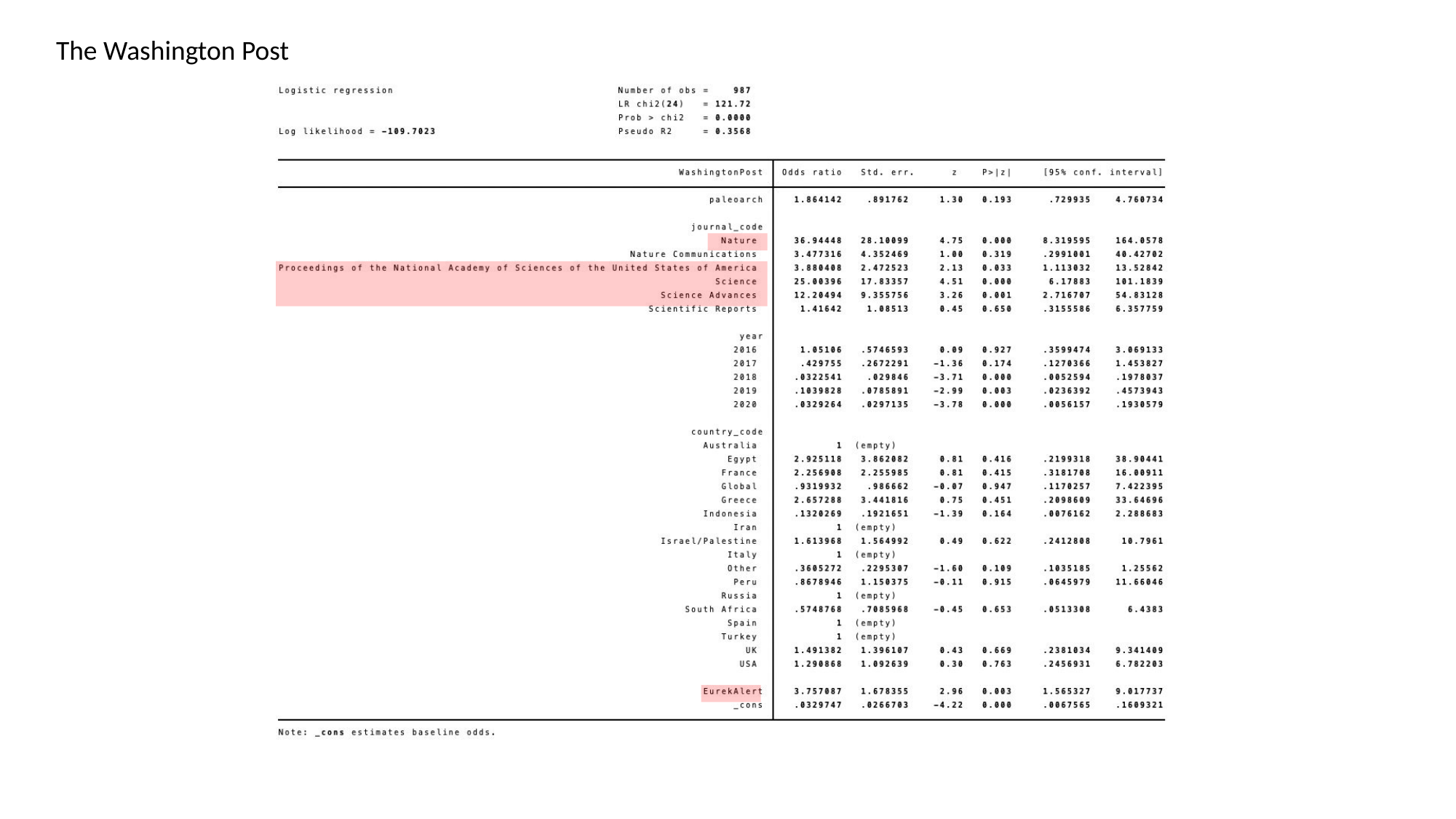

The Washington Post

## Slide 7
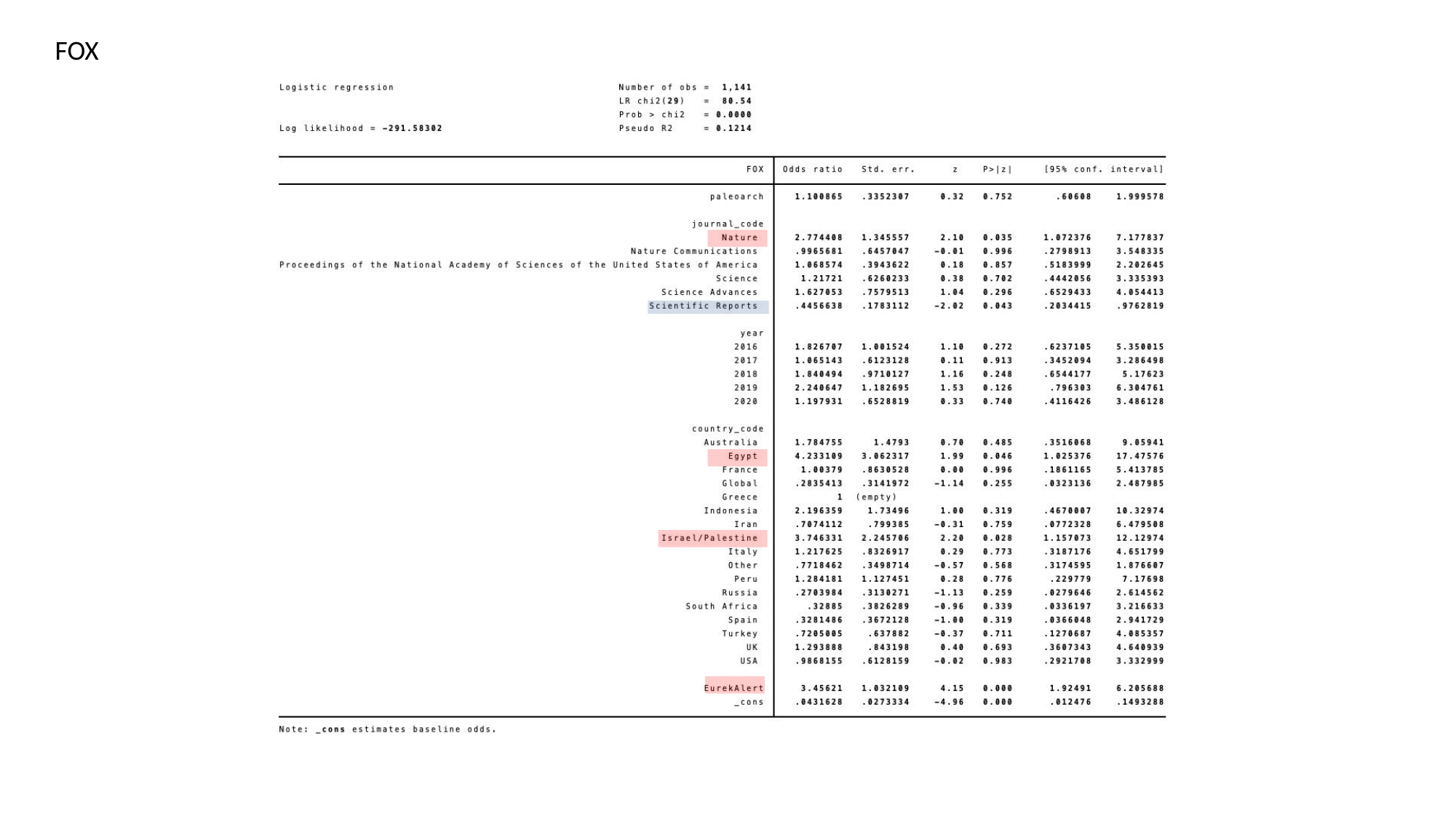

FOX

## Slide 8
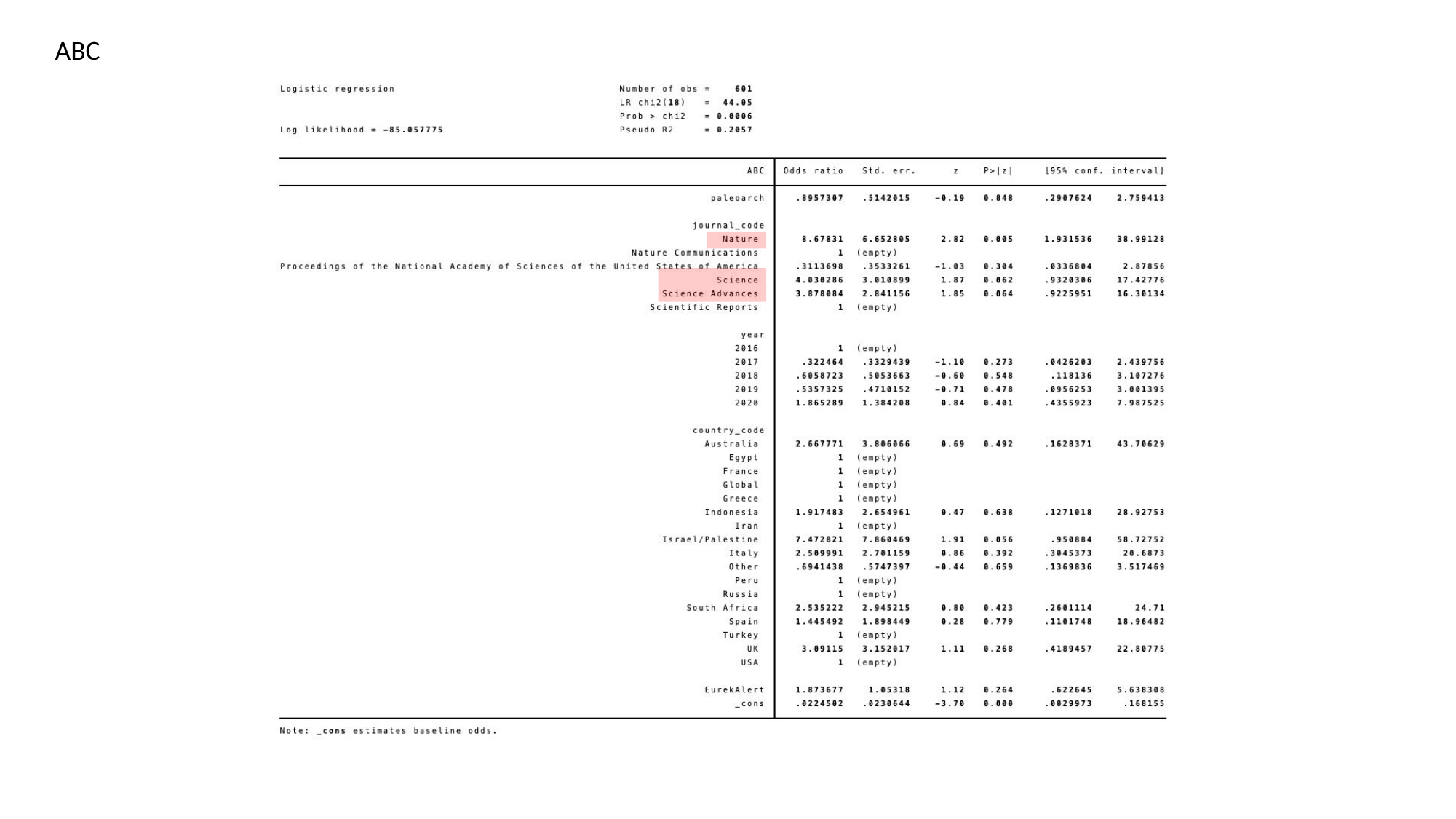

ABC

## Slide 9
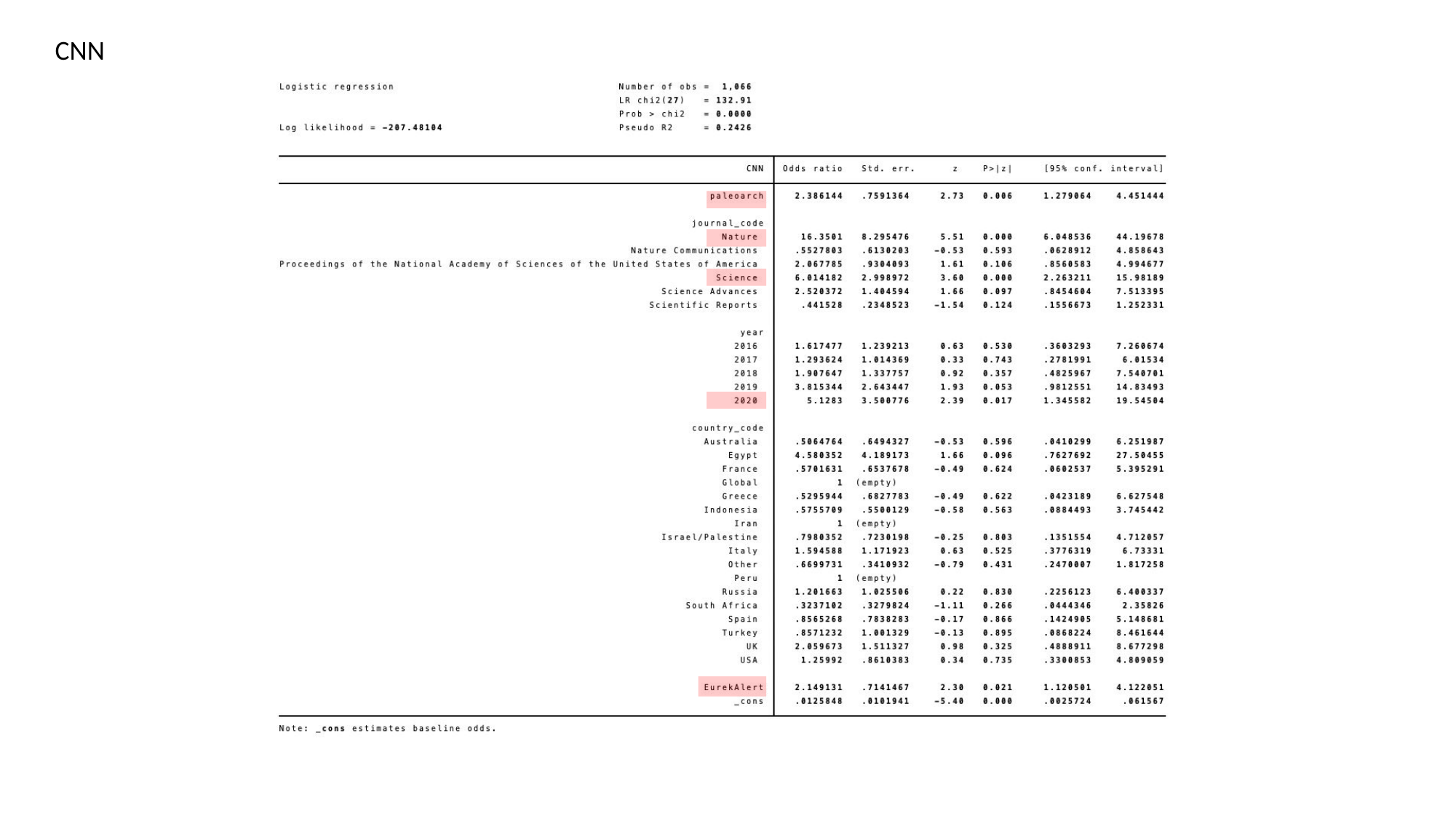

CNN

## Slide 10
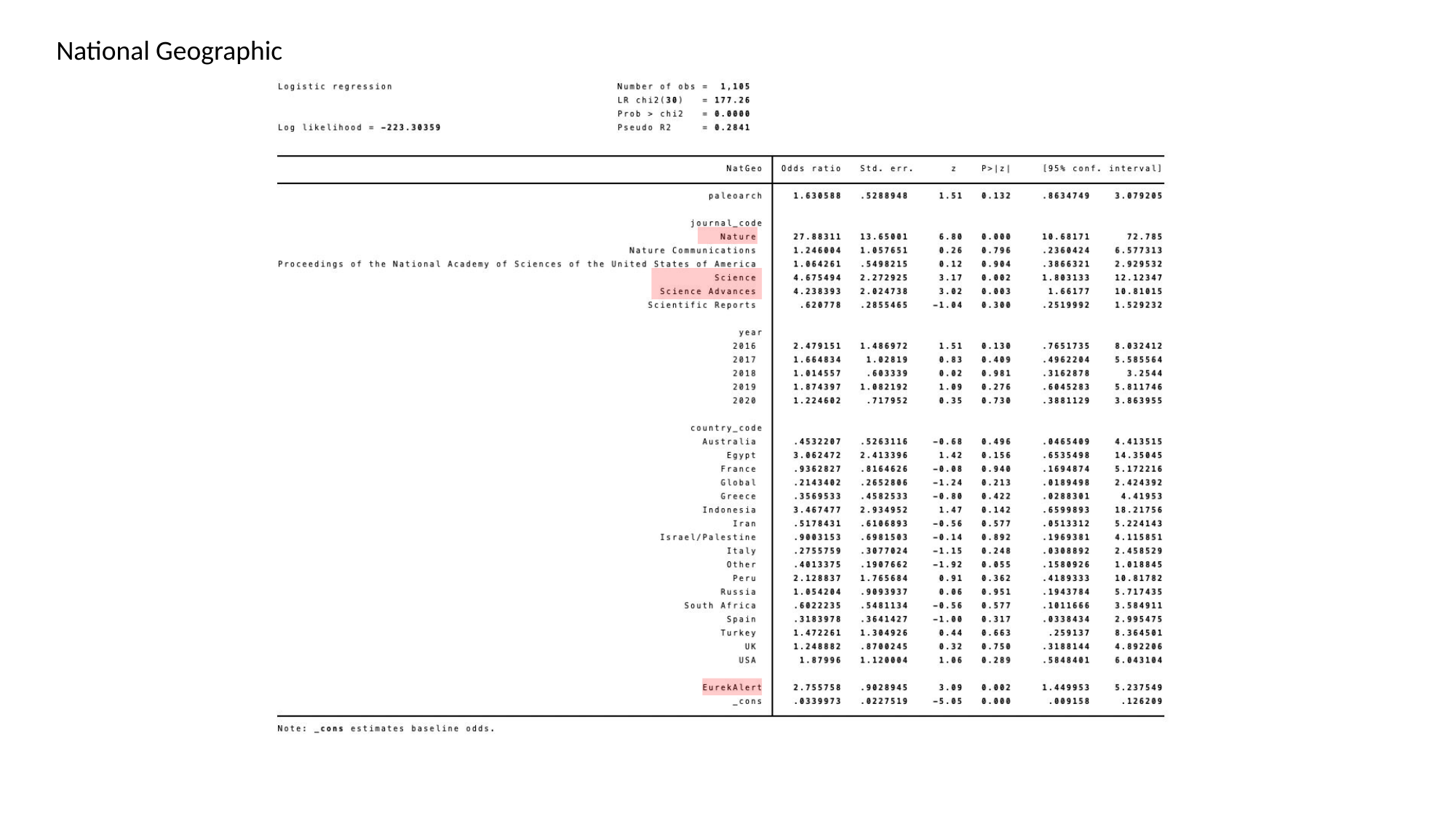

National Geographic

## Slide 11
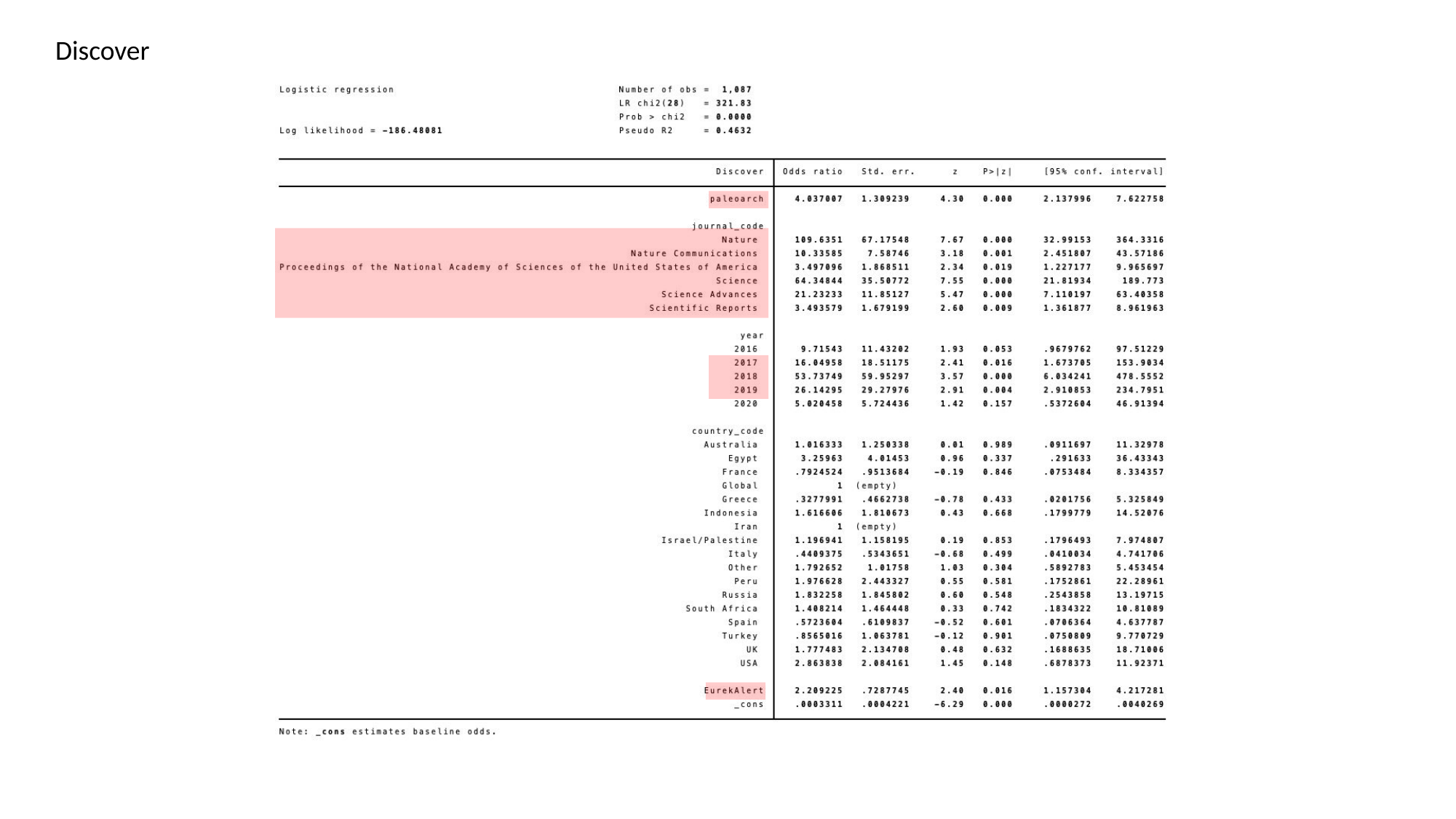

Discover

## Slide 12
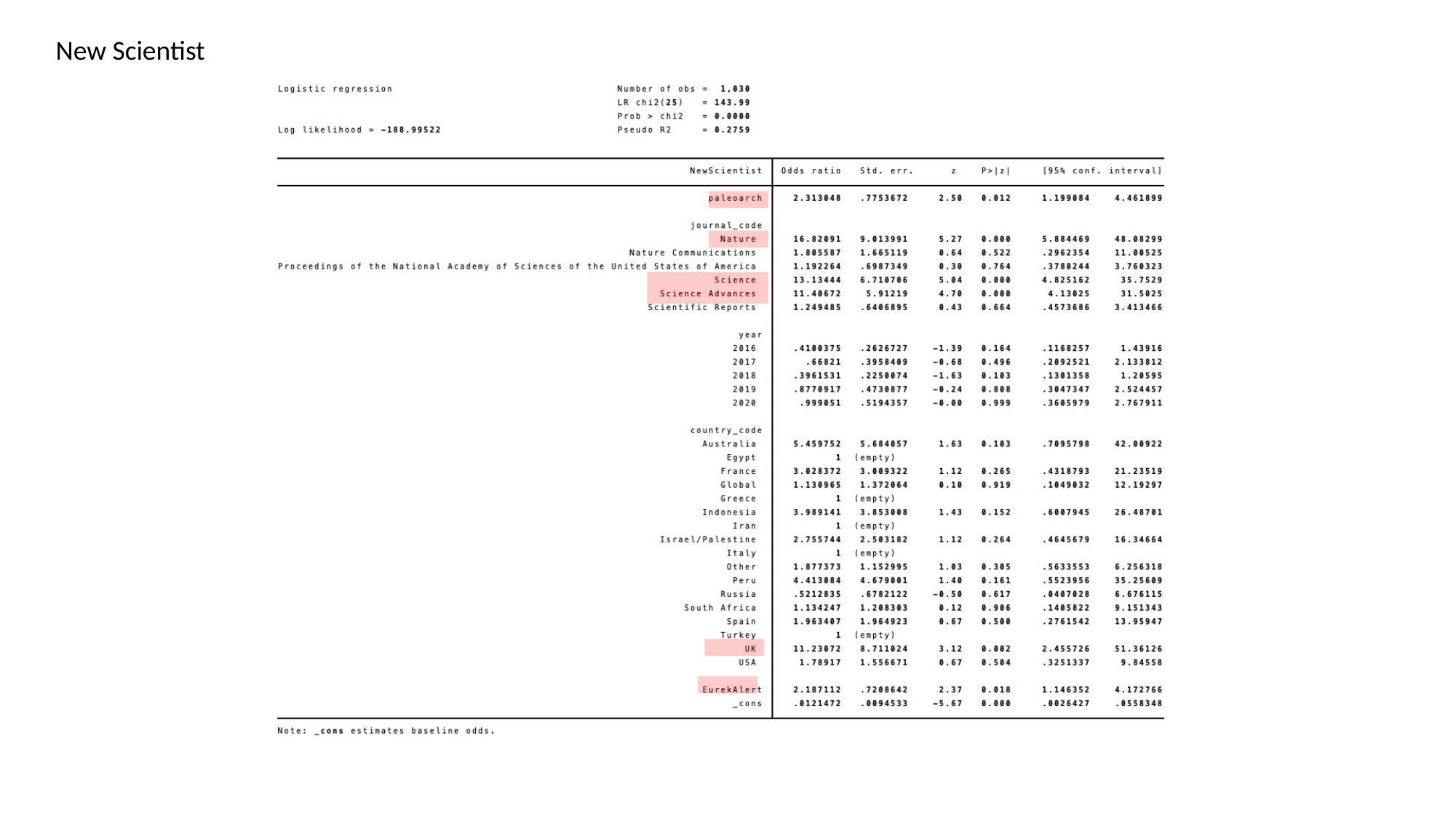

New Scientist

## Slide 13
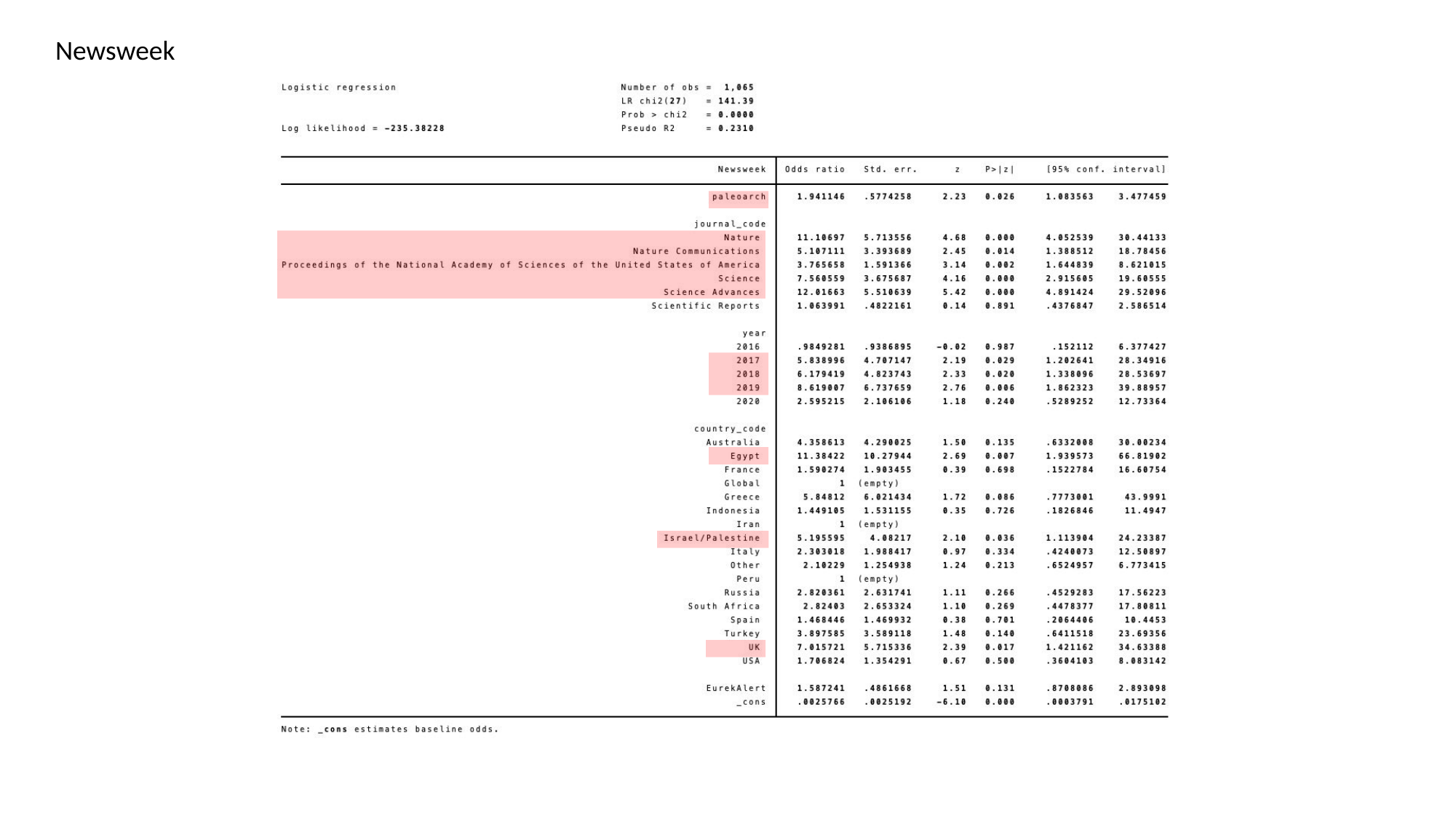

Newsweek

## Slide 14
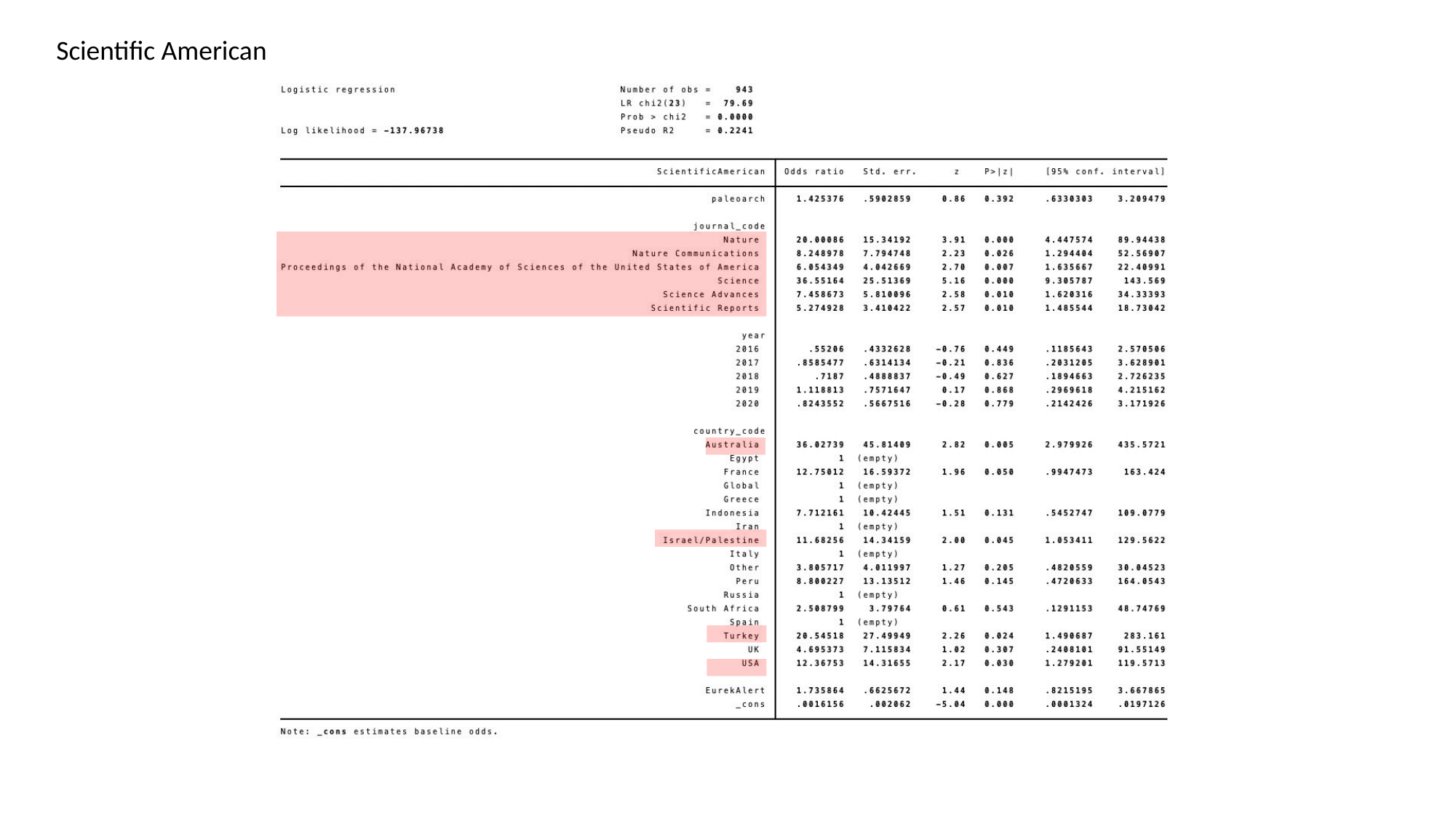

Scientific American

## Slide 15
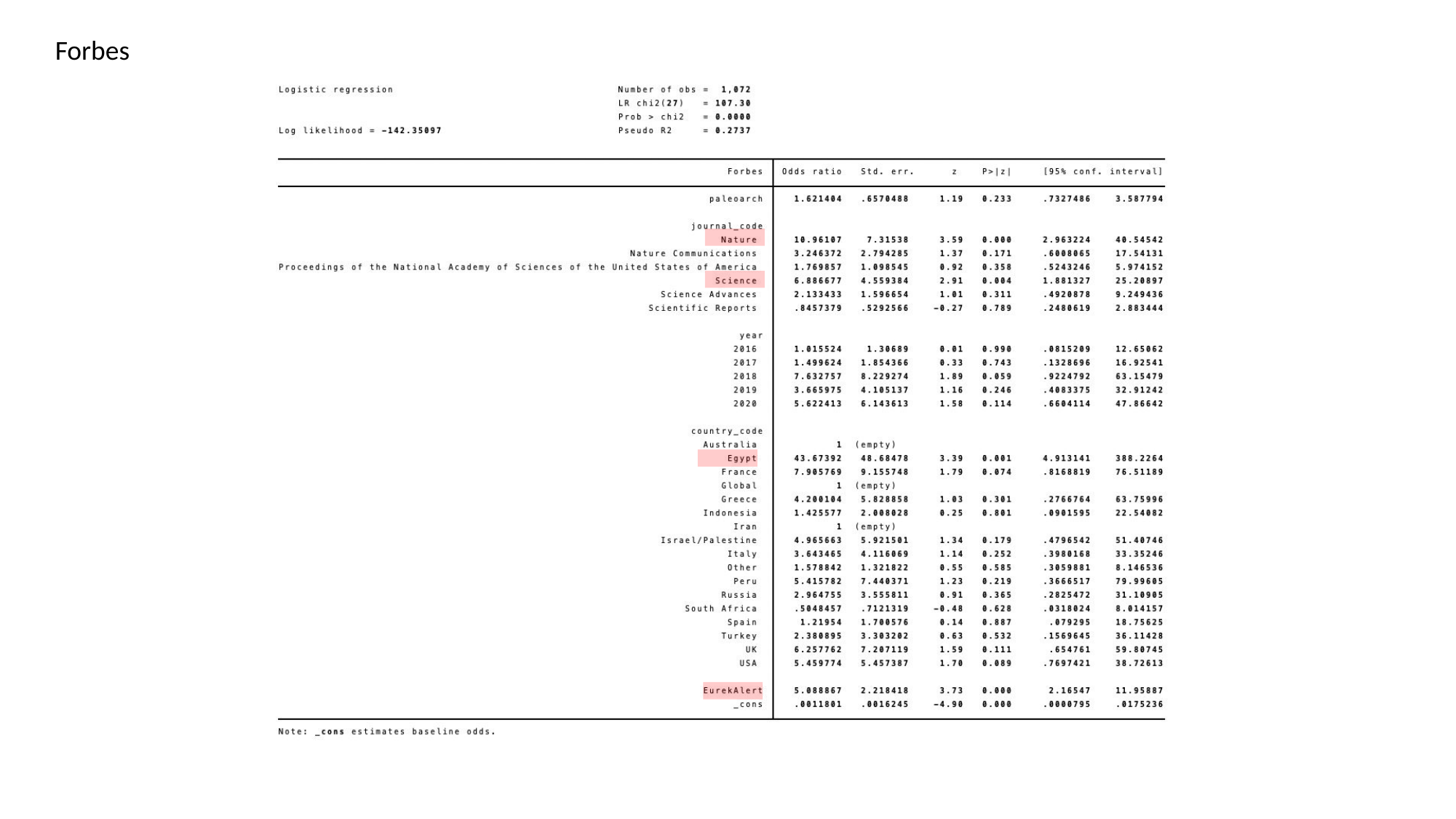

Forbes

## Slide 16
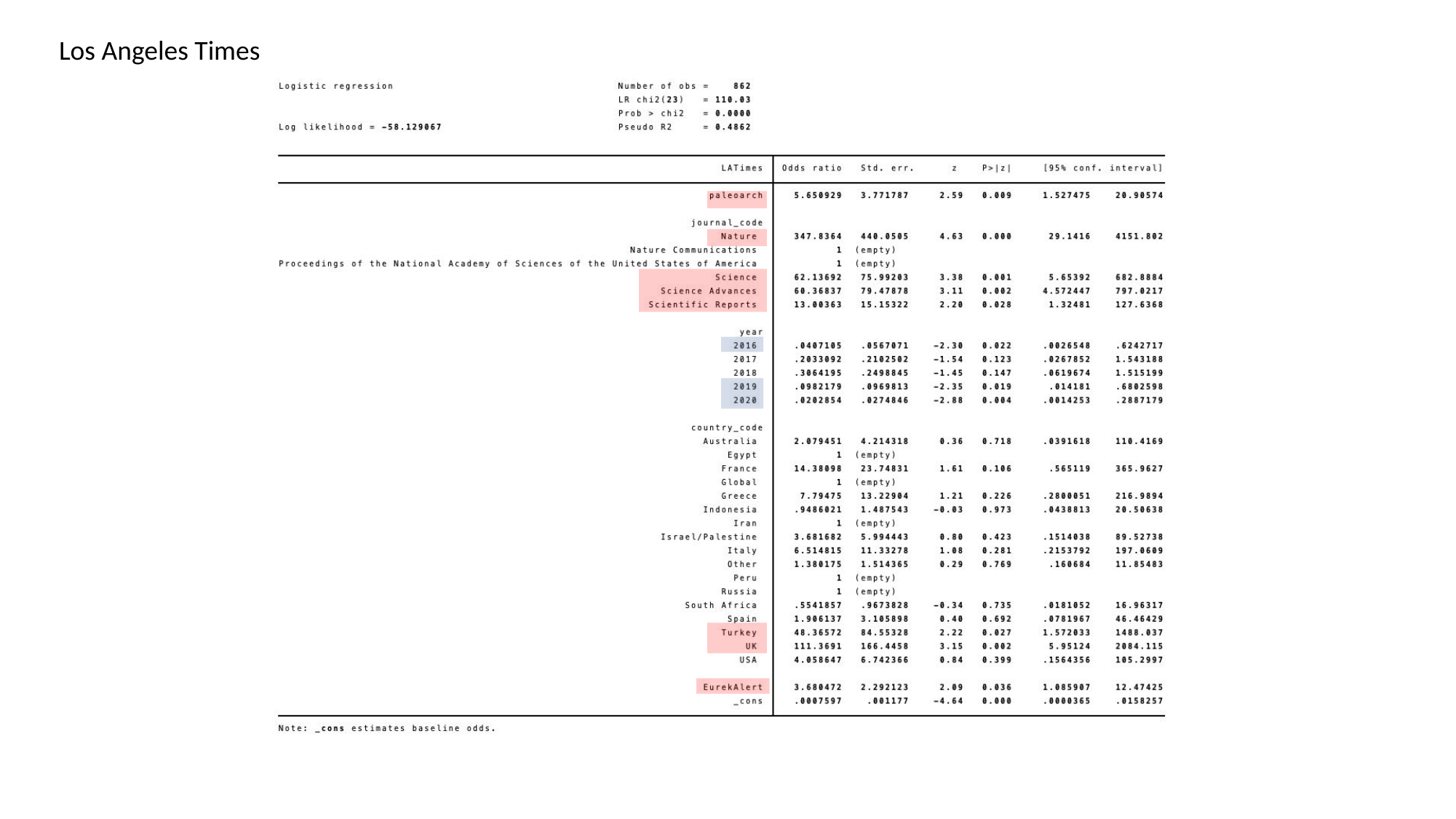

Los Angeles Times

## Slide 17
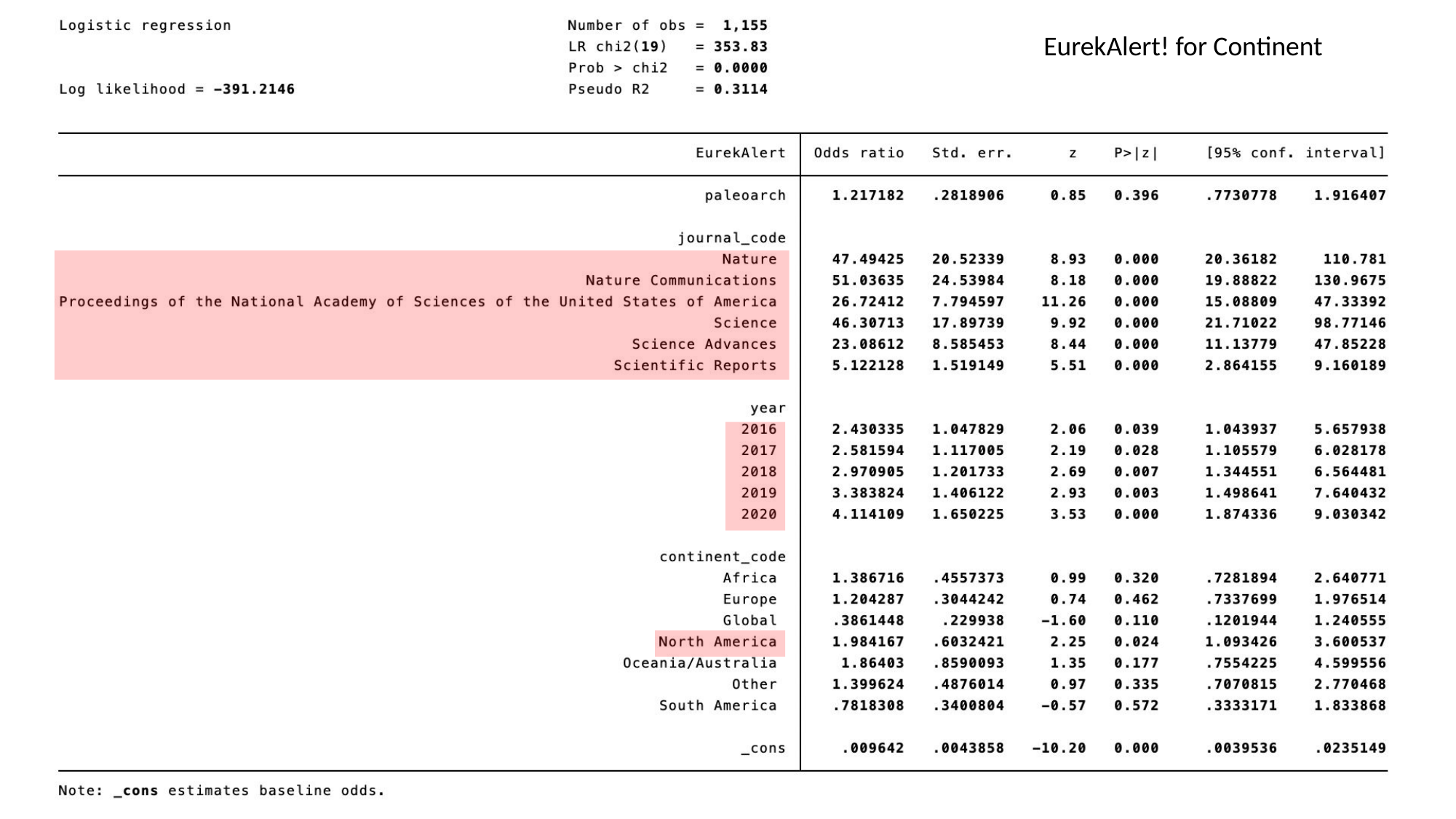

EurekAlert! for Continent

## Slide 18
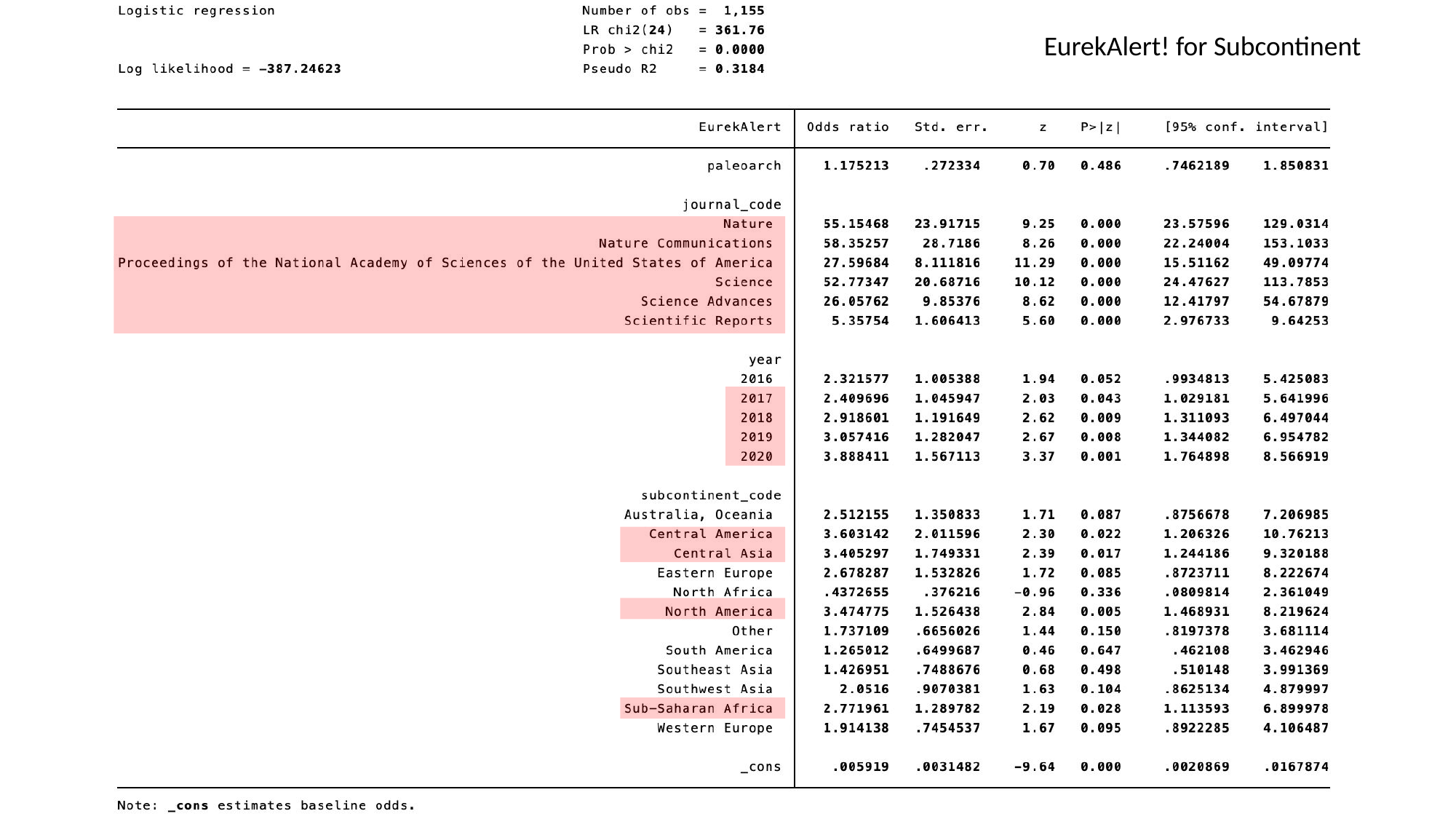

EurekAlert! for Subcontinent
#

## Slide 19
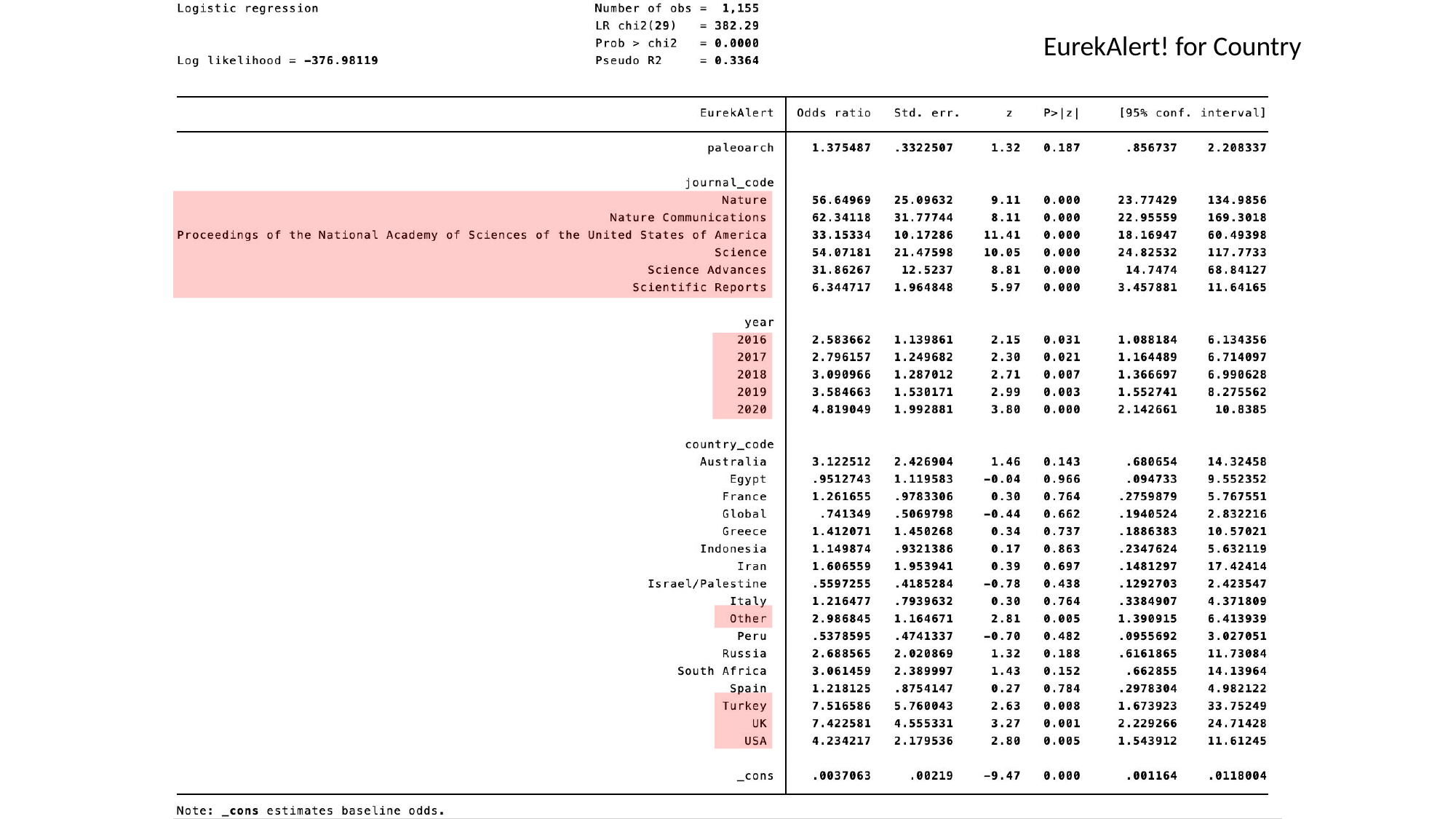

EurekAlert! for Country
#
