# Supplementary material for: Regional disparities in US media coverage of archaeology research
Source: Sci Adv. 2025 Jul 2;11(27):eadt5435. doi: 10.1126/sciadv.adt5435 (PMC12219491; doi:10.1126/sciadv.adt5435)
Supplement: Supplementary file 2 — Data S1 to S6 [file sciadv.adt5435_data_s1_to_s6.zip › adt5435_data_s5.pptx]

## Slide 1
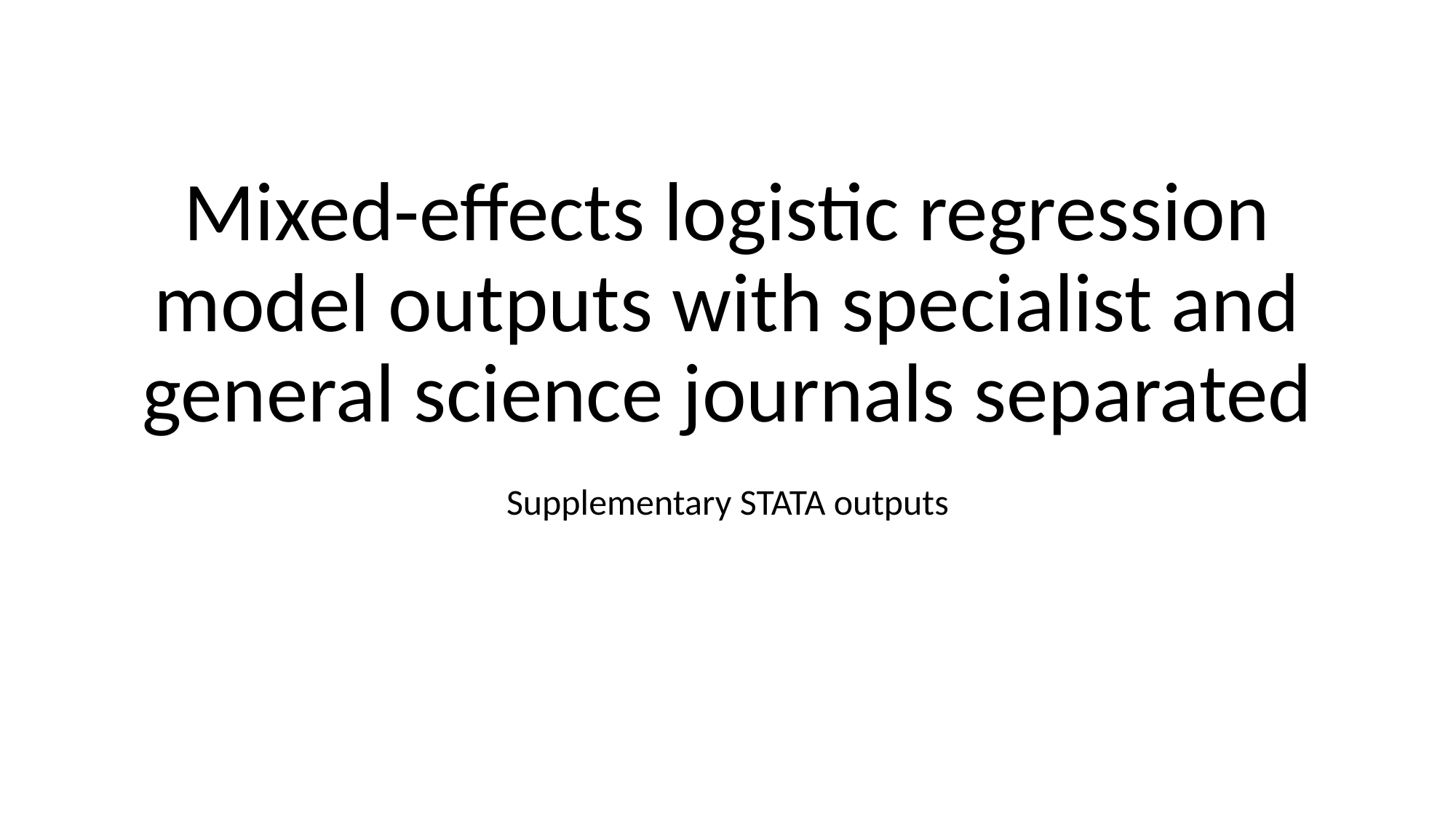

# Mixed-effects logistic regression model outputs with specialist and general science journals separated
Supplementary STATA outputs

## Slide 2
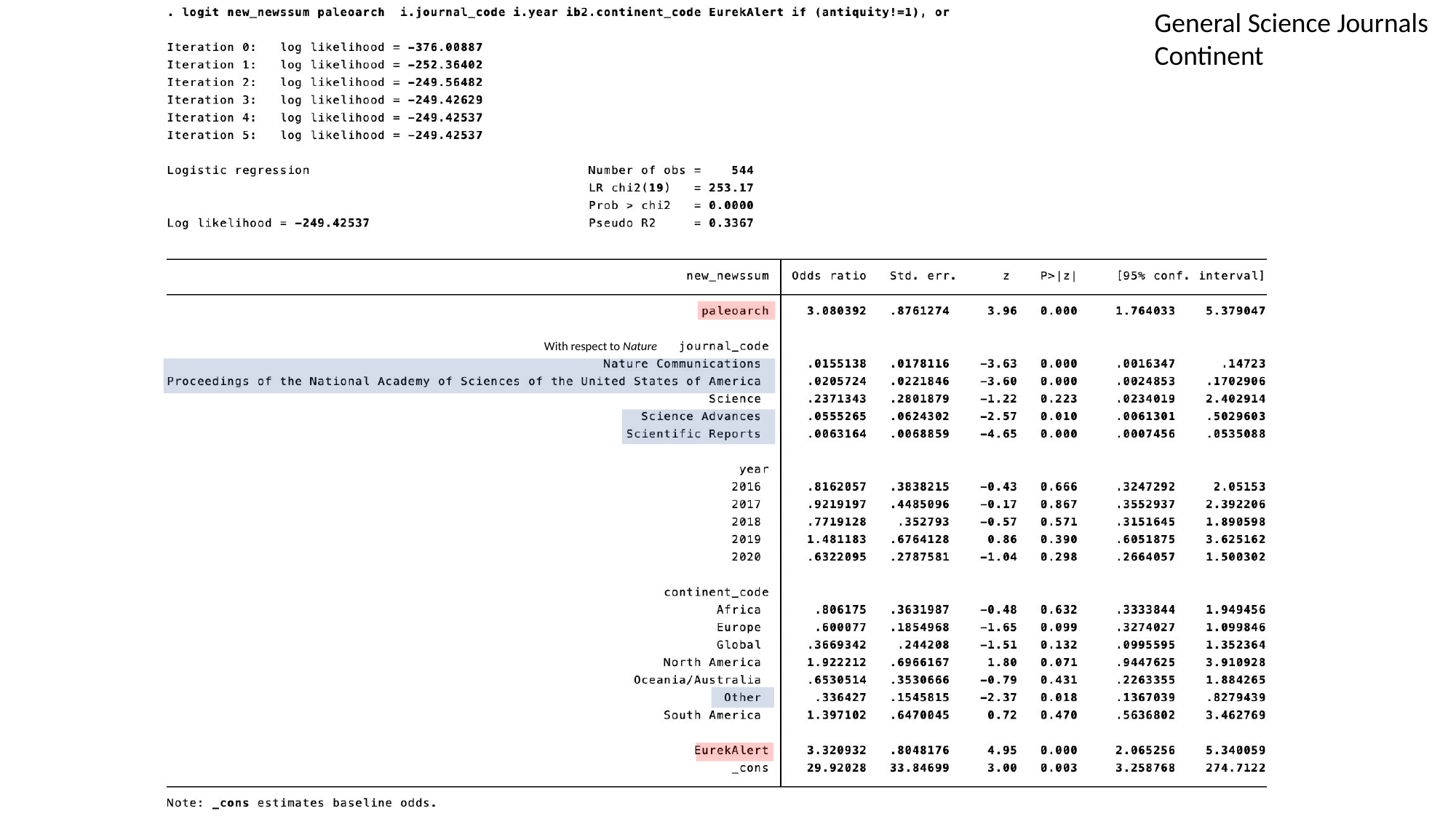

General Science Journals
Continent
With respect to Nature

## Slide 3
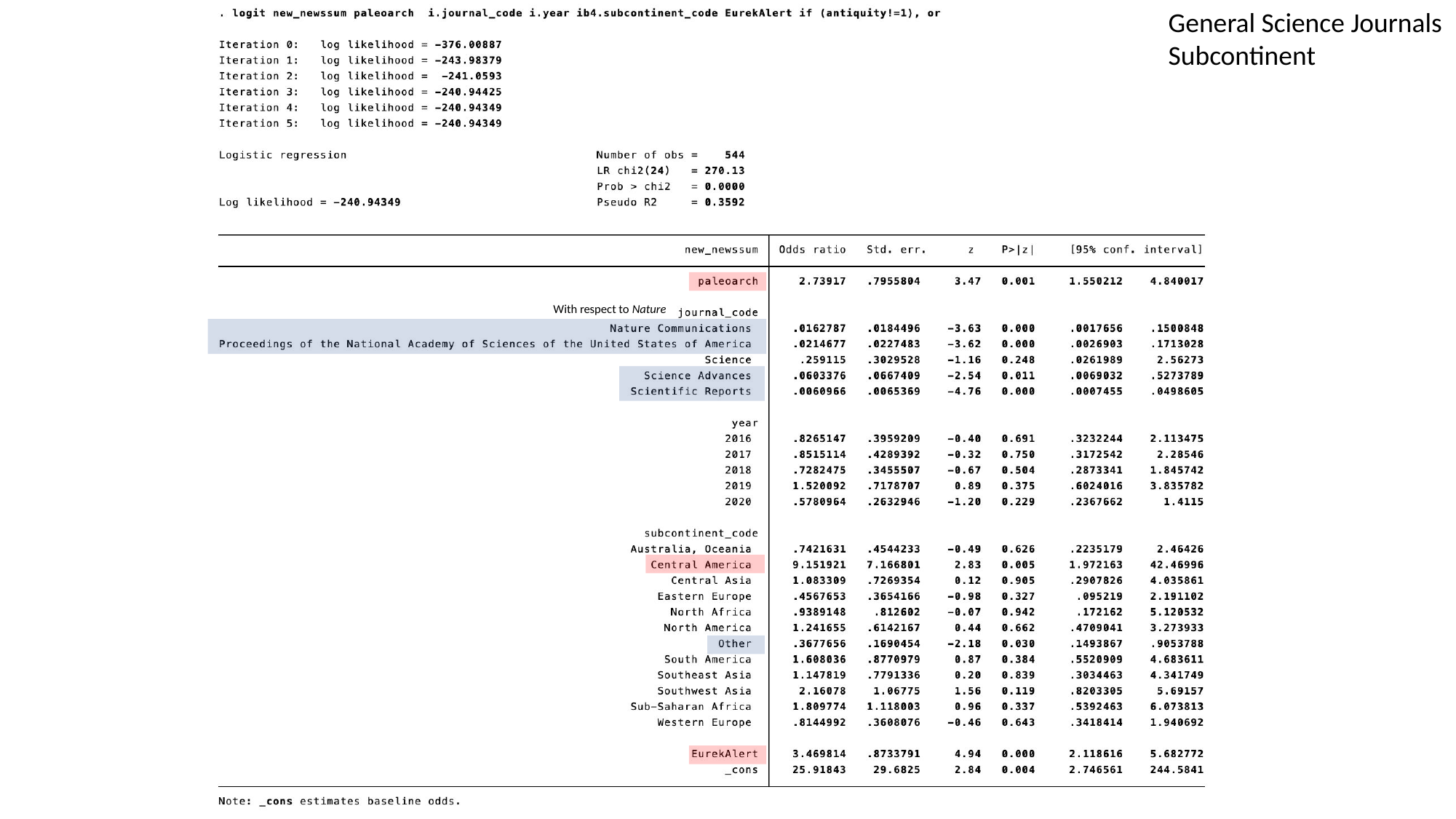

General Science Journals
Subcontinent
With respect to Nature

## Slide 4
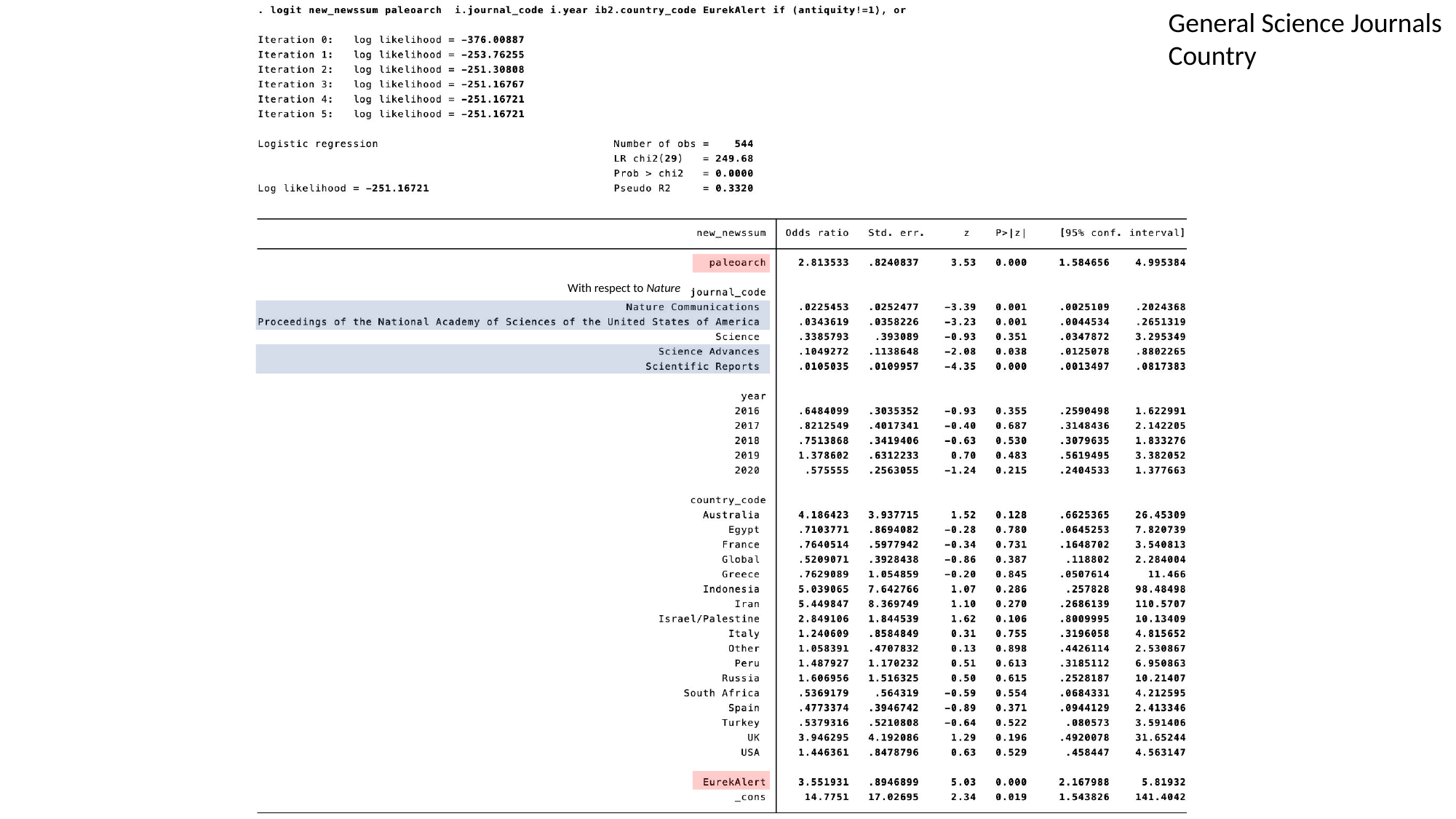

General Science Journals
Country
With respect to Nature

## Slide 5
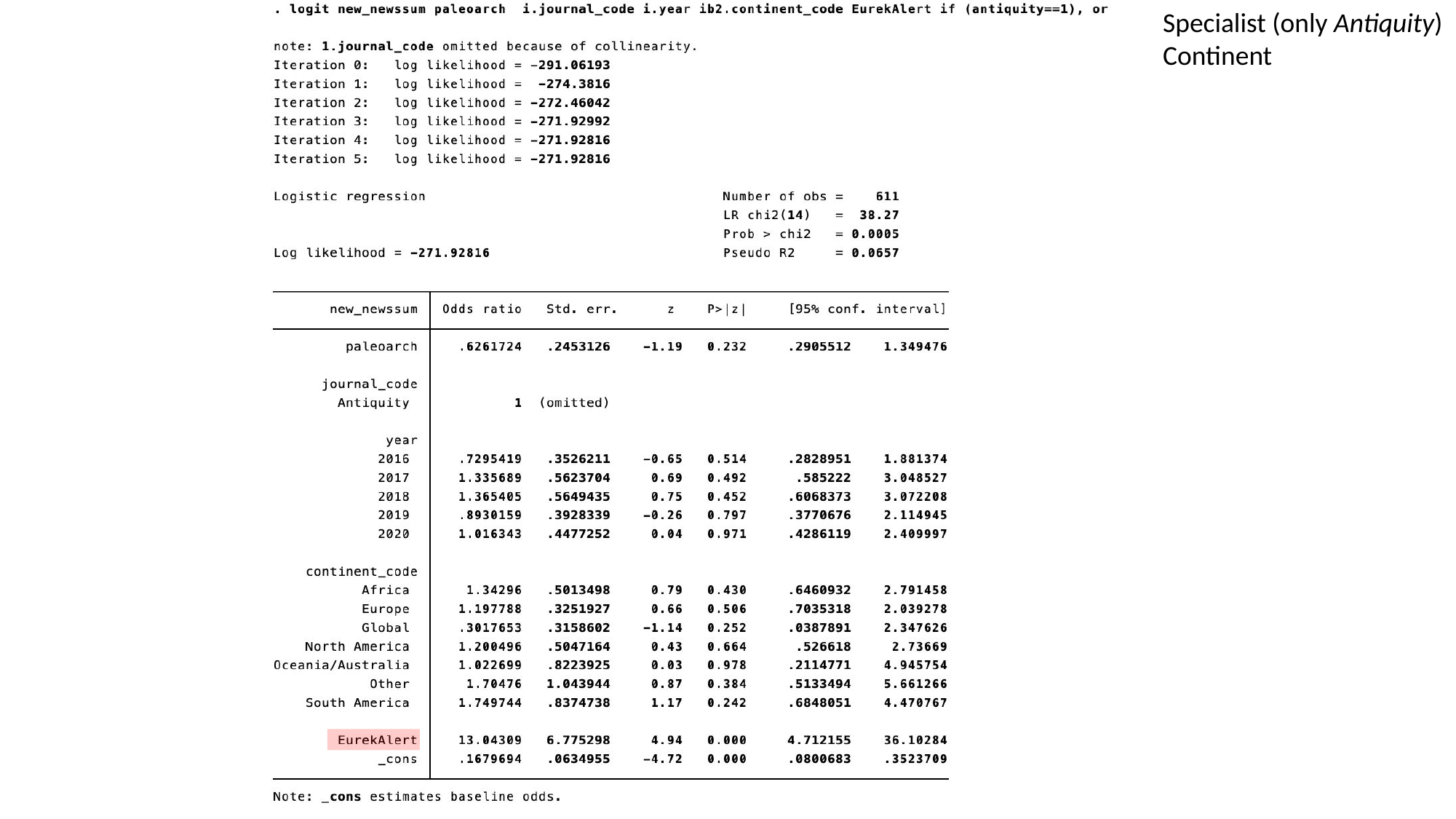

Specialist (only Antiquity)
Continent

## Slide 6
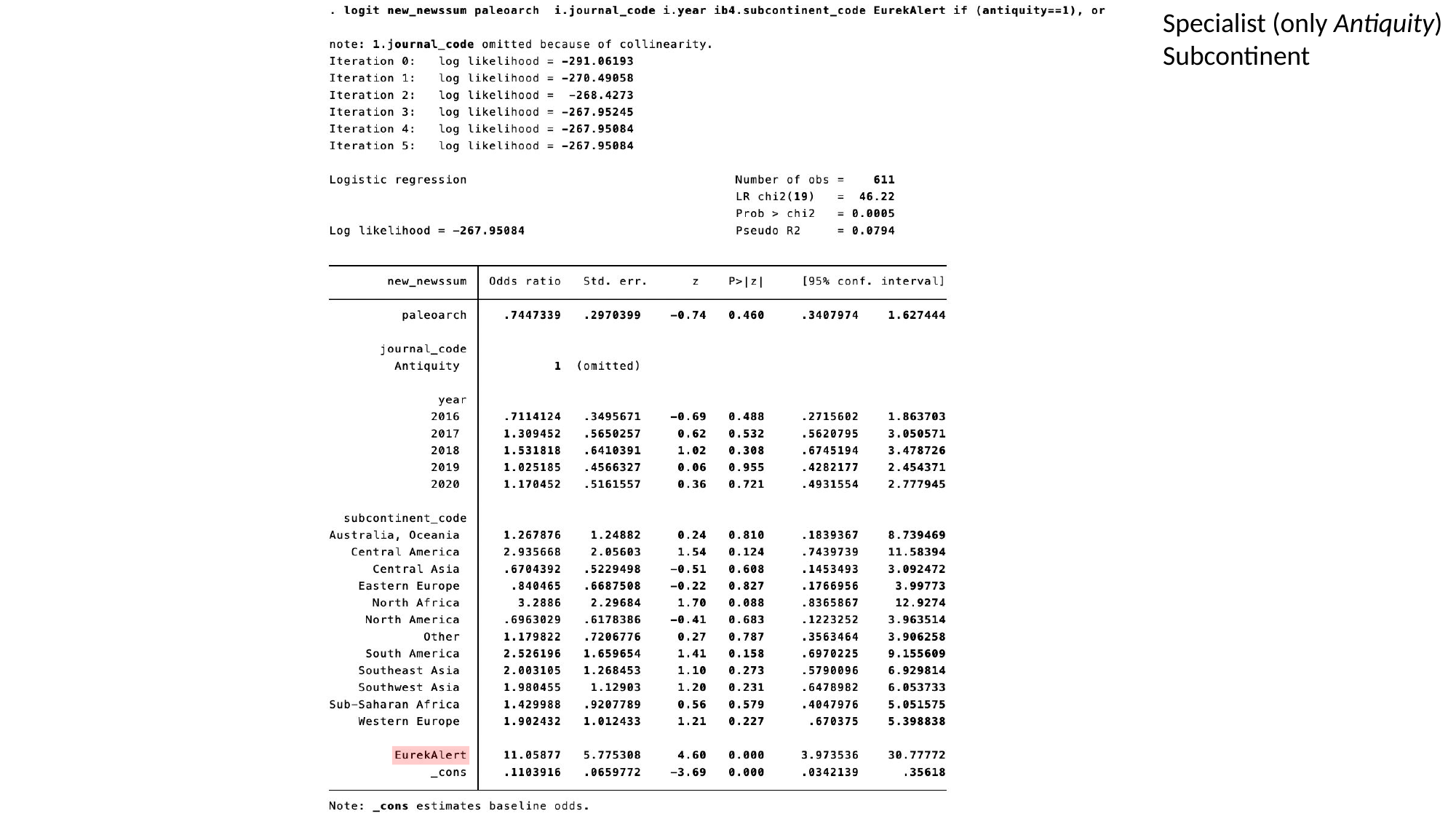

Specialist (only Antiquity)
Subcontinent

## Slide 7
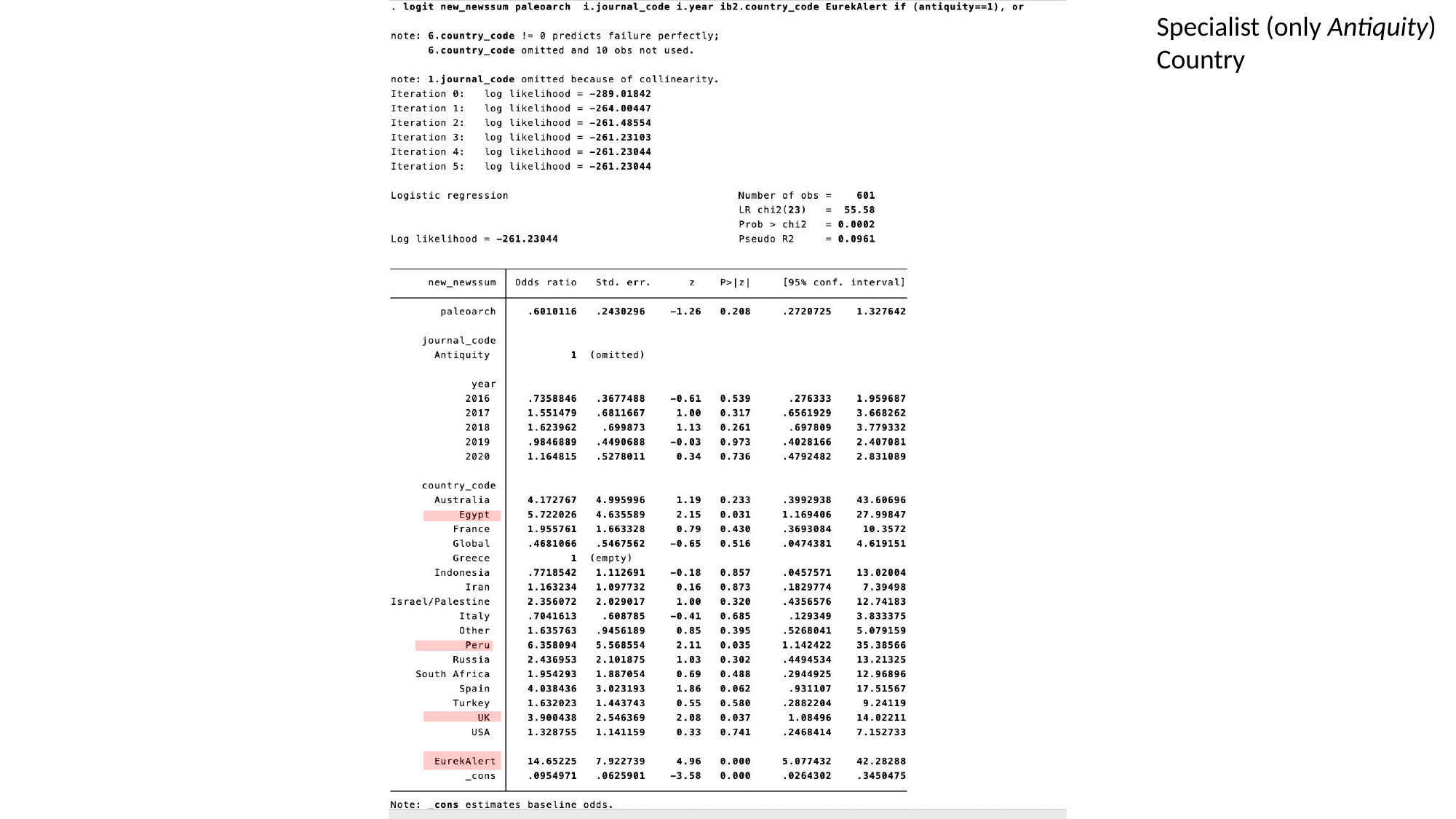

Specialist (only Antiquity)
Country
