# Supplementary material for: Regional disparities in US media coverage of archaeology research
Source: Sci Adv. 2025 Jul 2;11(27):eadt5435. doi: 10.1126/sciadv.adt5435 (PMC12219491; doi:10.1126/sciadv.adt5435)
Supplement: Supplementary file 2 — Data S1 to S6 [file sciadv.adt5435_data_s1_to_s6.zip › adt5435_data_s6.pptx]

## Slide 1
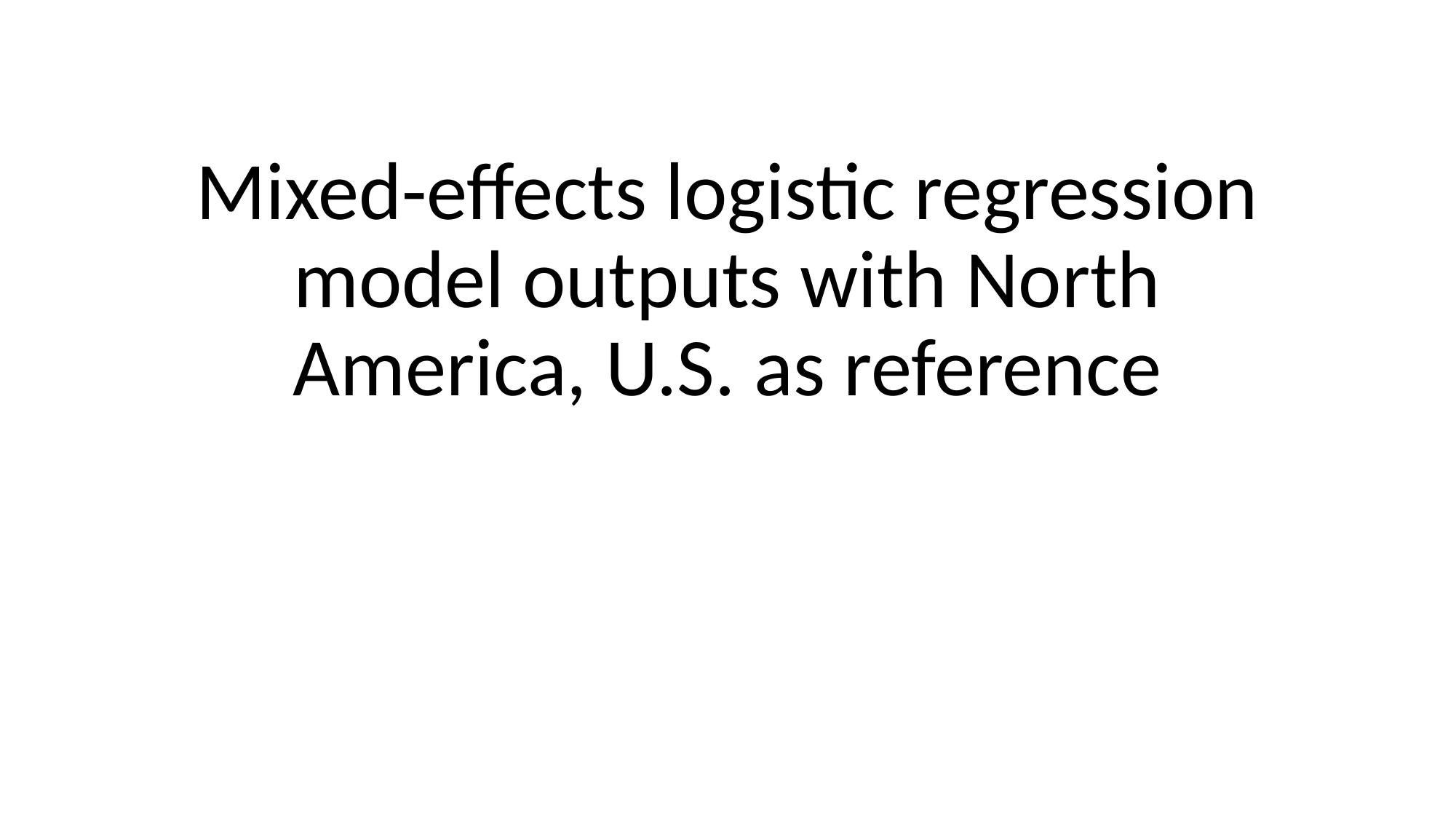

# Mixed-effects logistic regression model outputs with North America, U.S. as reference

## Slide 2
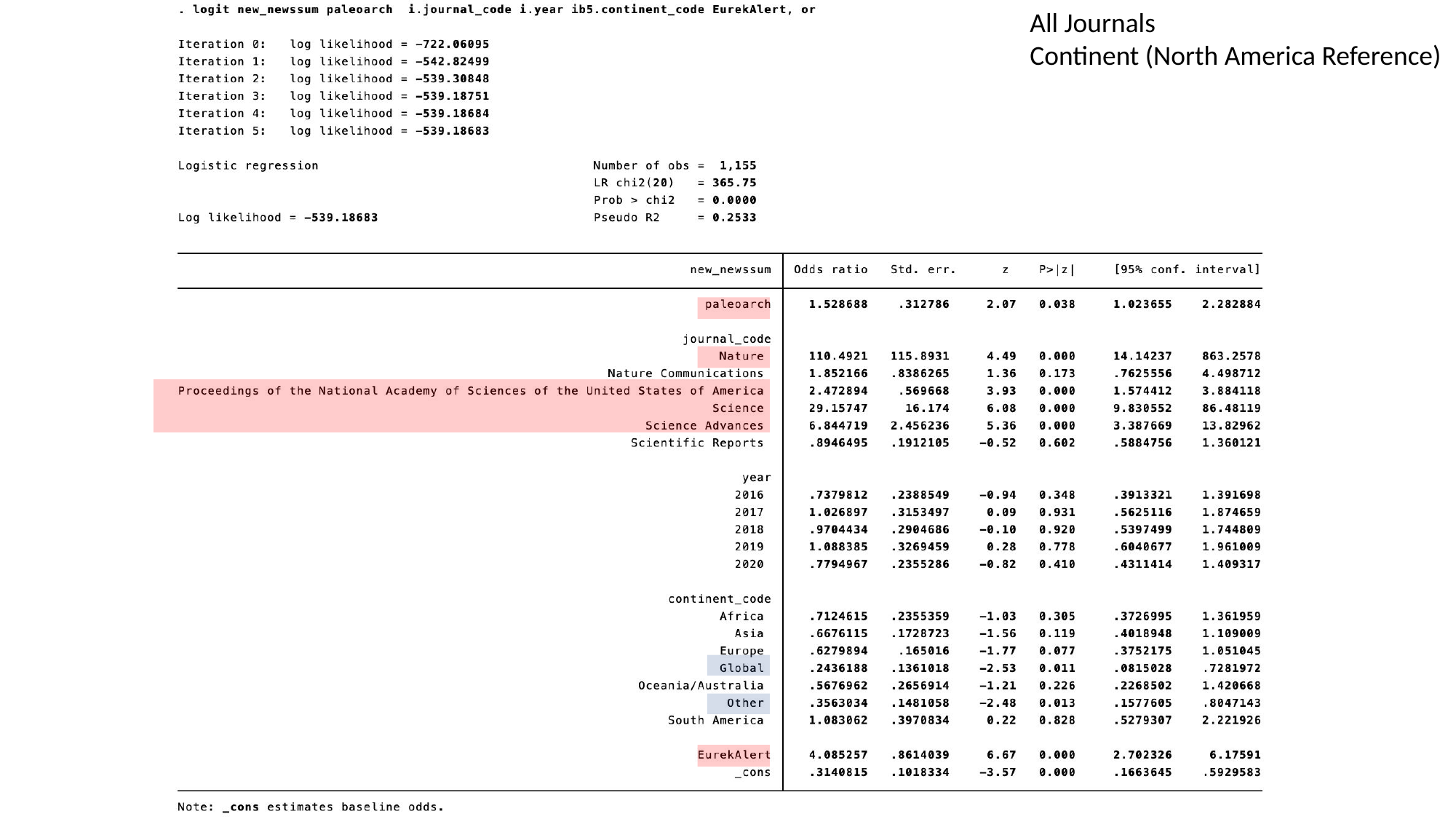

All Journals
Continent (North America Reference)

## Slide 3
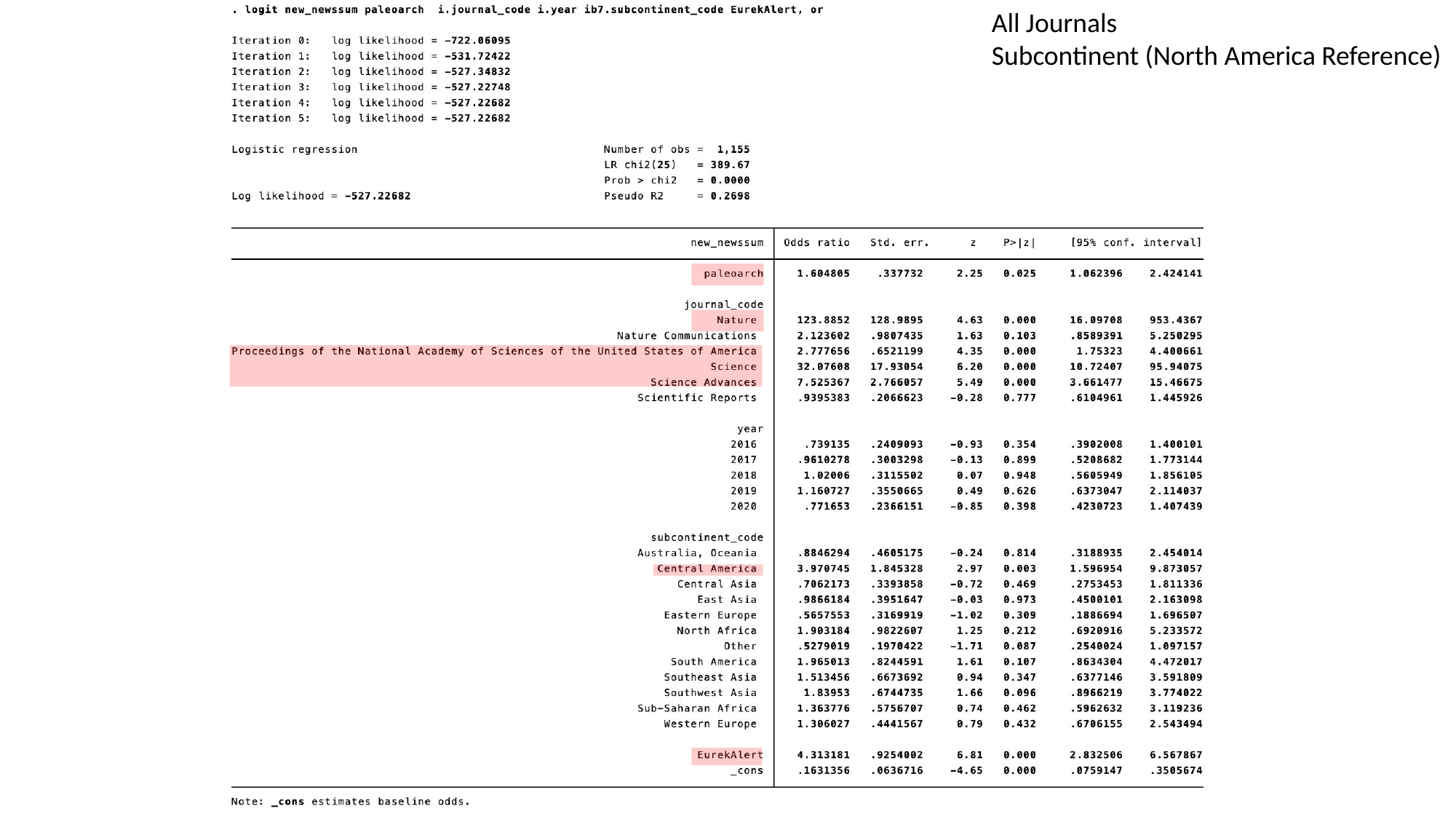

All Journals
Subcontinent (North America Reference)

## Slide 4
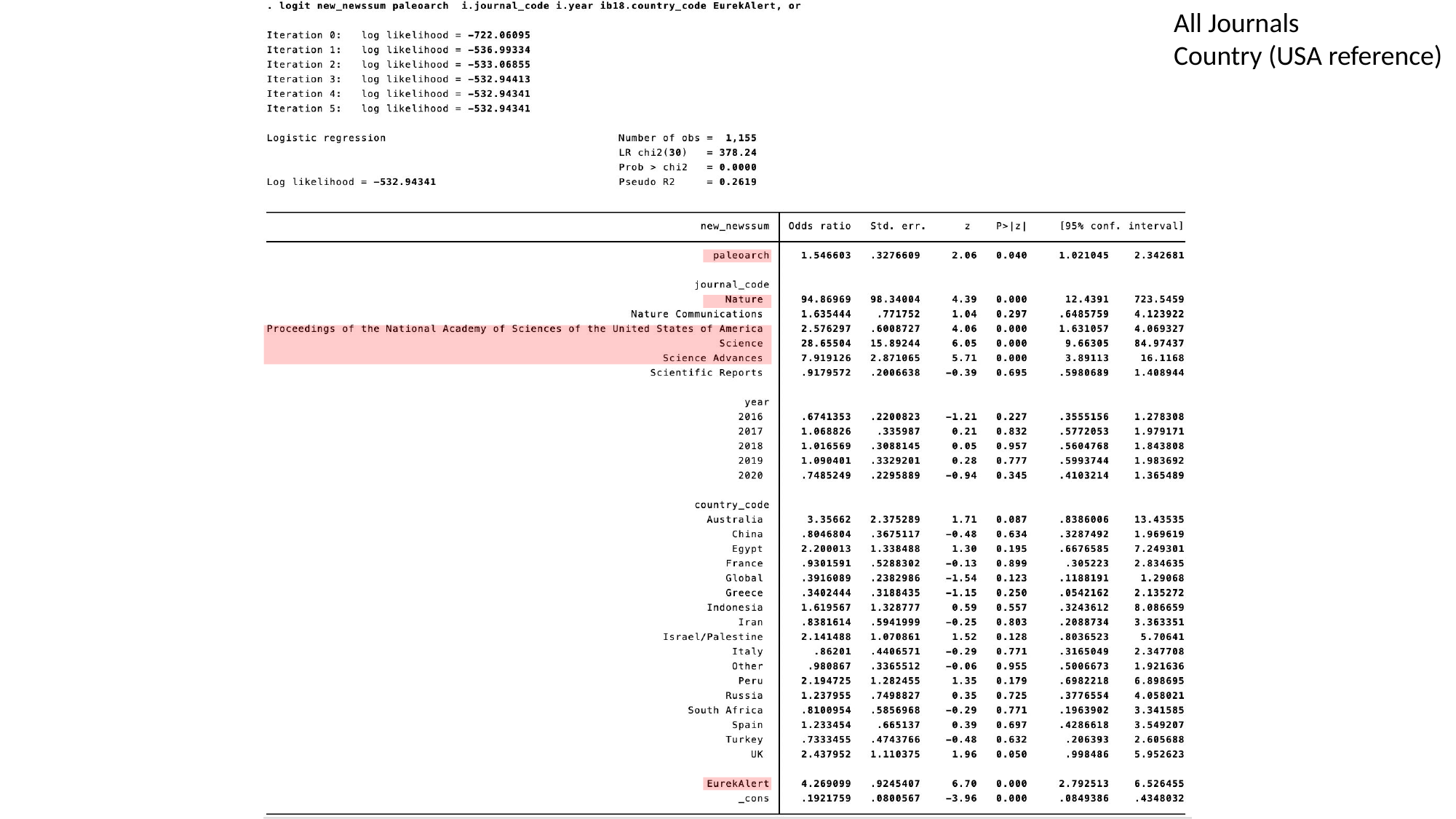

All Journals
Country (USA reference)

## Slide 5
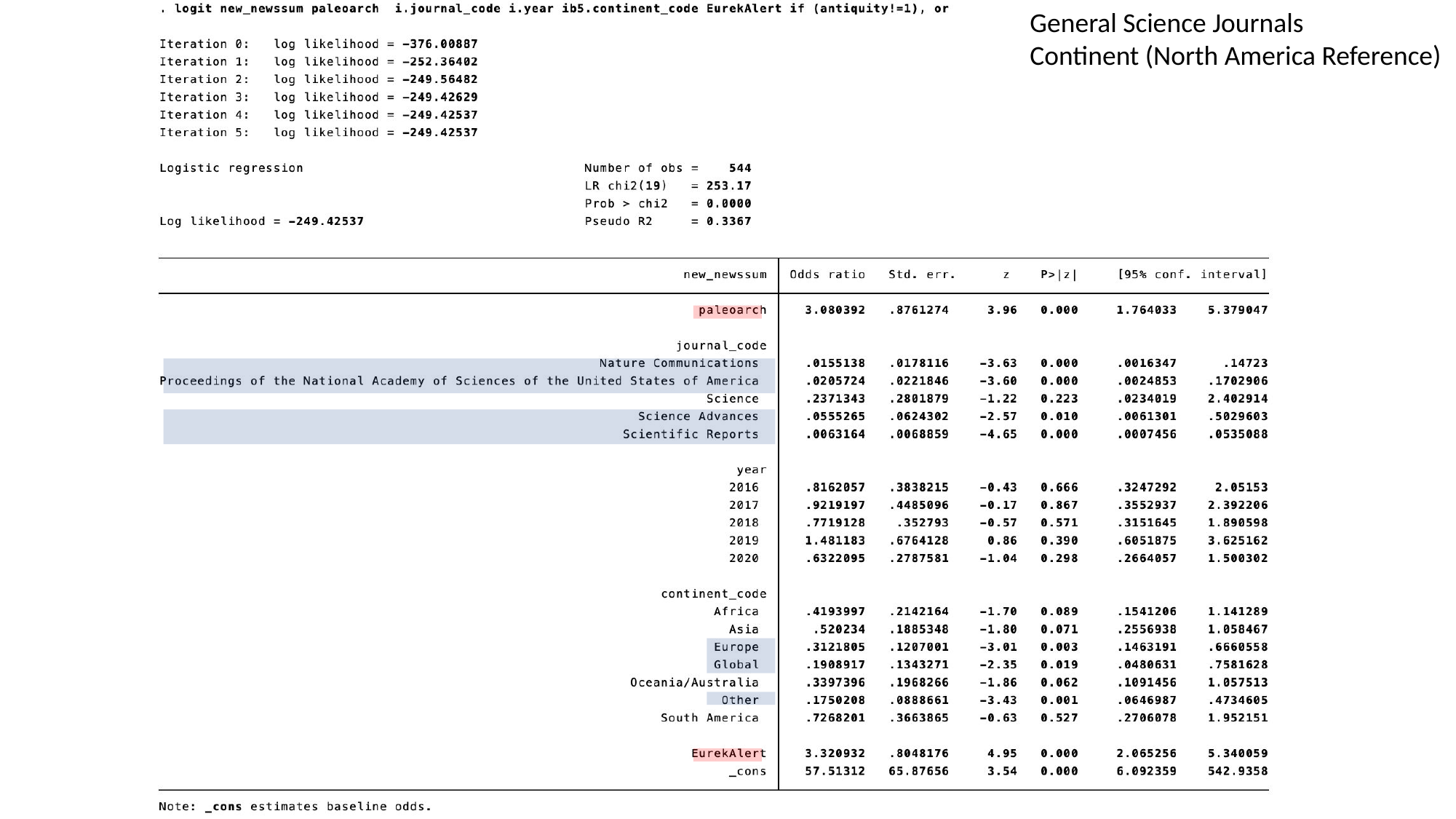

General Science Journals
Continent (North America Reference)

## Slide 6
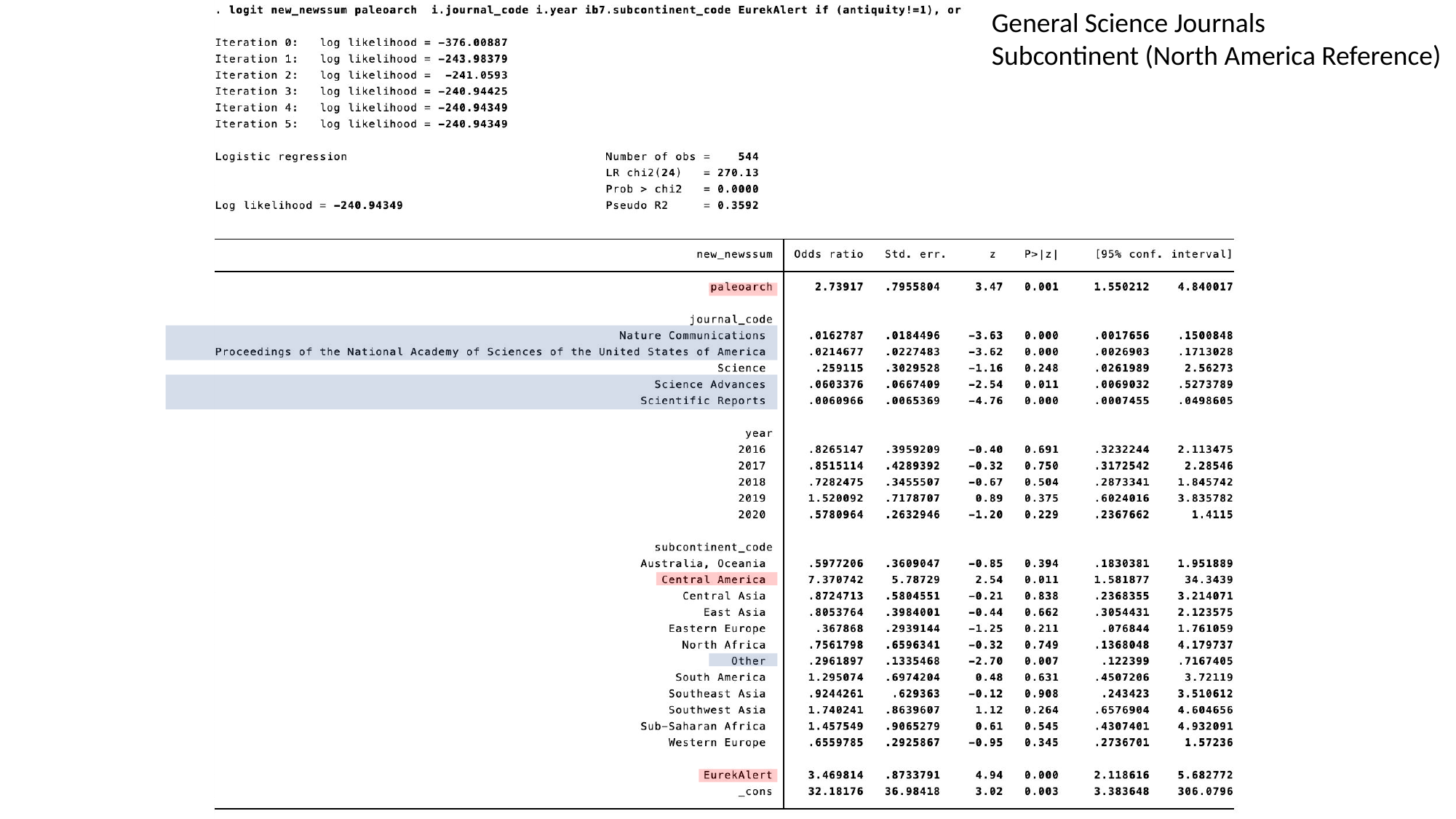

General Science Journals
Subcontinent (North America Reference)

## Slide 7
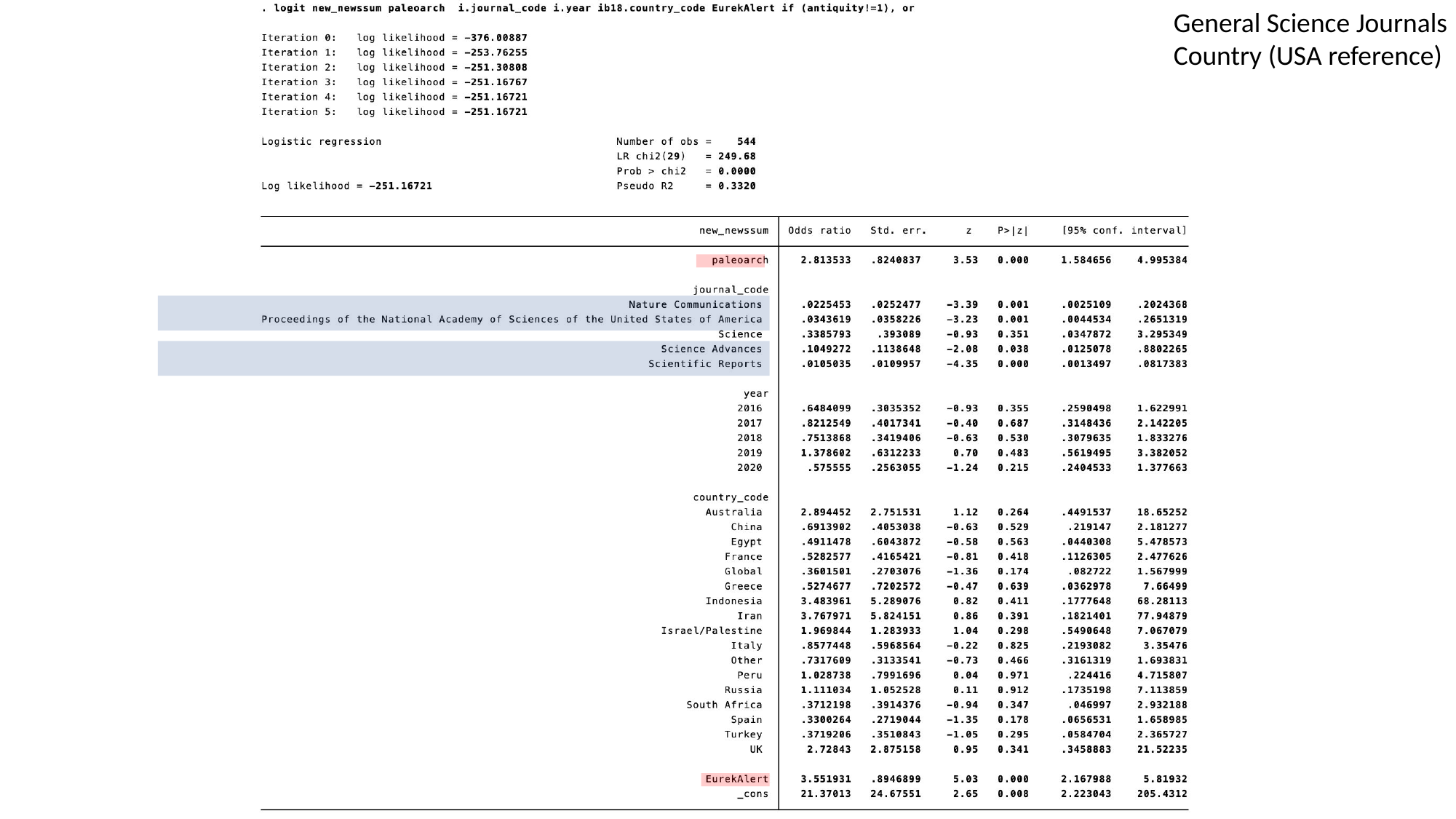

General Science Journals
Country (USA reference)

## Slide 8
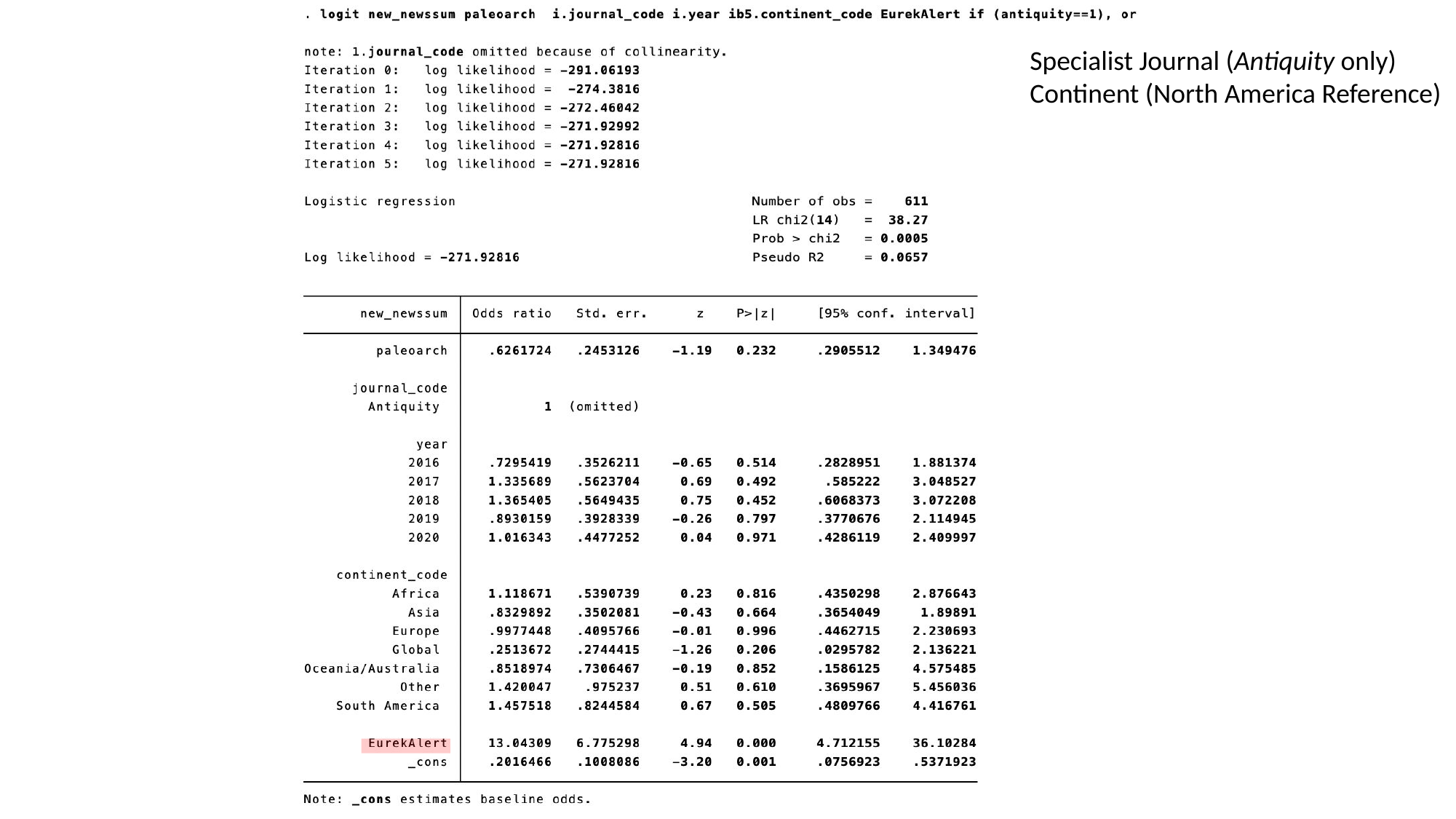

Specialist Journal (Antiquity only)
Continent (North America Reference)

## Slide 9
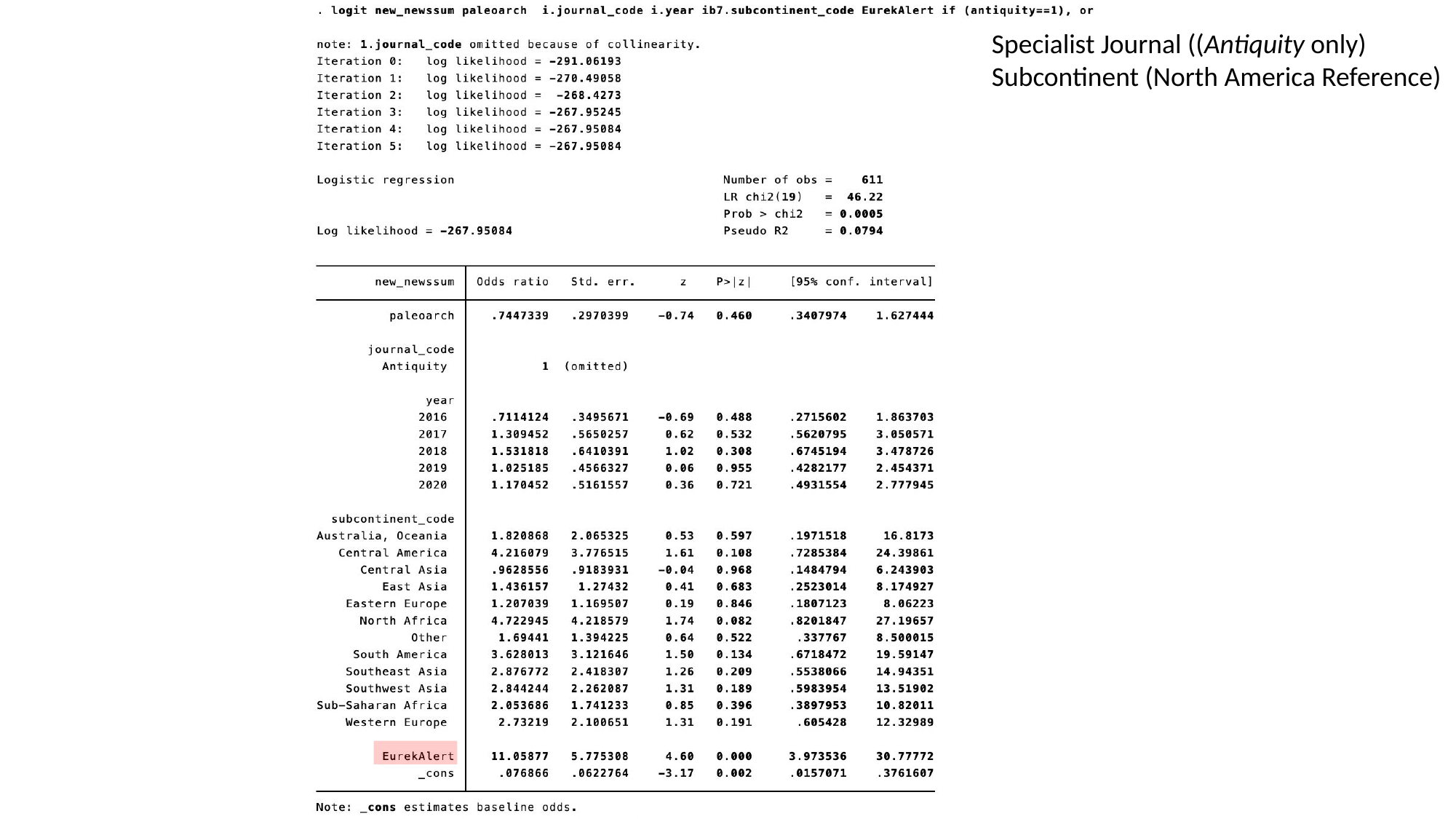

Specialist Journal ((Antiquity only)
Subcontinent (North America Reference)

## Slide 10
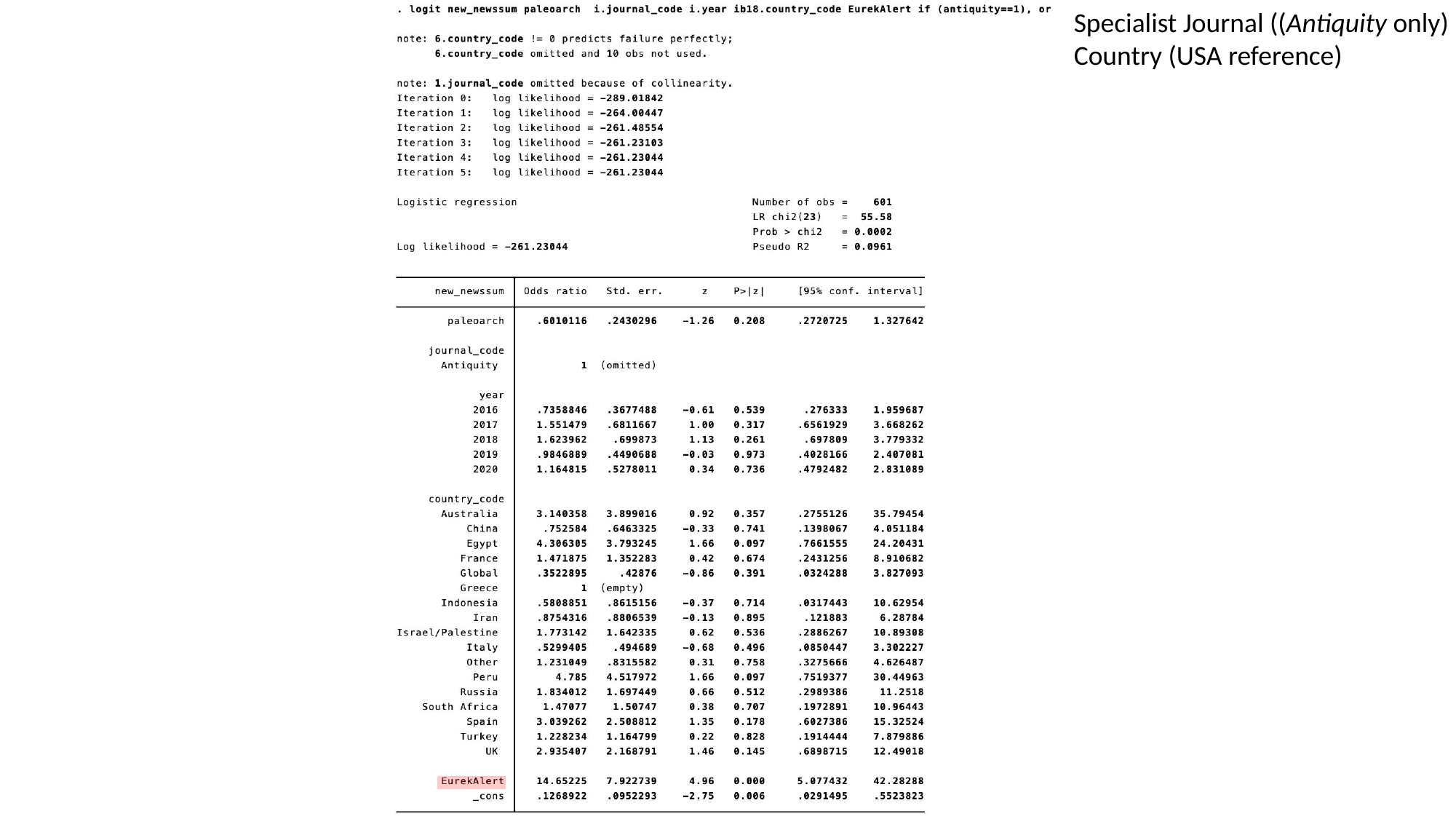

Specialist Journal ((Antiquity only)
Country (USA reference)
